# The prognostic value of ubiquitin/ubiquitin-like-related genes along with immune cell infiltration and clinicopathological features in osteosarcoma

**DOI:** 10.1186/s13018-024-04781-1

**Published:** 2024-06-15

**Authors:** Jian Wen, Lijia Wan, Wenming Chen, Xieping Dong

**Affiliations:** 1 Department of Pain Management, The 2nd Affiliated Hospital, Jiangxi Medical College, Nanchang University, No.1 Minde Road, Nanchang, 330006 Jiangxi China; 2 JXHC Key Laboratory of Digital Orthopaedics, Jiangxi Provincial People’s Hospital, The First Affiliated Hospital of Nanchang Medical College, 152 Aiguo Road, Nanchang, 330006 Jiangxi China; 3 Department of Orthopedics, Pingxiang People’s Hospital, The Sixth Clinical College of Gannan Medical University, Pingxiang, 337000 China; 4 Department of Child Healthcare, Hunan Provincial Maternal and Child Health Hospital, Changsha, 410008 Hunan China

**Keywords:** Ubiquitin/ubiquitin-like-related genes, Bioinformatics, Osteosarcoma, Prognostic model, Immune cell infiltration

## Abstract

**Background:**

Ubiquitin/ubiquitin-like (Ub/UBL)-related genes have been reported to be associated with the survival of osteosarcoma patients but have not yet been systematically explored.

**Methods:**

The prognostic value of Ub/UBL-related genes, immune cell infiltration and clinicopathological features of patients were explored by Cox and LASSO regression analyses. A prognostic model was established and then validated in the GSE21257 dataset. The differential expression of hub genes in osteosarcoma was confirmed by qRT-PCR, western blotting and immunohistochemistry.

**Results:**

Tripartite Motif Containing 8 (*TRIM8*) and Ubiquitin Like With PHD And Ring Finger Domains 2 (*UHRF2*) were screened as genes with prognostic value in osteosarcoma. Kaplan–Meier analysis and scatter plots indicated that patients in the high gene significance score group tended to have a worse prognosis. The concordance index, calibration analysis and receiver operating characteristic analysis suggested that the model had good prediction accuracy and high sensitivity and specificity. Decision curve analysis revealed that patients could obtain greater net benefit from this model. Functional analyses of the differentially expressed genes indicated that they were involved in important functions and pathways. *TRIM8* and *UHRF2* were confirmed to be highly expressed in osteosarcoma cell lines and tissues.

**Conclusions:**

*TRIM8* and *UHRF2* are potential prognostic genes in osteosarcoma, and these results provide insights into the roles of these genes and their implications for patient outcomes.

**Supplementary Information:**

The online version contains supplementary material available at 10.1186/s13018-024-04781-1.

## Background

Osteosarcoma, which occurs mainly in children and adolescents, is the most common primary malignant bone tumor, and it is characterized by the production of osteoid and immature bone by mesenchymal cells or osteogenic progenitor cells [1–4]. Moreover, osteosarcoma is a rare malignancy that accounts for less than 1% of all cancer cases in America [2]. According to the SEER 18 database, the incidence of osteosarcoma between 2000 and 2014 was 3.3 per million [1]. There are two incidence peaks in different age groups: one occurs in adolescence, and the other occurs in individuals older than 60 years of age [2]. Although the incidence of osteosarcoma is low, it is a lethal tumor with high mortality and metastasis rates. The 5-year relative survival rate decreases with increasing age. In the 0–9 year age group, the survival rate is 71.8%, and the survival rate decreases to 33.1% in elderly patients (> 60 years) [1]. Moreover, the survival rate is as low as 20% in patients with metastatic disease [5–7]. Although great progress has been made in the treatment of osteosarcoma, there has been limited improvement in patient survival since the 1980s, when standard therapy was established [8]. Therefore, it is necessary to further explore the prognostic factors of osteosarcoma; such studies can not only contribute to the understanding of osteosarcoma but also reveal potential therapeutic targets of osteosarcoma.

Ub/UBL protein modification is an important posttranslational modification that enables cells to dynamically react to intracellular or environmental changes [9, 10]. The processes by which these two modifications are mediated are similar and reversible. When catalyzed by specific enzymes, ubiquitin and small ubiquitin-like modifiers are added to a substrate, signals are transduced, and a series of subsequent molecular events are triggered. Many vital cellular functions, such as DNA repair, cell cycle progression, cell proliferation and cell apoptosis, are regulated in this way [10, 11]. Moreover, dysregulation of these functions is closely related to cancer [9, 12–18]. More importantly, several Ub/UBL-related genes have recently been shown to have prognostic value in osteosarcoma [19–22]. The ubiquitin-like protein FAT10 has been identified as a promoter of osteosarcoma growth [20, 23]. Zhang D et al. [24] demonstrated that downregulation of Ubiquitin-Specific Protease (USP) 22 inhibits proliferation, invasion, and epithelial-mesenchymal transition in osteosarcoma cells. Sévère et al. [25] showed that targeting the E3 ubiquitin ligase Casitas B-lineage lymphoma inhibits osteosarcoma cell growth and survival, and reduces tumorigenesis. Additionally, USP9X [26], USP39 [27], USP7 [28], the E3 ubiquitin ligase Rlim [29] and MDM2 [30] have been implicated in the tumorigenesis, progression, and metastasis of osteosarcoma.

Therefore, in this study, we used bioinformatics methods to explore the prognostic value of Ub/UBL-related genes in osteosarcoma. The prognostic value of immune cell infiltration and clinicopathological features was also investigated to improve the prediction accuracy and stability of the prognostic model from different perspectives. Although many prognostic biomarkers, even several Ub/UBL-related genes, have been identified in osteosarcoma, the role of other Ub/UBL-related genes in the prognosis of patients with osteosarcoma has not been investigated. Therefore, we explored the prognostic value of all the Ub/UBL-related genes that have been reported to date. Our study not only provides further clarity about the roles of Ub/UBL-related genes in the prognosis of patients with osteosarcoma but also serves to complement existing predictive models.

## Methods

### Data collection

GDC TARGET-OS RNA-seq count data (n = 88), phenotype data (n = 524) and survival data (n = 288) together with GTEX normal tissue RNA-seq count data (UCSC Toil RNA-seq Recompute) were downloaded from the UCSC Xena website (https://xenabrowser.net/hub/). Patients for whom overall survival (OS) data or OS time data were missing were excluded. After screening, the RNA expression and clinical data of 84 osteosarcoma patients, such as age, sex, race, ethnicity, primary site, and metastasis status, were extracted for further research. Moreover, RNA expression data from 379 normal subjects in the GTEX database were extracted (data from the same subject were merged by the mean). Then, RNA expression data from 84 osteosarcoma patients and 379 normal subjects were merged, and the data were transformed to transcripts per million (TPM) for further analysis. The formula was as follows: $TPM = \frac{{q_{i} /l_{i} }}{{\sum\limits_{{{\text{i}} = 1}}^{n} {(q_{i} /l_{i} )} }} \times 10^{9}$, where* q*_*i*_ denotes reads mapped to transcripts, *l*_*i*_ denotes the transcript length, and $\sum\limits_{i = 1}^{{\text{n}}} {(q_{i} /l_{i} )}$ denotes to the sum of mapped reads to transcripts normalized by transcript length [31].

The GSE21257 dataset [32], which contains the largest sample size of osteosarcoma bulk RNA sequence data available in the Gene Expression Omnibus (GEO) database (https://www.ncbi.nlm.nih.gov/geo/), was used as an external validation cohort. Therefore, the expression and clinical data of 53 osteosarcoma patients, which were submitted on Apr 08, 2010 by the Centre for Molecular Medicine Norway, were downloaded from the GEO website (https://www.ncbi.nlm.nih.gov/geo/query/acc.cgi?acc=GSE21257) on February 10, 2022. RNA expression data of 53 osteosarcoma patients were analyzed by the Illumina human-6 v2.0 expression beadchip (using nuIDs as identifiers), transformed by the variance stabilizing transformation (vst) algorithm and subsequently normalized by robust spline normalization.

A collection of 1344 Ub/UBL-related genes was downloaded from the Integrated Database of Regulators for Ubiquitin and Ubiquitin-like Conjugation Database (iUUCD) (http://iuucd.biocuckoo.org/). Subsequently, 984 common Ub/UBL-related genes between the merged expression data and GSE21257 expression data were selected for further analysis.

### Identification of genes with prognostic significance by Cox and LASSO regression analyses.

Cox regression analysis is a popular method that is commonly used in survival analyses. Univariate Cox regression analysis is used to evaluate the impact of a single predictor variable on survival time. In contrast, multivariate Cox regression simultaneously considers multiple predictor variables, adjusting for other covariates, to assess their independent effects on survival time. Least absolute shrinkage and selection operator (LASSO) regression analysis is a linear regression method that incorporates regularization to select relevant predictors by penalizing the absolute size of coefficients. This approach aids in feature selection and mitigates issues such as overfitting and multicollinearity that are often encountered in multivariate Cox regression analysis.

In this study, the TARGET-OS cohort was used as a training set. The “survival” package and Cox regression analysis were used to explore the prognostic value of the 984 common genes in the training set [33]. Genes with a *P* value < 0.05 in the univariate Cox regression analysis were incorporated into the subsequent multivariate Cox regression analysis. Multivariate regression analysis revealed genes with a *P* value < 0.05 to be independent prognostic indicators for osteosarcoma. Finally, LASSO regression analysis with 10 cross validations was performed to further screen genes with prognostic value [34].

### Gene significance score calculation and survival analysis

The gene significance score for each selected gene was calculated by multiplying the expression value (high-throughput sequencing data using TPM data, array data using normalized gene expression data) by its coefficient value. Then, by summing the scores of all the selected genes, we obtained a gene significance score for each sample. The formula was as follows: $significance\;Score = \sum\nolimits_{i = 1}^{n} {\left( {Exp_{{gene_{i} }} \times coefficient_{{gene_{i} }} } \right)}$. Thereafter, the samples were stratified into low- and high-score groups according to the median gene significance score. Then, KM survival analysis was performed and scatter plots and heatmaps were generated to explore the characteristics of the patents in the two groups.

### Infiltration score calculation and screening

The “MCPcounter” package was used to calculate the infiltration scores of fibroblasts, endothelial cells and 8 immune cell types at the tumor site [35]. Then, the gene significance score, cell infiltration score of 10 cell types and clinicopathological features were screened by univariate (*P* value < 0.05) and multivariate (*P* value < 0.05) Cox regression analyses. Indicators with *P* values < 0.05 were selected as the final indicators for the prognostic model.

### Establishment of a prognostic model and its assessment with the training set

A Cox proportional hazards model was established according to the final indicators and then visualized by a nomogram. The concordance index (C-index), calibration analysis, time-dependent receiver operating characteristic (ROC) analysis and decision curve analysis (DCA) were performed to evaluate the prediction accuracy and discriminatory capacity of the model in the training set.

### Validation of the model in an independent external set

With GSE21257 as the validation set, KM analysis was performed with the high- and low-significance score groups (stratified by the median). The characteristics of the patients in different groups were explored by scatter plots and heatmaps. Moreover, the C-index, calibration analysis, time-dependent ROC analysis and DCA were also employed to evaluate the prediction accuracy and discriminatory capacity of the model.

### Gene expression profile and PPI network analysis of the selected genes

Patients were stratified into low- and high-score groups according to the median gene significance score. Then, the expression profiles of the prognostic genes in different score groups were explored in both the training and validation sets to determine whether they were differentially expressed. Potential protein–protein interaction (PPI) network analysis of the genes was performed via the STRING website, with an interaction score ≥ 0.4 (https://cn.string-db.org/).

### Identification of DEGs between the high- and low-gene significance score groups and functional analysis

To preliminarily explore the possible mechanism underlying the difference in prognosis between the low- and high-significance score groups, the “DESeq2” package was used to identify DEGs with adjusted *P* values < 0.05 and |log_2_fold change|> 1 in the TARGET-OS cohort (high-score group versus low-score group). Then, GO and KEGG clustering analysis and gene set enrichment analysis (GSEA) were used to investigate the functional enrichment of the DEGs. Finally, PPI network analysis was performed to explore the interactions among the proteins that were encoded by the DEGs with the STRING website with interaction scores ≥ 0.4 (https://cn.string-db.org/).

### Validation of the hub gene expression in cell lines by real-time fluorescent quantitative PCR (qRT-PCR) and Western blotting

#### Cell culture

The normal human osteoblast cell line hFOB1.19 was purchased from the Shanghai Institute of Biochemistry and Cell Biology (Shanghai, China, catalog number: GNHu14). The MG63 cell line was purchased from iCell Bioscience Inc. (Shanghai, China, catalog number: iCell-h140). The 143B cell line was purchased from FuHeng BioLogy (Shanghai, China, catalog number: FH0438). All the cells were cultured in Dulbecco's modified Eagle’s medium/nutrient mixture F-12 (DMEM/F-12, Gibco, United States, catalog number: 11320033) supplemented with 10% fetal bovine serum (FBS, Gibco, catalog number: 26010074) and 1% penicillin/streptomycin (Solarbio, Beijing, China, catalog number: P1400). The human osteoblast cell line hFOB 1.19 was cultured at 34 °C with 5% carbon dioxide, and the osteosarcoma cell lines were cultured at 37 °C with 5% carbon dioxide in a humidified atmosphere.

#### qRT-PCR

Real-time fluorescent quantitative PCR (qRT-PCR) was used to measure the mRNA expression of Tripartite Motif Containing 8 (*TRIM8*) and Ubiquitin Like With PHD And Ring Finger Domains 2 (*UHRF2*) in the osteoblast and osteosarcoma cell lines. TRIzol reagent (CWBIO, Beijing, China, catalog number: CW0580S) was used to extract total RNA from the osteoblast and osteosarcoma cells. Then, cDNA was reverse transcribed from 1 μg of the extracted RNA using HiScript II Q RT SuperMix for qPCR (+ gDNA wiper) (Vazyme, Nanjing, China, catalog number: R223-01). The special primers (Table 1) and ChamQ Universal SYBR qPCR Master Mix (Vazyme, Nanjing, China, catalog number: Q711-02) were used to perform qRT-PCR with the CFX Connect™ fluorescent quantitative PCR detection system (Bio-Rad Laboratories (Shanghai) Co., Ltd., Shanghai, China) with the following steps: 95 °C for 10 min, 95 °C for 10 s, 58 °C for 30 s, and 72 °C for 30 s (40 cycles). β-actin was used as an internal control, and the 2^−ΔΔCT^ method was used for data analysis. The experiment was repeated three times.

**Table 1:** The sequences of the primers that were used in the RT-PCR experiments

Gene	Sequence (5′ to3′)
*TRIM8*-F	GACGGAGGATGTCAGCTTCA
*TRIM8*-R	TCAGGTGGCCGATCTTAGTG
*UHRF2*-F	ATTCTTGCTCCTGTCGTGTATGT
*UHRF2*-R	CTTGAGTCTTTCACCAGCCTTT
*β-actin*-F	TGGCACCCAGCACAATGAA
*β-actin*-R	CTAAGTCATAGTCCGCCTAGAAGCA

#### Western blotting analysis

The protein expression of *TRIM8* and *UHRF2* in the osteoblast and osteosarcoma cell lines was quantified by the Western blotting analysis. β-actin was used as an internal control. The cells were lysed with RIPA buffer (Beyotime Biotechnology, Shanghai, China, catalog number: P0013B) supplemented with 2% protease inhibitor (APPLYGEN, Beijing, China, catalog number: P1265) at 4 °C for 30 min. Equal amounts (0.83 μg) of protein from hFOB1.19, MG63, and 143B cells were separated by 10% SDS-PAGE, and transferred to PVDF membranes (Millipore, Darmstadt, Germany, catalog number: IPVH00010), and then, the membranes were blocked with 5% skim milk. The membranes were incubated with the primary antibodies (anti-TRIM8: 1:1000 dilution, Proteintech Group Inc., Rosemont, IL, USA, catalog number: 27463-1-AP; anti-UHRF2: 1:1000 dilution, Affinity Biosciences, Cincinnati, OH, USA, catalog number: DF6930; anti-β-actin: 1:2000 dilution, TransGen Biotech, Beijing, China, catalog number: HC201) overnight at 4 °C. After the membranes were rinsed with TBST buffer three times, they were incubated with an HRP-labeled secondary antibody (1:2000 dilution, Servicebio, Wuhan, China; catalog number: GB23301/GB23303) for 1 h. After the membranes were incubated with the highly sensitive plus ECL luminescent reagent for 2 min, an ultrasensitive multifunctional imager (Tanon-5200, Shanghai, China) was used to visualize the bands.

### Immunohistochemical staining for proteins encoded by prognostic genes

Immunohistochemical staining was used to identify the differentially expressed proteins that were encoded by the prognostic genes between osteosarcoma and normal bone tissues. Paraffin-embedded osteosarcoma tissue sections were obtained from 3 osteosarcoma patients at Jiangxi Provincial People’s Hospital (approved by the Ethics Committee of Jiangxi Provincial People's Hospital (1 August 2022), NO. 2022-059). Immunohistochemical staining of the proteins was performed according to the protocol described below. The sections were dewaxed, hydrated, subjected to antigen retrieval in boiling antigen retrieval buffer (1 mM EDTA, pH 8.0) for 20 min, incubated with 3% hydrogen peroxide, blocked with 5% bovine serum albumin (S12012, Shanghai Yuanye Bio-Technology Co., Ltd, Shanghai, China), incubated with primary antibodies at 4 °C overnight (anti-TRIM8: 1:200 dilution, Proteintech Group Inc.; anti-UHRF2: 1:100 dilution, Affinity Biosciences), and incubated with secondary antibodies for 30 min (1:100 dilution, ZSGB-BIO, Beijing, China; catalog number: ZB-2301). Then, the sections were stained with 3,3'-diaminobenzidine (CWBIO, catalog number: CW0125M), stained with hematoxylin, dehydrated and sealed. Then, the sections were observed under a microscope (CX43, OLYMPUS, Tokyo, Japan). Two randomly chosen fields per Sect. (400 ×) were utilized to determine the positive rate through the IHC Profiler function within ImageJ software. Subsequently, the positive rates of both groups were compared and visualized using GraphPad Prism 8.3.0 for Windows (GraphPad Software, Boston, Massachusetts, USA; www.graphpad.com).

### Statistical analysis

In this study, R software v3.63 was used to process the data and generate charts, and Cytoscape software v3.7.1 was used to visualize the PPI network. Normally distributed continuous data were subjected to statistical analysis using either Student’s t test or one-way ANOVA, while nonnormally distributed continuous data were analyzed using the Mann–Whitney U test. Categorical variables were examined by the chi-square test or Fisher’s exact test. A *P* value less than 0.05 was considered to indicate statistical significance.

## Results

### Clinicopathological features of the osteosarcoma patients who were enrolled in this study

Eighty-four patients from the TARGET-OS database (training set) and 53 osteosarcoma patients from the GSE21257 database (validation set) were included in this study. The baseline characteristics of the two cohorts were similar (Table 2).

**Table 2:** Clinicopathological features of osteosarcoma patients in the different cohorts

Characteristics	Level	TARGET-OS	GSE21257	*P* value	Test
Sample size (n)		84	53		
Age (years), No. (%)	< 18	66 (78.6)	34 (64.2)	0.077	Exact
	≥ 18	18 (21.4)	19 (35.8)		
Sex, No. (%)	Female	37 (44.0)	19 (35.8)	0.376	Exact
	Male	47 (56.0)	34 (64.2)		
Primary site, No. (%)	Femur	38 (45.2)	27 (50.9)	0.611	Exact
	Tibia	21 (25.0)	15 (28.3)		
	Fibula	8 (9.5)	2 (3.8)		
	Others	17 (20.2)	9 (17.0)		
Metastasis, No. (%)	Absent	63 (75.0)	39 (73.6)	0.844	Exact
	Present	21 (25.0)	14 (26.4)		
OS, No. (%)	Alive	57 (67.9)	30 (56.6)	0.205	Exact
	Dead	27 (32.1)	23 (43.4)		
OS.time (Months: median [Q1, Q3])		48.65 [20.63, 69.15]	45.00 [27.00, 94.00]	0.122	Kruskal

### Ub/UBL related genes screened by multiple survival analyses

**Fig. 1:**
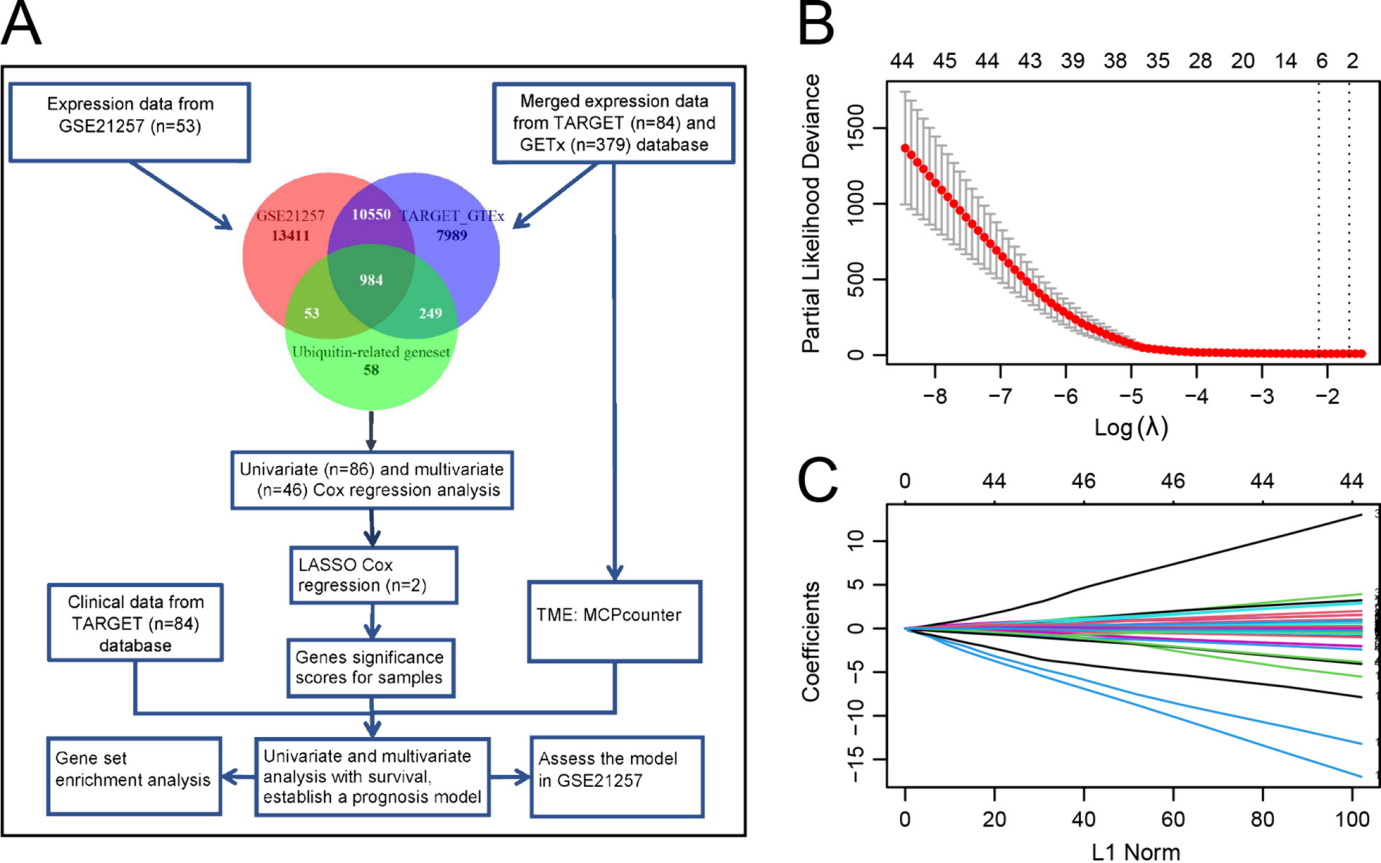
Flowchart of this study and LASSO Cox regression analysis of the prognostic genes. **A** Flowchart of this study. **B** LASSO coefficient profiles for the 46 genes identified by tenfold cross-validation. **C** Partial likelihood deviance with changing log (λ) plotted by LASSO regression in tenfold cross-validation

The overall process of analysis of this study is shown in the flowchart (Fig. 1A). Among the 984 common Ub/UBL-related genes, 86 exhibited a *P* value < 0.05 in the univariate Cox regression analysis, and among these 86 genes, 46 displayed a *P* value < 0.05 in the multivariate Cox regression analysis (Supplementary file 1). Finally, *TRIM8* and *UHRF2* were identified as prognostic hub genes in osteosarcoma by LASSO regression analysis (λ = lambda.1se) (Fig. 1 B-C).

### The prognosis of patients in different gene significance score groups

**Fig. 2:**
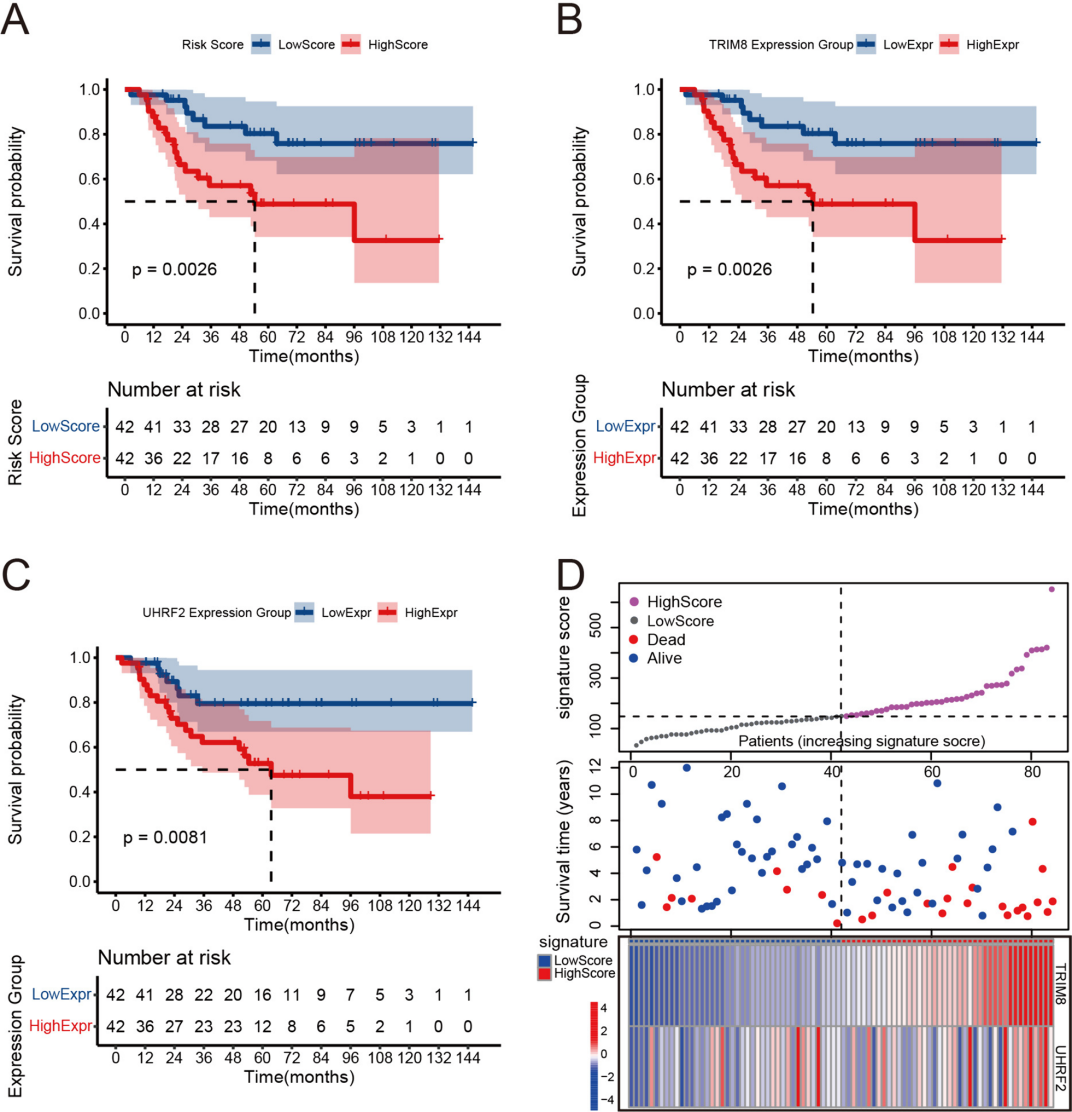
KM analysis and characteristics of patients in different groups of the training set. **A** The KM plot for the high- and low-gene significance groups in the TARGET-OS cohort. **B** The KM plot for the high- and low-expression groups of *TRIM8* in the TARGET-OS cohort. **C** The KM plot for the high- and low-expression groups of *UHRF2* in the TARGET-OS cohort. **D** Characteristics of patients in the high- and low-significance score groups: upper panel: the x-axis refers to samples with increasing gene significance scores, and the y-axis refers to the gene significance scores; middle panel: the x-axis refers to samples with increasing gene significance scores, and the y-axis refers to the survival times of patients; and lower panel: the heatmap for the expression of *TRIM8* and *UHRF2* in samples ranked by the gene significance scores

Patients were stratified into high- and low-gene significance score groups according to the median score (gene significance score = TRIM8 * 0.00426934 + UHRF2 * 0.03053178). The KM plot revealed that the red and blue curves closely overlapped and crossed paths within the first 12 months, but after that point, a clear divergence emerged (Fig. 2A). Consequently, for the sake of result reliability, our subsequent discussion focused primarily on the prognostic value of the model for predicting outcomes beyond 12 months. The KM plot demonstrated that, 12 months after the initial diagnosis, patients in the high-score group exhibited a notably worse prognosis (*P* = 0.0026). Additionally, patients with elevated expression levels of *TRIM8* and *UHRF2* also had worse outcomes, with *P* values of 0.0026 and 0.0081, respectively (Fig. 2B-C). Figure 2D provides insights into the characteristics of patients in both the high- and low-significance score groups. The high-score group had a higher incidence of mortality and shorter overall survival (middle of Fig. 2D). In the lower section of Fig. 2D, the heatmap shows the expression patterns of *TRIM8* and *UHRF2* in the samples, illustrating the increasing gene significance scores. Notably, the expression profile of *TRIM8* closely mirrored the increasing scores that were observed among patients in the training set.

### Further screening of final independent indicators for the prognostic model

The infiltration scores of fibroblasts, endothelial cells and 8 immune cell types in the training and validation sets are provided in Supplementary Files 2, and 3. The gene significance score, metastasis, and monocytic lineage (cell originating from monocytes) [36] infiltration at the tumor site were screened as the final indicators by univariate and multivariate Cox regression analyses (Table 3).

**Table 3:** Screening indicators for the prognostic model by univariate and multivariate Cox analyses

Characteristics	Univariate Cox regression		Multivariate Cox regression	
	Hazard.Ratio(95% CI)	*P*.Value	Hazard.Ratio(95% CI)	*P*.Value
Metastasis	4.76(2.22–10.22)	**0.000061**	3.8(1.7–8.48)	**0.001**
Gene significance score	0.30(0.13–0.69)	**0.005**	0.29(0.12–0.66)	**0.003**
Monocytic lineage	0(0–0)	**0.012**	0(0–0)	**0.043**
B lineage	0(0–4.59E + 30)	0.237	#N/A	#N/A
CD8 + T cells	0(0–3.37E + 211)	0.296	#N/A	#N/A
Cytotoxic lymphocytes	0(0–6.86E + 32)	0.587	#N/A	#N/A
Endothelial cells	1.08E + 49 (0–4.02E + 235)	0.606	#N/A	#N/A
Fibroblasts	1.4(0.27–7.25)	0.686	#N/A	#N/A
Myeloid dendritic cells	0(0–2.71E + 286)	0.218	#N/A	#N/A
Neutrophils	0(0-Inf)	0.760	#N/A	#N/A
NK cells	0(0-Inf)	0.492	#N/A	#N/A
T cells	0(0-Inf)	0.855	#N/A	#N/A
Age	1(1–1)	0.976	#N/A	#N/A
Gender	0.71(0.34–1.52)	0.382	#N/A	#N/A
Ethnicity (Hispanic or Latino)				
Not Hispanic or Latino	0.36(0.13–1.03)	0.056	#N/A	#N/A
Unknown	0.61(0.12–3.20)	0.557	#N/A	#N/A
Primary site (Femur)				
Fibula	0.69(0.16–3.01)	0.619	#N/A	#N/A
Tibia	0.30(0.09–1.03)	0.056	#N/A	#N/A
others	0.81(0.32–2.08)	0.663	#N/A	#N/A
Race (Asian)				
White	0.75(0.17–3.29)	0.699	#N/A	#N/A
Black or African American	0.30(0.03–3.40)	0.334	#N/A	#N/A
Unknown	2.29(0.44–11.92)	0.326	#N/A	#N/A

95% CI: 95% confidence interval; #N/A: Not applicable

Bold values indicate statistical significance of *P*-values

### Establishment of a prognostic model and its evaluation in the training set

**Fig. 3:**
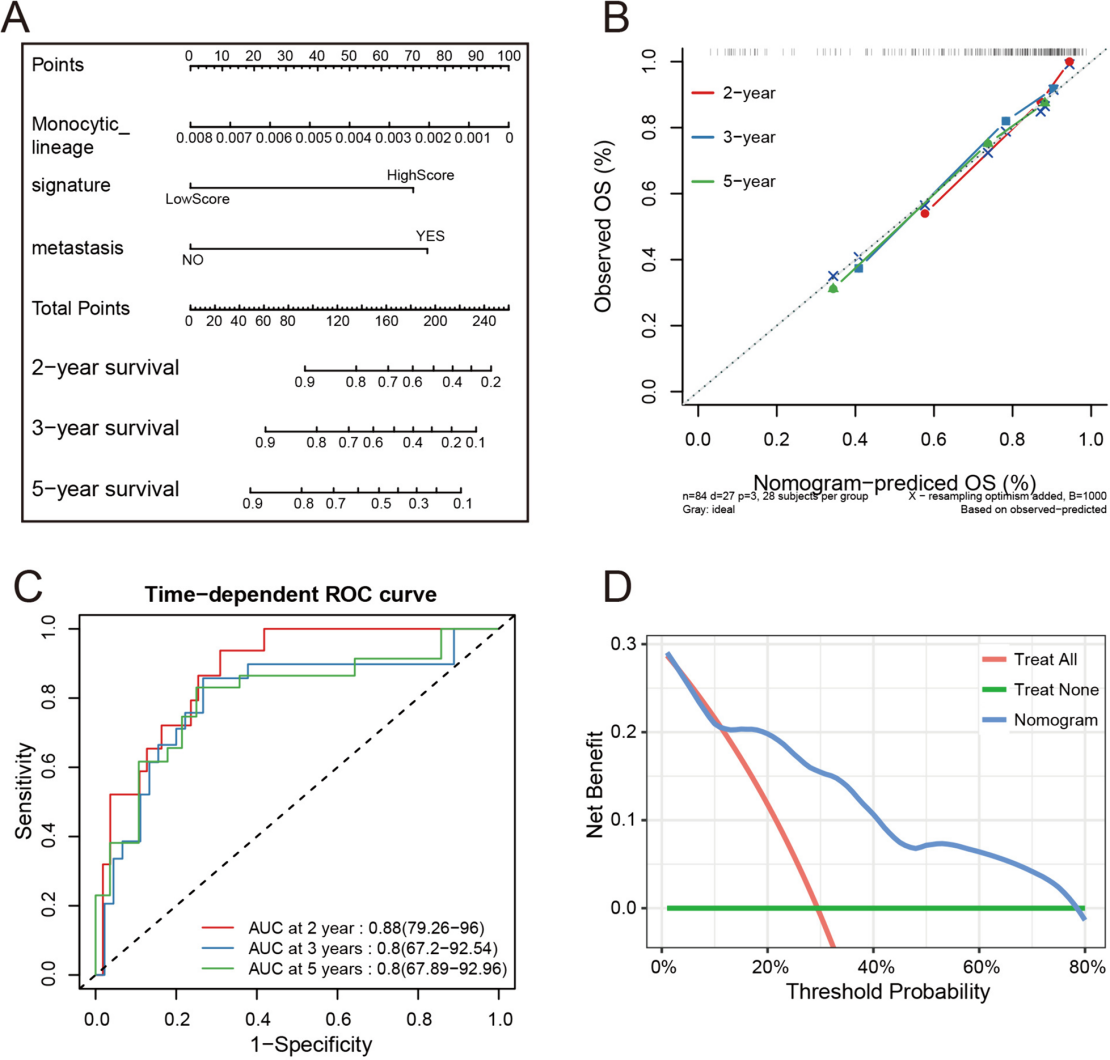
Visualization and evaluation of the model in the training set. **A** The nomogram of the Cox proportional hazards model. **B** The 2-, 3-, and 5-year calibration analysis of the model in the training set. **C** Time-dependent ROC curve analysis of the model in the training set. **D** The 3-year DCA in the training set

A Cox proportional hazards model was established by the final indicators and visualized by a nomogram, which could be used to predict the survival probability of a patient by summing the points of the 3 final indicators (Fig. 3A). The higher the total points are, the lower the survival probability. The C-index of the model in the training set was 0.797 (95% CI: 0.751–0.843). Calibration analysis (Fig. 3B) revealed that the predicted 2-, 3-, and 5-year overall survival rates were highly consistent with the overall survival observed. The C-index and the calibration analysis both indicated good predictive accuracy of the model in the training set. The time-dependent ROC analysis indicated that the area under the curve (AUC) values for the 2-, 3-, and 5-year predictions of the model in the training set were 0.88, 0.80 and 0.80, respectively, which suggested good predictive specificity and sensitivity of the model (Fig. 3C). The DCA of the model for 3-year prediction showed a high net benefit of the nomogram (Fig. 3D).

### Validation of the model in the GSE21257 dataset

**Fig. 4:**
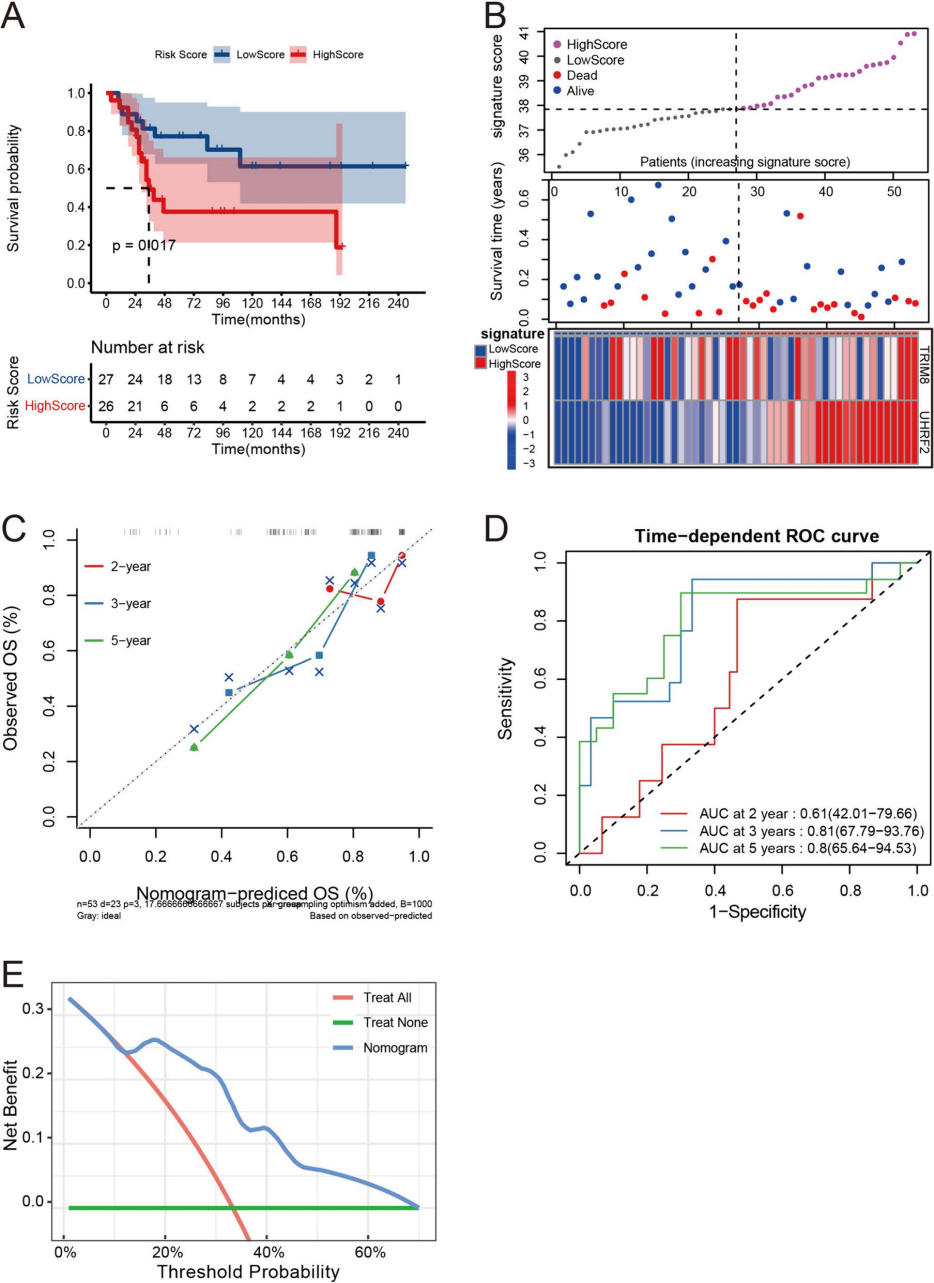
Evaluation of the model in the validation set. **A** The KM plot of the high- and low-gene significance score groups in the GSE21257 dataset. **B** The characteristics of patients in the high- and low-gene significance score groups in the GSE21257 dataset: upper panel: the x-axis refers to samples with increasing gene significance scores, and the y-axis refers to the gene significance scores; middle panel: the x-axis refers to samples with increasing gene significance scores, and the y-axis refers to patient survival times; lower panel: heatmap for the expression of *TRIM8* and *UHRF2* in samples ranked by gene significance scores. **C** The 2-, 3-, and 5-year calibration analysis of the model in GSE21257. **D** Time-dependent ROC curve analysis of the model in GSE21257. **E** 3-year DCA in GSE21257

KM analysis of the GSE21257 dataset revealed that patients in the high-significance score group had poorer outcomes than those in the low-significance score group after 2 years (Fig. 4A). Notably, a similar pattern was observed where the two curves in the Kaplan–Meier plot closely converged and intersected between 6 and 18 months, mirroring the findings in the training set. Fortunately, the prognostic value of the model after 18 months was not affected. Figure 4B provides a graphical depiction of patient characteristics in the high- and low-score groups through a scatter plot and heatmap. Patients from the GSE21257 dataset in the high-score group exhibited a higher frequency of mortality and shorter overall survival. The heatmap in Fig. 4B illustrates that the overall expression patterns of *TRIM8* and *UHRF2* in patients were consistent with the increasing trend of the gene significance score. Notably, compared with the heatmap from the training set, the expression profile of *UHRF2* was more consistently aligned with the increasing scores that were observed for the patients from the GSE21257 dataset.

The C-index in the validation set was 0.708 (95% CI: 0.65–0.766), which was slightly lower than that in the training set. Calibration analysis (Fig. 4C) revealed that the predicted overall survival from 2 to 5 years became increasingly more consistent with the observed overall survival. Moreover, the predicted 3- and 5-year overall survival rates were highly consistent with the overall survival rates observed. The C-index and calibration analysis both suggested the high predictive accuracy of the model in the validation set. The time-dependent ROC analysis showed that the AUC values for the 2-, 3-, and 5-year predictions of the model in the training set were 0.61, 0.81 and 0.80, respectively (Fig. 4D). The 2-year AUC in the validation set was much lower than that in the training set, but the 3-year and 5-year AUCs in the validation set were just as good as those in the training set; these results suggested the stable and reliable predictive value of the model for 3- and 5-year survival. The DCA of the model for 3-year prediction in the validation set also indicated a higher net benefit of the nomogram (Fig. 4E).

### Differential expression analysis and PPI network analysis of *TRIM8* and *UHRF2*

**Fig. 5:**
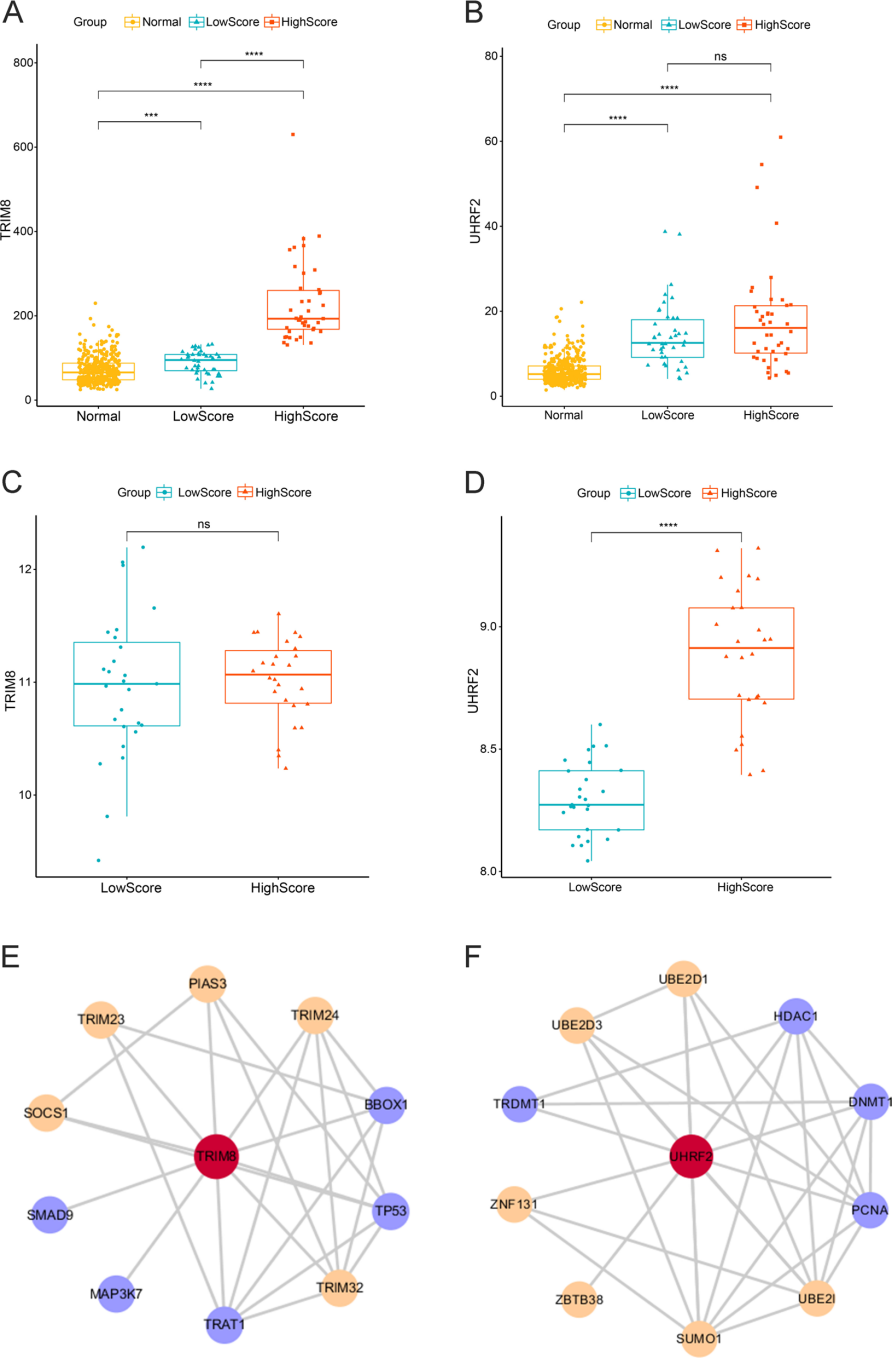
Expression profiles of *TRIM8* and *UHRF2* in the normal and tumor groups and PPI network analysis of TRIM8 and UHRF2. Expression of *TRIM8* (**A**) and *UHRF2* (**B**) in the normal, low- and high-gene significance score groups in the training set: the x-axis refers to the group, and the y-axis refers to the RNA expression. Expression of *TRIM8* (**C**) and *UHRF2* (**D**) in the low- and high-gene significance score groups in the validation set: the x-axis refers to the group, and the y-axis refers to the RNA expression. PPI network analyses of TRIM8 (**E**) and UHRF2 (**F**): orange nodes refer to the Ub/UBL-related proteins. (Significance level: no significance (ns), *P* ≥ 0.05; *, *P* < 0.05; **, *P* < 0.01; ***, *P* < 0.001, ****, *P* < 0.0001.)

*TRIM8* and *UHRF2* expression in the tumor group was significantly higher than that in the normal group (Fig. 5A-B). In the training set, *TRIM8* exhibited a notably higher expression level in the high score group, while in the validation set, *UHRF2* displayed a significantly higher expression in the high-score group (Fig. 5A, D). Although the average expression of *UHRF2* in the training set and that of *TRIM8* in the validation set were higher in the high-score group, the differences were not statistically significant (Fig. 5B, C). The PPI networks (Fig. 5E-F) showed that TRIM8 mainly interacted with SMAD9, MAP3K7, TP53, BBOX1, TRAT1, TRIM23, SOCS1, PIAS3, TRIM24, and TRIM32, while UHRF2 mainly interacted with ZBTB38, ZNF131, UBE2I, UBE2D3, UBE2D2, SUMO1, PCNA, HDAC1, TRDMT1, and DNMT1. The orange nodes in Fig. 5E-F are proteins involved in the Ub/UBL processes.

### Identification of DEGs between the high- and low-gene significance score groups and functional analysis

**Fig. 6:**
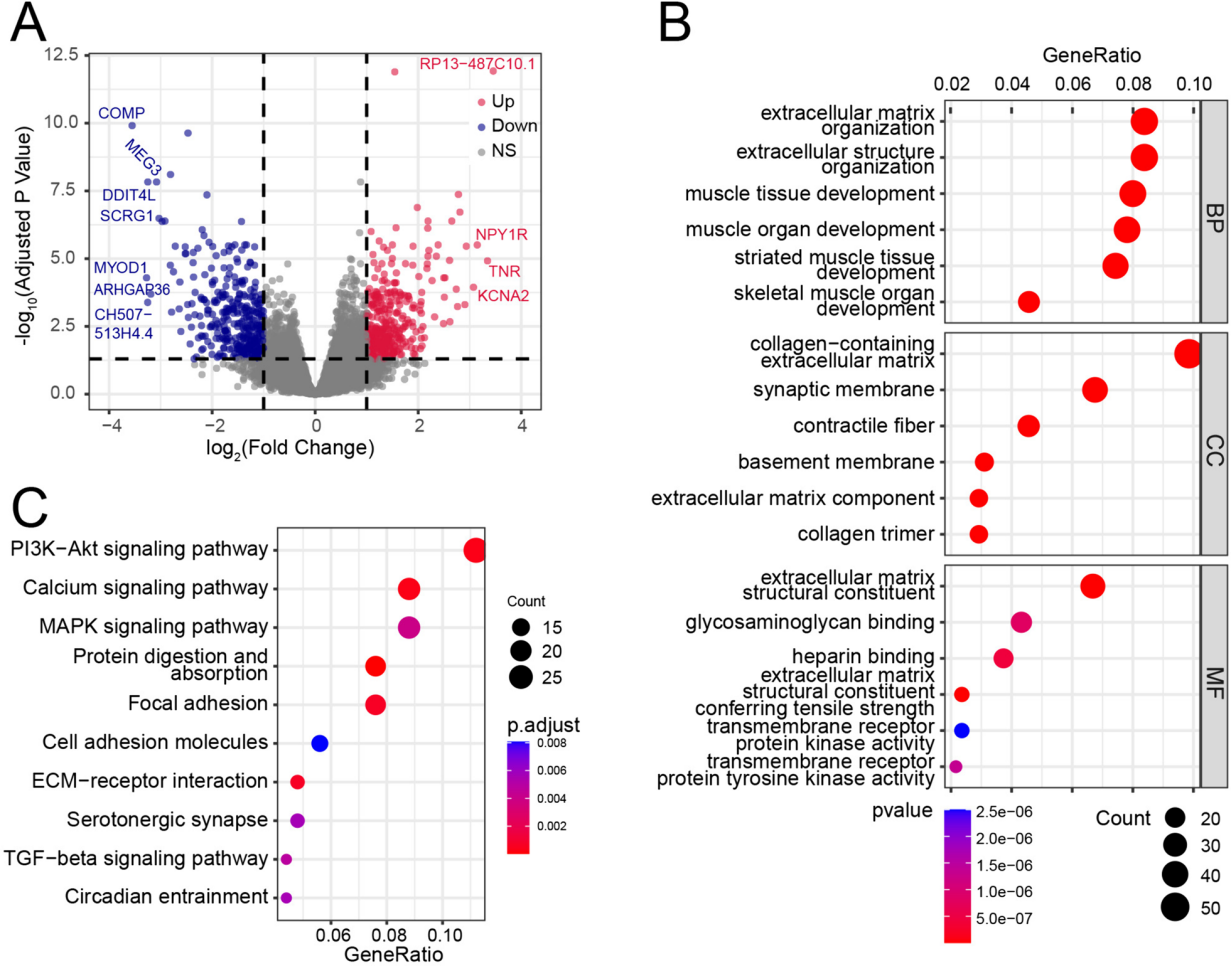
Identify DEGs between the high- and low-gene significance score groups and gene enrichment analysis. **A** Volcano plot of the DEGs between the high- and low-gene significance score groups. **B** Dot plot for GO clustering of the DEGs. **C** Dot plot for KEGG clustering of the DEGs

**Fig. 7:**
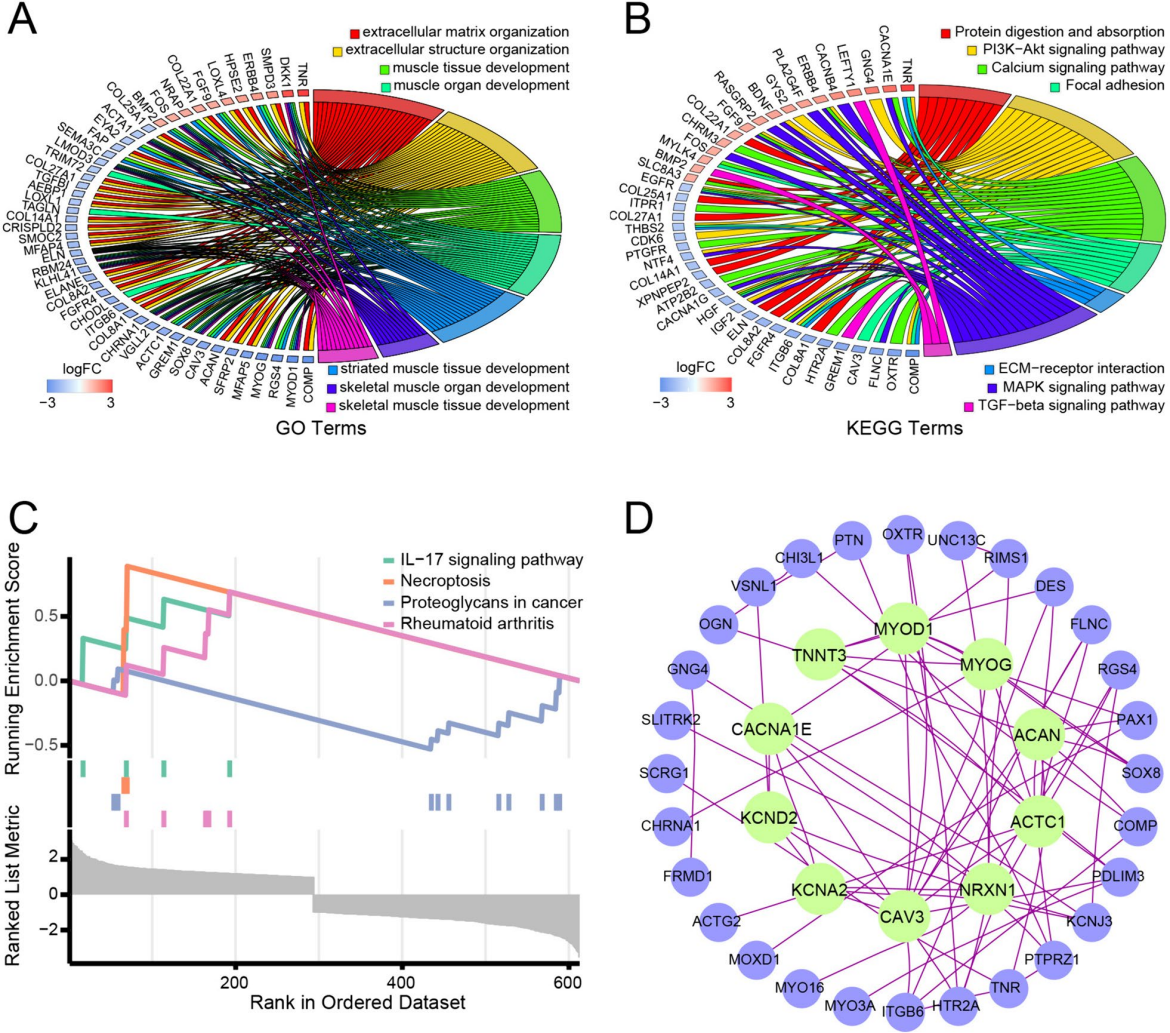
Functional analysis of the DEGs. **A** Chord plot for the 7 top clustered BPs. **B** Chord plot for the 7 top clustered KEGG pathways. **C** The four top enriched pathways according to GSEA. **D** PPI network analysis of the DEGs

There were 917 DEGs between the high and low gene significance score groups (adjusted *P* value < 0.05 and |log2fold change|> 1) (Supplementary file 4). The colored dots in the volcano plot (Fig. 6A) are genes with adjusted *P* values < 0.05 and |log_2_fold change|> 1. The processes clustered by GO clustering were mainly associated with muscle and extracellular matrix organization. The top 3 clustered biological processes (BP) of the DEGs ranked by generatio were extracellular matrix organization, extracellular structure organization, and muscle tissue development. The top 3 cellular components (CC) were collagen-containing extracellular matrix, synaptic membrane, and contractile fiber, and the top 3 molecular functions (MF) were extracellular matrix structural constituent, glycosaminoglycan binding, and heparin binding (Fig. 6B). Moreover, pathways clustered by KEGG were mainly associated with signal transduction (Fig. 6C). The top 5 pathways of the DEGs clustered by KEGG enrichment were the PI3K-Akt signaling pathway, calcium signaling pathway, MAPK signaling pathway, protein digestion and absorption, and focal adhesion. The top DEGs, ranked by the absolute value of logFC, which were involved in the top GO and KEGG processes are shown in Fig. 7A-B. GSEA revealed that the IL-17 signaling pathway, necroptosis, proteoglycans in cancer, and rheumatoid arthritis were the top 4 enriched pathways, among which proteoglycans in cancer were downregulated and the other three were upregulated in the high-score group (Fig. 7C). The PPI network of proteins encoded by the DEGs is shown in Fig. 7D. CAV3, NRXN1, ACTC1, ACAN, MYOG, MYOD1, TNNT3, CACNA1E, KCND2 and KCNA2 were the most connected proteins in the network. Most of them also participate in the processes clustered by GO and KEGG analyses.

### qRT-PCR and Western blotting analysis of the expression profiles of the hub genes in cell lines

**Fig. 8:**
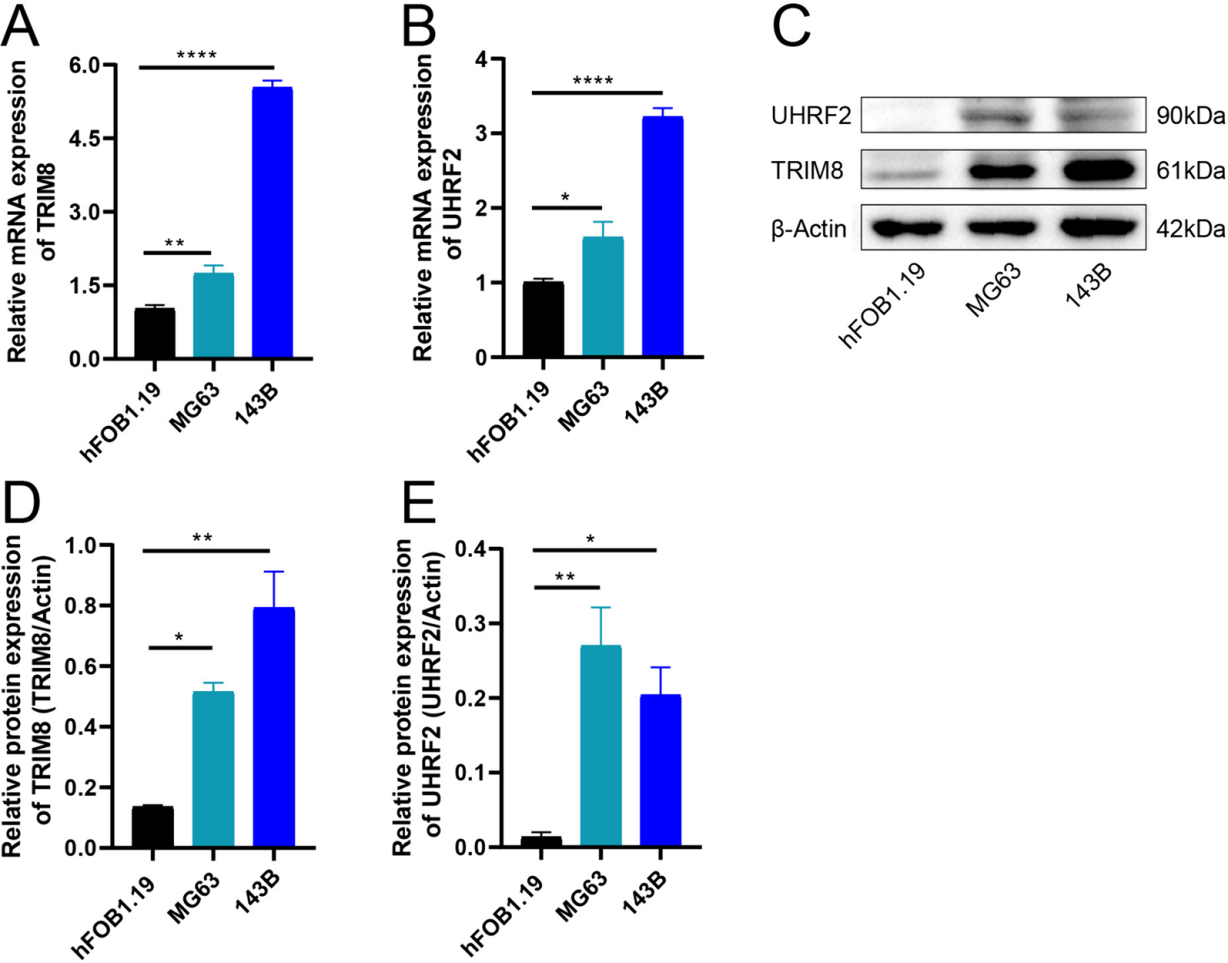
The mRNA and protein expression of *TRIM8* and *UHRF2* in cell lines. The mRNA expression of *TRIM8* (**A**) and *UHRF2* (**B**) relative to that of β-actin in osteosarcoma and osteoblast cells by qRT-PCR (2^−ΔΔCT^ method, mean ± standard error of the mean). **C** Western blotting analysis of *TRIM8* and *UHRF2* expression. The protein expression of *TRIM8* (**D**) and *UHRF2* (**E**) relative to that of β-actin in osteosarcoma and osteoblast cells was determined by Western blotting analysis (mean ± standard error of the mean)

qRT-PCR indicated that the mRNA expression of *TRIM8* and *UHRF2* in the 143B and MG63 cell lines was significantly higher than that in osteoblasts (*P* value < 0.05) (Fig. 8A-B). Western blotting analysis further confirmed that the protein expression of *TRIM8* and *UHRF2* in the 143B and MG63 cell lines was notably higher than that in osteoblasts (Fig. 8C-E). Our results in cell lines were consistent with the in silico results above.

### Immunohistochemical staining for TRIM8 and UHRF2

**Fig. 9:**
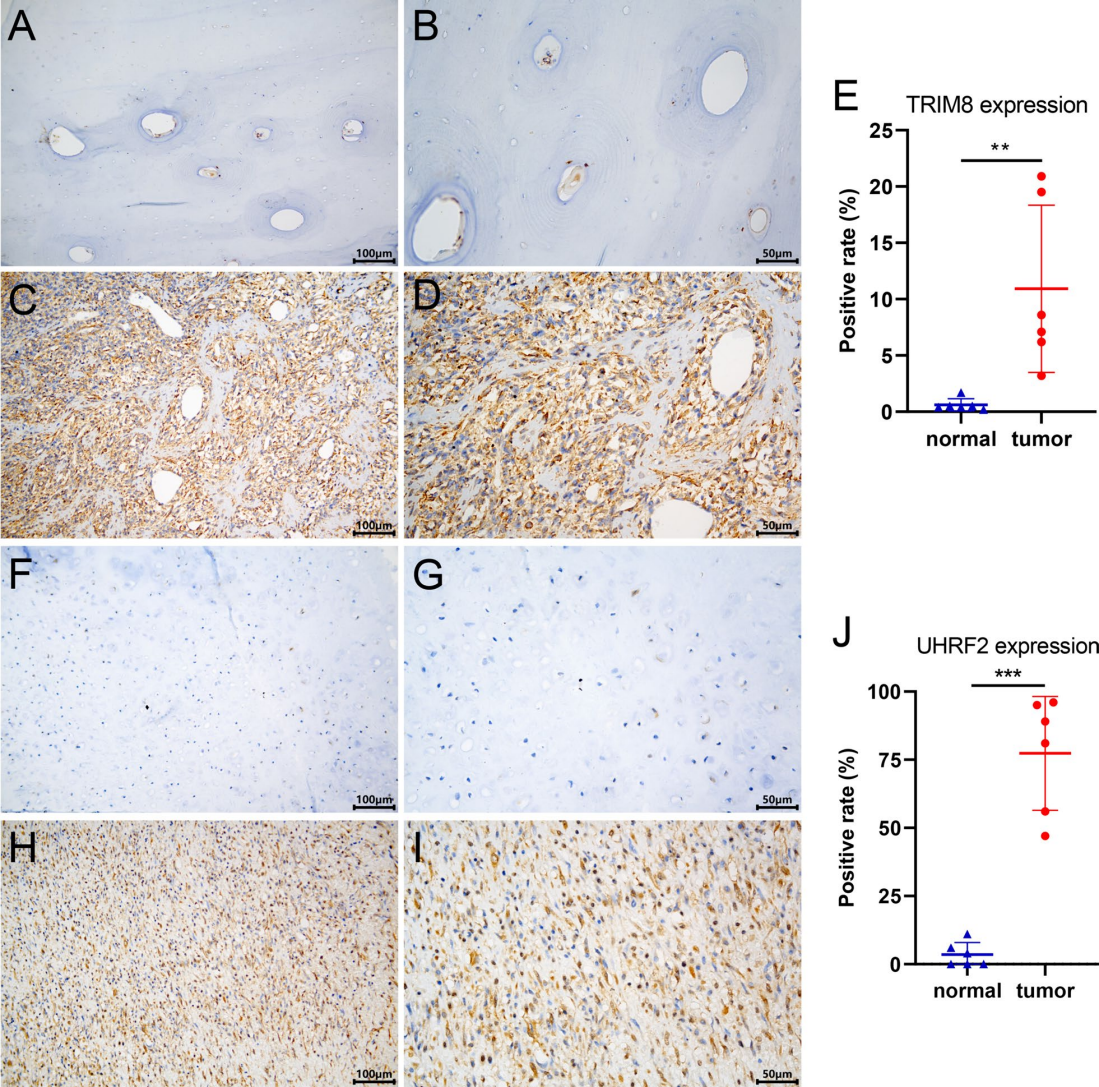
Immunohistochemical staining for TRIM8 and UHRF2 in osteosarcoma and normal bone tissues. Immunohistochemical staining for TRIM8 in normal bone (**A**, **B**) and osteosarcoma (**C**, **D**) tissues at 200 × and 400 × magnification, respectively. **E** Scatter plot of the positive rate of TRIM8. Immunohistochemical staining for UHRF2 in normal bone (**F**, **G**) and osteosarcoma (**H**, **I**) tissues at 200 × and 400 × magnification, respectively. **J** Scatter plot of the positive rate of UHRF2. Significance level: no significance (ns), *p* ≥ 0.05; *, *p* < 0.05; **,* p* < 0.01; ***, *p* < 0.001, ****, *p* < 0.0001)

Immunohistochemical staining for TRIM8 (Fig. 9A-E) and UHRF2 (Fig. 9F-J) indicated that these proteins were highly expressed in osteosarcoma tissues.

## Discussion

To date, progress in improving long-term survival in elderly patients with metastatic osteosarcoma has been disappointing. Accumulating evidence shows that Ub/UBL-related genes play important roles in the survival of patients with osteosarcoma [19–22].

In this study, *TRIM8* and *UHRF2* were identified as the Ub/UBL-related genes that were most strongly associated with survival by Cox and LASSO regression analyses, and the gene significance scores were calculated according to these genes. Since the coefficient values of *TRIM8* and *UHRF2* were positive, a higher expression of *TRIM8* and *UHRF2* would lead to a higher significance score for the patient. In addition to the gene significance score, metastasis and monocytic lineage infiltration at the tumor site were also identified as prognostic indicators by univariate and multivariate Cox regression analyses. KM analysis and the scatterplot both showed that patients in the high-gene significance score group tended to exhibit a poorer prognosis in both the training and validation sets, indicating the robust discriminatory capacity of the gene significance score. In the training set, patient prognosis varied significantly based on high and low expression levels of *TRIM8* and *UHRF2*, with KM analysis yielding a *P* value of less than 0.01 for both. Notably, the KM curve of the risk score mirrors that of *TRIM8*, primarily due to the high predictive efficacy of *TRIM8* within this dataset and the limited sample size of the training set. Although the C-index and calibration analysis performed slightly less effectively in the validation set than in the training set, they still exhibited a commendable level of predictive accuracy (C-index > 0.7). Moreover, it is important to note that a reduction in model accuracy in the validation set compared to the training set is a common and expected occurrence. The time-dependent ROC analysis suggested the high sensitivity and specificity of the model in both the training and validation sets, especially for 3- and 5- year predictions (AUC ≥ 0.8). The DCA also indicated that patients in both the training and validation sets could obtain greater net benefits from the model than other strategies. In summary, our model was successfully validated in an independent cohort, suggesting good prediction accuracy and discriminatory capacity of the model.

Differential expression of the two genes in the normal, high- and low-gene significance score groups indicated that *TRIM8* and *UHRF2* were highly expressed in osteosarcoma patients and that the higher the expression was, the worse the prognosis. Moreover, the differential expression of *TRIM8* and *UHRF2* in the normal and osteosarcoma groups was also successfully validated in cell lines by qRT-PCR and Western blotting and in human tissues by immunohistochemistry. The RNA and protein expression patterns of *TRIM8* and *UHRF2* in the three cell lines were generally consistent, although some minor discrepancies were observed. These differences could be attributed to posttranscriptional RNA and posttranslational protein modifications. PPI network analysis indicated that TRIM8 could interact with proteins, such as p53, SMAD9, and PIAS3, which are involved in the p53 signaling pathway, TGF-beta signaling pathway, and JAK-STAT signaling pathway. While UHRF2 could not only interact with other Ub/UBL-related proteins that regulate Ub/UBL protein modification, it could also interact with PCNA, DNMT1, HDAC1, and TRDMT1, which participate in DNA replication, cell cycle progression, microRNAs in cancer, etc. (Fig. 5F).

According to the KEGG analysis, the DEGs were found to be involved mainly in the PI3K-Akt signaling pathway, calcium signaling pathway, MAPK signaling pathway, etc. Substantial evidence has indicated that the PI3K-Akt signaling pathway is frequently hyperactivated in osteosarcoma and contributes to tumorigenesis, proliferation, invasion, cell cycle progression, inhibition of apoptosis, angiogenesis, metastasis and chemoresistance [37–41]. The calcium signaling pathway was reported to affect cell viability in osteosarcoma cell lines [42, 43]. Finally, although the role of the MAPK signaling pathway in osteosarcoma is not fully understood and has even been shown to be contradictory in different studies, it is known that it can affect the angiogenesis, proliferation, migration and metastasis of osteosarcoma [44–47].

In addition, according to GSEA, the IL-17 signaling pathway, necroptosis pathway, and proteoglycans in cancer were the most highly enriched pathways, which might also be involved in the different prognoses between the high- and low-score groups. It has been reported that upregulation of the IL-17 signaling pathway is associated with metastasis and poor prognosis in patients with osteosarcoma [48–50]. Necroptosis often occurs inside overgrown tumors, which can result in inflammatory cells infiltrating into the tumor site and forming a favorable microenvironment for tumor metastasis [51–53]. However, the detailed role of necroptosis in the development and progression of osteosarcoma remains unclear [54]. In this study, necroptosis was observed to be associated with poor outcomes in patients with osteosarcoma. There is also evidence suggesting that proteoglycans play a role in regulating osteosarcoma cell proliferation, migration, and ECM structure [55–57], which is consistent with our GO clustering results. The overall effect of different kinds of proteoglycans in osteosarcoma in this study was that low expression of proteoglycans was associated with poor prognosis. PPI network analysis suggested that most of the hub proteins encoded by the DEGs were vital enzymes in the signaling pathways clustered by KEGG clustering.

In general, we found that *TRIM8* and *UHRF2* might affect the prognosis of osteosarcoma patients by influencing or cooperating with the p53 signaling pathway, TGF-beta signaling pathway, cell cycle, PI3K-Akt signaling pathway, IL-17 signaling pathway, necroptosis pathway, etc.

*TRIM8* encodes a member of the tripartite motif (*TRIM*) protein family. Because the protein has a RING-finger domain, it was suspected to be an E3 ubiquitin-protein ligase according to the NCBI Reference Sequences (RefSeq). There is also evidence that *TRIM8* is involved in the ubiquitination process. Qi Li, et al. [58] reported that *TRIM8* can target TAK1 for K63-linked polyubiquitination. Wang L et al. [59] discovered direct mutual regulation between TRIM21 and TRIM8 via Lys48 (K48)-linked ubiquitination in lung and renal cancer cells. Bo Kyung A. Seong, et al. [60] reported that *TRIM8* is an E3 ligase, that can regulate EWS/FLI protein degradation. In addition, the function of TRIM8 is not limited to ubiquitination; it can also act as an oncogene or tumor suppressor in multiple cancers. TRIM8 is involved in three pivotal cellular signaling pathways, namely, the p53 tumor suppressor, NF-κB and JAK-STAT pathways, which can affect cell proliferation, the cell cycle, DNA repair, autophagy, chemo-sensitivity, inflammation and immunity [61, 62]. In this study, high *TRIM8* expression was found to be associated with poor long-term prognosis in osteosarcoma, and the same conclusion was reached by Dachang Liu et al. [63]. Intriguingly, *TRIM8* plays an opposite role in another bone sarcoma, namely, Ewing sarcoma, but the reason for this difference still needs further investigation [60].

*UHRF2* encodes a ubiquitin-ligase that is capable of ubiquitinating PCNP (a PEST-containing nuclear protein), which is involved in cell cycle regulation, cell proliferation, etc. (NCBI Reference Sequences (RefSeq)). *UHRF2* has also been reported to affect certain phenotypes of tumor cells through DNA demethylation, the ErbB3/Ras/Raf signaling pathway, and the Wnt/β-catenin signaling pathway [64–69]. Hu et al. [70] discovered that miR-196a could promote the proliferation and migration of esophageal cancer cells through the *UHRF2/TET2* axis. Zhang Y et al. [71] also reported that *UHRF2* enhances the malignancy of hepatocellular carcinoma via PARP1-mediated autophagy. In addition, studies have shown that *UHRF2* has prognostic value in osteosarcoma [72–74]. In our study, high *UHFR2* expression was also found to be associated with poor long-term prognosis.

Monocytic lineage infiltration and metastasis, which are important components of this model, have been reported to be associated with the prognosis of osteosarcoma in many studies [75–79]. The immune cells that infiltrate osteosarcoma cells include mainly macrophages and T cells, which are closely related to tumor progression and metastasis [80–82]. In our study, monocytic lineage infiltration, dominated by monocytes, macrophages and dendritic cells [36], was associated with the survival of patients with osteosarcoma, which was consistent with the findings of previous studies. In addition, drugs that target macrophage-associated genes and pathways are currently approved for clinical use and are considered promising treatments for reducing the tumor burden and extending the survival of osteosarcoma patients [8, 83–85]. In addition, drugs that can block the metastasis of osteosarcoma are now also a focus of current research and are considered promising.

In summary, we identified two Ub/UBL genes (*TRIM8* and *UHRF2*) that were most strongly associated with the survival of osteosarcoma patients. A prognostic model was established based on the gene significance score, monocytic lineage infiltration and metastasis. The model was successfully validated in an independent external cohort, and it had good prediction accuracy and discriminatory capacity, especially for 3- and 5-year prediction. *TRIM8* and *UHRF2* might affect the prognosis of osteosarcoma patients by influencing or collaborating with several important pathways, such as the p53 signaling pathway, TGF-beta signaling pathway, and cell cycle.

Finally, several limitations of this study should be acknowledged. First, due to the low incidence rate of osteosarcoma, larger sample sizes are needed for survival analysis. The TCGA dataset only comprises 84 samples with osteosarcoma survival data. While pooling data from multiple sources could increase the sample size, differences in sequencing or chip methods across different sources may introduce significant biases to the results. Second, our study primarily relied on bioinformatics analysis, with validation of the differential expression of TRIM8 and UHRF2 in osteosarcoma conducted at the tissue and cellular levels. However, the specific mechanisms by which these indicators influence the prognosis of osteosarcoma patients remain unclear. In our future research, we will validate the signaling pathways of TRIM8 and UHRF2 that potentially influence the phenotype of osteosarcoma through gene interference.

## Conclusions

*TRIM8* and *UHRF2* are potential prognostic genes for osteosarcoma. The gene significance score of these two genes, along with metastasis and monocytic lineage infiltration, can effectively predict the 3- and 5-year survival rates of patients with osteosarcoma.

### Supplementary Information

Supplementary File 1.

Supplementary File 2.

Supplementary File 3.

Supplementary File 4.

## Data Availability

As stated in methods, all the original data were downloaded from public databases. GDC TARGET-OS and GTEX data were downloaded from UCSC Xena website (https://xenabrowser.net/hub/). GSE21275 dataset was downloaded from GEO website (https://www.ncbi.nlm.nih.gov/geo/). Ub/UBL related genes were downloaded from iUUCD website (http://iuucd.biocuckoo.org/).

## Abbreviations

BP: Biological process
CC: Cellular component
CI: Confidence interval
DCA: Decision curve analysis
DEGs: Differentially expressed genes
GEO: Gene expression omnibus database
GO: Gene ontology
iUUCD: Integrated database of regulators for ubiquitin and ubiquitin-like conjugation database
KEGG: Kyoto encyclopedia of genes and genomes
KM: Kaplan–meier
LASSO: The least absolute shrinkage and selection operator
MF: Molecular function
OS: Overall survival
PPI: Protein–protein interact
TME: Tumor microenvironment
TPM: Transcript per million
TRIM8: Tripartite motif containing 8
UHRF2: Ubiquitin like with PHD and ring finger domains 2
USP: Ubiquitin-specific protease
