# Supplementary material for: The prognostic value of ubiquitin/ubiquitin-like-related genes along with immune cell infiltration and clinicopathological features in osteosarcoma
Source: J Orthop Surg Res. 2024 Jun 15;19:356. doi: 10.1186/s13018-024-04781-1 (PMC11179372; doi:10.1186/s13018-024-04781-1)
Supplement: Supplementary file 1 [file 13018_2024_4781_MOESM1_ESM.docx]

**Table S1 Univariate and multivariate Cox analyses of ubiquitin/ubiquitin-like related genes with survival**

| Gene name | Univariate analysis | | | Multivariate analysis | | |
| --- | --- | --- | --- | --- | --- | --- |
|  | Hazard Ratio | 95%CI | *P-*Value | Hazard Ratio | 95%CI | *P-*Value |
| ANAPC5 | 1.03 | 1.01-1.05 | 0.014 | 0 | 0-0 | 0 |
| ASPSCR1 | 1.03 | 1.01-1.04 | 0 | 7.66511E+17 | 173521275633.2-3.38597464874037e+24 | 0 |
| FBXL3 | 1.04 | 1.01-1.07 | 0.014 | 0 | 0-0 | 0 |
| FBXO32 | 1.02 | 1-1.03 | 0.016 | 0 | 0-0 | 0 |
| GAN | 1.14 | 1.01-1.29 | 0.035 | 0 | 0-0 | 0 |
| HCLS1 | 0.97 | 0.95-1 | 0.04 | 5.55034E+12 | 893176.37-34490644333333786624 | 0 |
| HOXB4 | 1.09 | 1.03-1.16 | 0.005 | 1.12E+27 | 545175043061253-2.28233296685874e+39 | 0 |
| ING3 | 1.11 | 1.02-1.2 | 0.016 | 0 | 0-0 | 0 |
| INPPL1 | 1.01 | 1-1.02 | 0.008 | 0 | 0-0 | 0 |
| KIAA1875 | 1.21 | 1.04-1.4 | 0.015 | 2.21E+162 | 2.14759058478753e+124-2.26689358003596e+200 | 0 |
| KLHL13 | 1.02 | 1-1.04 | 0.021 | 2860165457 | 21850.6-374385502177478 | 0 |
| LLGL2 | 1.22 | 1.03-1.44 | 0.024 | 2.79E+78 | 1.78054726707074e+47-4.38526022088272e+109 | 0 |
| MARK3 | 1.01 | 1-1.01 | 0.048 | 92430.79 | 1394.45-6126750.69 | 0 |
| NACA | 1.01 | 1-1.01 | 0.021 | 0 | 0-0 | 0 |
| NCK1 | 1.04 | 1-1.08 | 0.039 | 4.67E+24 | 35469770368312268-6.14126460352991e+32 | 0 |
| RNF138 | 1.02 | 1-1.04 | 0.036 | 8.90282E+16 | 20825072354356.6-3.80599766278224e+20 | 0 |
| RPS27A | 1 | 44562.00 | 0.015 | 13.69 | 5.05-37.06 | 0 |
| SATB2 | 1.01 | 1-1.01 | 0.006 | 0 | 0-0 | 0 |
| SH3PXD2A | 1.01 | 1-1.01 | 0.001 | 35204891.39 | 2489242.56-497896185.93 | 0 |
| TAF5L | 1.09 | 1.01-1.18 | 0.031 | 0 | 0-0 | 0 |
| TP53BP2 | 1.03 | 1.01-1.06 | 0.009 | 6.72772E+17 | 33963534464007.9-1.33266935316312e+22 | 0 |
| TRIM21 | 0.97 | 0.94-1 | 0.049 | 0 | 0-0 | 0 |
| TRIM8 | 1.01 | 1-1.01 | 0 | 0 | 0-0 | 0 |
| UBAP2 | 1.07 | 1.02-1.13 | 0.008 | 6.73E+34 | 3.21686617520207e+24-1.40773284207468e+45 | 0 |
| UBE2E2 | 0.96 | 0.92-0.99 | 0.025 | 0 | 0-0 | 0 |
| UBE2L3 | 0.99 | 0.98-1 | 0.044 | 3042.31 | 45.27-204448.85 | 0 |
| UHRF2 | 1.04 | 1.02-1.07 | 0.001 | 3.43078E+14 | 1792299747.72-65671295753602875392 | 0 |
| VAV3 | 1.06 | 1.01-1.12 | 0.028 | 0 | 0-0 | 0 |
| WDR12 | 1.06 | 1-1.11 | 0.036 | 1.88E+24 | 170992200023086-2.05618339455048e+34 | 0 |
| WDR44 | 1.02 | 1.01-1.04 | 0.005 | 0 | 0-0 | 0 |
| KLHL25 | 1.09 | 1.03-1.15 | 0.002 | 1.20E+21 | 709413245.16-2.01928457636563e+33 | 0.001 |
| NFX1 | 1.03 | 1.01-1.06 | 0.014 | 292103717.6 | 2572.17-33172199531642.8 | 0.001 |
| PCGF6 | 1.04 | 1-1.08 | 0.033 | 1.93775E+13 | 291637.14-1.28751368433122e+21 | 0.001 |
| SORBS2 | 1.09 | 1.01-1.17 | 0.018 | 0 | 0-0 | 0.003 |
| TRIM46 | 1.14 | 1.01-1.3 | 0.035 | 0 | 0-0 | 0.003 |
| VAV1 | 0.88 | 0.79-0.99 | 0.029 | 0 | 0-0 | 0.003 |
| FBXO25 | 1.11 | 1.03-1.2 | 0.008 | 5.76E+22 | 4942601.24-6.71469344653193e+38 | 0.005 |
| RNF38 | 1.03 | 1.01-1.05 | 0.015 | 0 | 0-0.04 | 0.011 |
| KBTBD3 | 1.27 | 1.03-1.56 | 0.024 | 0 | 0-0 | 0.016 |
| BOP1 | 1.01 | 1-1.01 | 0.024 | 0 | 0-0.34 | 0.018 |
| CISH | 0.84 | 0.72-0.99 | 0.034 | 8.50E+36 | 2418184.7-2.98485453400346e+67 | 0.018 |
| ATG7 | 0.86 | 0.75-0.99 | 0.035 | 1.29E+44 | 3079193.64-5.42329877559556e+81 | 0.022 |
| BCAR1 | 0.98 | 0.96-1 | 0.035 | 1389524.14 | 4.31-447628671685.42 | 0.029 |
| TNK2 | 1.03 | 1.01-1.06 | 0.002 | 397172.07 | 3.21-49070356942.87 | 0.031 |
| RHOBTB1 | 1.03 | 1-1.05 | 0.025 | 0 | 0-0.47 | 0.039 |
| PREB | 1.01 | 1-1.01 | 0.008 | 26.43 | 1.05-664.36 | 0.047 |
| BAG1 | 1.05 | 1.01-1.08 | 0.005 | 24096594.65 | 0.07-8437687458533754 | 0.09 |
| RAD23A | 1 | 1-1.01 | 0 | 0.14 | 0.01-1.37 | 0.091 |
| MDM2 | 1 | 1-1.01 | 0.043 | 0.04 | 0-2.4 | 0.123 |
| RNF32 | 1.18 | 1.07-1.31 | 0.001 | 2.91166E+16 | 0-6.72253171825003e+37 | 0.131 |
| PPARG | 0.92 | 0.87-0.98 | 0.01 | 921417787.8 | 0-5.69389567529914e+20 | 0.136 |
| VPS18 | 0.95 | 0.91-0.99 | 0.014 | 0 | 0-110.52 | 0.143 |
| RNF139 | 1 | 1-1.01 | 0.005 | 5.74 | 0.5-65.44 | 0.16 |
| USP11 | 0.99 | 0.98-1 | 0.01 | 0.02 | 0-4.75 | 0.166 |
| SPSB2 | 0.93 | 0.86-0.99 | 0.03 | 5.29288E+12 | 0-1.63817869136277e+31 | 0.177 |
| PSTPIP1 | 0.93 | 0.87-1 | 0.045 | 0 | 0-38873.29 | 0.18 |
| WDR75 | 1.03 | 1.01-1.06 | 0.013 | 0 | 0-47.75 | 0.206 |
| UFC1 | 1.01 | 1-1.01 | 0.034 | 0.1 | 0-8.01 | 0.3 |
| FBXW4 | 1.04 | 1.02-1.07 | 0.001 | 0 | 0-230.91 | 0.308 |
| UBE2S | 1 | 1-1.01 | 0.034 | 2.72 | 0.36-20.29 | 0.329 |
| LAPTM5 | 1 | 44562.00 | 0.034 | 1.39 | 0.6-3.23 | 0.448 |
| UBA2 | 1 | 1-1.01 | 0.005 | 0.18 | 0-16.12 | 0.456 |
| NBEAL1 | 1.13 | 1-1.27 | 0.042 | 0 | 0-189069119159020 | 0.468 |
| NEBL | 1.34 | 1.13-1.6 | 0.001 | 0 | 0-9.84532464604077e+27 | 0.495 |
| FBXL5 | 0.97 | 0.95-0.99 | 0.01 | 11.65 | 0-29848.13 | 0.54 |
| TAF5 | 1.12 | 1.03-1.21 | 0.006 | 56496.05 | 0-8.7493396665312e+20 | 0.565 |
| PML | 0.95 | 0.9-1 | 0.042 | 0 | 0-37651058.89 | 0.595 |
| KIF21A | 1.02 | 1.01-1.04 | 0.01 | 26.82 | 0-15118620.67 | 0.626 |
| TRIM6 | 0.67 | 0.46-0.97 | 0.036 | 0 | 0-4.7471896283613e+67 | 0.637 |
| KLHL17 | 1.03 | 1.01-1.05 | 0.012 | 0.02 | 0-392898.71 | 0.641 |
| UBB | 1 | 44562.00 | 0.044 | 0.91 | 0.59-1.38 | 0.643 |
| HECW2 | 1.06 | 1.01-1.12 | 0.026 | 0 | 0-19022172280.99 | 0.674 |
| WDR66 | 1.14 | 1.01-1.3 | 0.042 | 43808.31 | 0-3.2269558199326e+27 | 0.691 |
| FAM63B | 1.06 | 1.03-1.09 | 0 | 0.03 | 0-915057.24 | 0.696 |
| TRIM68 | 0.85 | 0.76-0.96 | 0.006 | 0 | 0-3.91150674843797e+21 | 0.716 |
| WDR1 | 0.99 | 0.99-1 | 0.032 | 1.66 | 0.05-58.3 | 0.781 |
| NCF4 | 0.96 | 0.92-1 | 0.041 | 0.18 | 0-166824797.36 | 0.872 |
| LASP1 | 0.99 | 0.98-1 | 0.013 | 1.31 | 0.05-37.19 | 0.873 |
| TRIM62 | 0.88 | 0.79-0.99 | 0.036 | 0.01 | 0-9.4052390353491e+26 | 0.891 |
| UBE2D4 | 0.92 | 0.85-1 | 0.045 | 13.22 | 0-12520662007853914112 | 0.903 |
| MAP3K5 | 0.87 | 0.77-0.98 | 0.02 | 31.02 | 0-6.14218372312931e+25 | 0.904 |
| ZBTB40 | 1.06 | 1.01-1.11 | 0.011 | 0.35 | 0-34428282.72 | 0.91 |
| RC3H1 | 1.04 | 1-1.08 | 0.045 | 2.04 | 0-23059384.78 | 0.931 |
| CORO6 | 1.05 | 1.02-1.08 | 0.002 | 0.22 | 0-24939459422754024 | 0.939 |
| ABTB1 | 1.04 | 1-1.09 | 0.048 | 0.97 | 0-6986639976570746880 | 0.999 |
| ZFAND2B | 1.04 | 1.01-1.08 | 0.018 | 1 | 0-13213421.72 | 1 |
| AAMP | 1 | 1-1.01 | 0.322 | NA | NA | NA |
| ABI1 | 1.01 | 0.99-1.03 | 0.525 | NA | NA | NA |
| ABI2 | 1.05 | 0.99-1.12 | 0.085 | NA | NA | NA |
| ABI3 | 0.99 | 0.96-1.02 | 0.454 | NA | NA | NA |
| ABL1 | 1 | 0.99-1.01 | 0.433 | NA | NA | NA |
| ABL2 | 1 | 0.98-1.02 | 0.887 | NA | NA | NA |
| ABTB2 | 0.98 | 0.92-1.05 | 0.648 | NA | NA | NA |
| ADRM1 | 1 | 0.99-1.01 | 0.723 | NA | NA | NA |
| AHCTF1 | 1.04 | 1-1.08 | 0.079 | NA | NA | NA |
| AHI1 | 1.02 | 0.95-1.1 | 0.522 | NA | NA | NA |
| AHR | 0.99 | 0.98-1 | 0.197 | NA | NA | NA |
| AMFR | 1 | 0.98-1.02 | 0.893 | NA | NA | NA |
| AMPH | 0.98 | 0.95-1.01 | 0.236 | NA | NA | NA |
| ANAPC1 | 1.03 | 0.93-1.14 | 0.595 | NA | NA | NA |
| ANAPC10 | 0.97 | 0.91-1.04 | 0.414 | NA | NA | NA |
| ANAPC11 | 1 | 0.99-1.01 | 0.923 | NA | NA | NA |
| ANAPC13 | 1 | 0.99-1.01 | 0.556 | NA | NA | NA |
| ANAPC2 | 0.98 | 0.95-1.02 | 0.297 | NA | NA | NA |
| ANAPC4 | 1.03 | 0.96-1.11 | 0.352 | NA | NA | NA |
| ANAPC7 | 1.02 | 0.99-1.05 | 0.273 | NA | NA | NA |
| ANKRD13B | 1.03 | 1-1.06 | 0.057 | NA | NA | NA |
| ANKRD13D | 1.03 | 0.98-1.08 | 0.215 | NA | NA | NA |
| AP1G2 | 1.04 | 0.99-1.1 | 0.098 | NA | NA | NA |
| APAF1 | 1 | 0.95-1.05 | 0.99 | NA | NA | NA |
| ARHGAP10 | 0.95 | 0.88-1.03 | 0.235 | NA | NA | NA |
| ARHGAP26 | 0.88 | 0.71-1.08 | 0.209 | NA | NA | NA |
| ARHGAP4 | 0.99 | 0.96-1.02 | 0.407 | NA | NA | NA |
| ARHGEF6 | 1 | 0.99-1.01 | 0.607 | NA | NA | NA |
| ARHGEF7 | 1.01 | 0.98-1.05 | 0.397 | NA | NA | NA |
| ARIH1 | 0.98 | 0.9-1.07 | 0.663 | NA | NA | NA |
| ARIH2 | 1 | 0.97-1.03 | 0.944 | NA | NA | NA |
| ARNT | 1 | 0.99-1.01 | 0.874 | NA | NA | NA |
| ARPC1A | 1 | 0.99-1.01 | 0.555 | NA | NA | NA |
| ASB1 | 1.03 | 0.97-1.09 | 0.401 | NA | NA | NA |
| ASB11 | 0.97 | 0.81-1.15 | 0.712 | NA | NA | NA |
| ASB12 | 0.98 | 0.71-1.35 | 0.901 | NA | NA | NA |
| ASB14 | 1.08 | 0.89-1.3 | 0.444 | NA | NA | NA |
| ASB15 | 1.1 | 0.91-1.33 | 0.313 | NA | NA | NA |
| ASB4 | 0.94 | 0.74-1.19 | 0.61 | NA | NA | NA |
| ASB6 | 1 | 0.97-1.03 | 0.948 | NA | NA | NA |
| ASB7 | 1.02 | 0.95-1.1 | 0.562 | NA | NA | NA |
| ASB8 | 0.99 | 0.96-1.03 | 0.641 | NA | NA | NA |
| ASB9 | 0.96 | 0.73-1.27 | 0.773 | NA | NA | NA |
| ATG10 | 1.15 | 0.96-1.37 | 0.123 | NA | NA | NA |
| ATG12 | 0.99 | 0.92-1.07 | 0.821 | NA | NA | NA |
| ATG16L2 | 1.11 | 0.97-1.26 | 0.12 | NA | NA | NA |
| ATG3 | 1.01 | 0.99-1.03 | 0.227 | NA | NA | NA |
| ATG5 | 1.01 | 0.98-1.04 | 0.533 | NA | NA | NA |
| ATXN3 | 0.94 | 0.78-1.14 | 0.523 | NA | NA | NA |
| AUP1 | 1 | 1-1.01 | 0.251 | NA | NA | NA |
| BACH1 | 1 | 0.98-1.02 | 0.779 | NA | NA | NA |
| BACH2 | 0.99 | 0.93-1.06 | 0.731 | NA | NA | NA |
| BAP1 | 0.99 | 0.98-1.01 | 0.424 | NA | NA | NA |
| BARD1 | 1.04 | 0.98-1.1 | 0.218 | NA | NA | NA |
| BAZ1A | 1 | 0.99-1.02 | 0.568 | NA | NA | NA |
| BAZ1B | 1 | 0.99-1.01 | 0.969 | NA | NA | NA |
| BCL6 | 1.01 | 1-1.03 | 0.08 | NA | NA | NA |
| BCL6B | 1.02 | 0.99-1.05 | 0.136 | NA | NA | NA |
| BIN1 | 0.99 | 0.97-1.02 | 0.629 | NA | NA | NA |
| BIRC2 | 1 | 44562.00 | 0.185 | NA | NA | NA |
| BIRC3 | 1 | 0.95-1.05 | 0.981 | NA | NA | NA |
| BIRC6 | 0.99 | 0.97-1.01 | 0.442 | NA | NA | NA |
| BRAP | 0.99 | 0.93-1.05 | 0.638 | NA | NA | NA |
| BRCA1 | 0.99 | 0.92-1.06 | 0.688 | NA | NA | NA |
| BRSK2 | 0.91 | 0.64-1.31 | 0.613 | NA | NA | NA |
| BRWD1 | 1.05 | 0.99-1.11 | 0.126 | NA | NA | NA |
| BRWD3 | 1.02 | 0.96-1.09 | 0.485 | NA | NA | NA |
| BTBD1 | 1.01 | 1-1.01 | 0.05 | NA | NA | NA |
| BTBD11 | 1.03 | 0.95-1.12 | 0.467 | NA | NA | NA |
| BTBD2 | 1 | 0.99-1.01 | 0.79 | NA | NA | NA |
| BTBD3 | 1.01 | 0.99-1.03 | 0.355 | NA | NA | NA |
| BTBD6 | 1 | 1-1.01 | 0.684 | NA | NA | NA |
| BTBD8 | 1.19 | 0.9-1.57 | 0.226 | NA | NA | NA |
| BTBD9 | 0.95 | 0.87-1.04 | 0.296 | NA | NA | NA |
| BTK | 0.88 | 0.78-1 | 0.051 | NA | NA | NA |
| BTRC | 1.01 | 0.93-1.09 | 0.904 | NA | NA | NA |
| BUB3 | 1.01 | 1-1.03 | 0.056 | NA | NA | NA |
| BZRAP1 | 1.07 | 0.92-1.24 | 0.376 | NA | NA | NA |
| C1orf86 | 1 | 0.96-1.04 | 0.938 | NA | NA | NA |
| C6orf106 | 0.99 | 0.98-1 | 0.146 | NA | NA | NA |
| CADPS2 | 1.01 | 0.98-1.04 | 0.559 | NA | NA | NA |
| CBL | 1.03 | 1-1.07 | 0.075 | NA | NA | NA |
| CBLB | 0.98 | 0.94-1.03 | 0.429 | NA | NA | NA |
| CBLL1 | 1 | 0.96-1.04 | 0.987 | NA | NA | NA |
| CBX4 | 1 | 0.99-1.01 | 0.926 | NA | NA | NA |
| CCNB1IP1 | 1 | 0.99-1.01 | 0.829 | NA | NA | NA |
| CCNF | 0.99 | 0.95-1.03 | 0.614 | NA | NA | NA |
| CCRL2 | 0.88 | 0.72-1.09 | 0.235 | NA | NA | NA |
| CD2AP | 1.01 | 0.99-1.03 | 0.387 | NA | NA | NA |
| CDC16 | 1 | 0.99-1.01 | 0.755 | NA | NA | NA |
| CDC20 | 1 | 0.99-1 | 0.609 | NA | NA | NA |
| CDC23 | 0.99 | 0.95-1.03 | 0.576 | NA | NA | NA |
| CDC26 | 0.98 | 0.96-1.01 | 0.302 | NA | NA | NA |
| CDC27 | 0.99 | 0.97-1.01 | 0.387 | NA | NA | NA |
| CDC34 | 1 | 1-1.01 | 0.235 | NA | NA | NA |
| CDC40 | 0.97 | 0.9-1.05 | 0.508 | NA | NA | NA |
| CDCA3 | 0.98 | 0.94-1.03 | 0.526 | NA | NA | NA |
| CDRT1 | 1.15 | 0.73-1.81 | 0.55 | NA | NA | NA |
| CHAF1B | 0.99 | 0.97-1.02 | 0.638 | NA | NA | NA |
| CHD3 | 0.99 | 0.98-1 | 0.148 | NA | NA | NA |
| CHD4 | 1 | 0.99-1 | 0.405 | NA | NA | NA |
| CHD5 | 0.91 | 0.7-1.2 | 0.505 | NA | NA | NA |
| CHFR | 1.1 | 1-1.22 | 0.057 | NA | NA | NA |
| CIRH1A | 0.99 | 0.96-1.02 | 0.48 | NA | NA | NA |
| CNOT4 | 1.02 | 0.95-1.09 | 0.643 | NA | NA | NA |
| COPA | 1 | 44562.00 | 0.256 | NA | NA | NA |
| COPB2 | 1 | 0.99-1.01 | 0.735 | NA | NA | NA |
| COPS5 | 1.02 | 0.99-1.06 | 0.17 | NA | NA | NA |
| COPS6 | 1 | 0.99-1.01 | 0.945 | NA | NA | NA |
| CORO1A | 0.97 | 0.94-1.01 | 0.123 | NA | NA | NA |
| CORO1B | 0.99 | 0.97-1.01 | 0.345 | NA | NA | NA |
| CORO1C | 1 | 0.99-1.01 | 0.399 | NA | NA | NA |
| CORO2A | 1.04 | 0.98-1.1 | 0.208 | NA | NA | NA |
| CORO2B | 1.02 | 1-1.05 | 0.097 | NA | NA | NA |
| CORO7 | 0.85 | 0.69-1.05 | 0.126 | NA | NA | NA |
| CPNE1 | 1 | 0.99-1 | 0.63 | NA | NA | NA |
| CRBN | 0.93 | 0.86-1.02 | 0.116 | NA | NA | NA |
| CREBBP | 0.99 | 0.96-1.02 | 0.407 | NA | NA | NA |
| CRK | 1 | 0.99-1 | 0.478 | NA | NA | NA |
| CRKL | 1 | 0.98-1.01 | 0.709 | NA | NA | NA |
| CSTF1 | 0.99 | 0.95-1.03 | 0.556 | NA | NA | NA |
| CTTN | 1 | 0.99-1.01 | 0.676 | NA | NA | NA |
| CUEDC1 | 0.99 | 0.96-1.01 | 0.297 | NA | NA | NA |
| CUEDC2 | 1.01 | 1-1.02 | 0.088 | NA | NA | NA |
| CUL1 | 1 | 0.99-1.01 | 0.863 | NA | NA | NA |
| CUL2 | 0.99 | 0.97-1.01 | 0.399 | NA | NA | NA |
| CUL3 | 1.01 | 0.97-1.05 | 0.679 | NA | NA | NA |
| CUL4A | 1 | 0.99-1.01 | 0.887 | NA | NA | NA |
| CUL4B | 1 | 1-1.01 | 0.237 | NA | NA | NA |
| CUL5 | 1.01 | 0.99-1.03 | 0.487 | NA | NA | NA |
| CUL7 | 1 | 0.98-1.01 | 0.376 | NA | NA | NA |
| CYLD | 1.01 | 0.95-1.08 | 0.684 | NA | NA | NA |
| DCUN1D1 | 1.02 | 0.96-1.08 | 0.537 | NA | NA | NA |
| DCUN1D2 | 1 | 0.97-1.03 | 0.987 | NA | NA | NA |
| DCUN1D3 | 0.99 | 0.9-1.1 | 0.873 | NA | NA | NA |
| DCUN1D5 | 1 | 44562.00 | 0.057 | NA | NA | NA |
| DDB1 | 1 | 0.99-1.02 | 0.914 | NA | NA | NA |
| DDB2 | 0.95 | 0.9-1.01 | 0.092 | NA | NA | NA |
| DDI2 | 1.01 | 0.97-1.06 | 0.641 | NA | NA | NA |
| DDX58 | 1 | 0.99-1.01 | 0.479 | NA | NA | NA |
| DENND3 | 0.97 | 0.81-1.16 | 0.755 | NA | NA | NA |
| DHX36 | 1.01 | 0.97-1.04 | 0.765 | NA | NA | NA |
| DIDO1 | 1.01 | 0.97-1.06 | 0.619 | NA | NA | NA |
| DMWD | 1.01 | 0.98-1.03 | 0.624 | NA | NA | NA |
| DMXL1 | 0.98 | 0.87-1.11 | 0.765 | NA | NA | NA |
| DMXL2 | 1.01 | 0.92-1.11 | 0.823 | NA | NA | NA |
| DNAJB2 | 1.01 | 0.99-1.02 | 0.323 | NA | NA | NA |
| DNAJC6 | 1.03 | 0.89-1.19 | 0.733 | NA | NA | NA |
| DNMBP | 1 | 0.93-1.07 | 0.945 | NA | NA | NA |
| DPF1 | 0.96 | 0.84-1.1 | 0.543 | NA | NA | NA |
| DPF2 | 1.01 | 0.97-1.05 | 0.569 | NA | NA | NA |
| DPF3 | 0.91 | 0.71-1.18 | 0.493 | NA | NA | NA |
| DTL | 1.01 | 0.97-1.04 | 0.703 | NA | NA | NA |
| DTX1 | 0.94 | 0.71-1.23 | 0.63 | NA | NA | NA |
| DTX2 | 0.96 | 0.89-1.05 | 0.381 | NA | NA | NA |
| DTX3 | 1 | 0.99-1.01 | 0.582 | NA | NA | NA |
| DTX3L | 1 | 0.98-1.01 | 0.519 | NA | NA | NA |
| DTX4 | 1 | 1-1.01 | 0.147 | NA | NA | NA |
| DYNC1I1 | 1 | 0.95-1.06 | 0.868 | NA | NA | NA |
| DYNC1I2 | 1 | 0.99-1.01 | 0.834 | NA | NA | NA |
| DZIP3 | 0.99 | 0.94-1.04 | 0.695 | NA | NA | NA |
| E4F1 | 1.05 | 0.98-1.12 | 0.153 | NA | NA | NA |
| EED | 1.02 | 0.97-1.08 | 0.44 | NA | NA | NA |
| EML1 | 1.01 | 0.98-1.03 | 0.705 | NA | NA | NA |
| EML2 | 1.04 | 0.94-1.14 | 0.46 | NA | NA | NA |
| EML3 | 1.01 | 0.99-1.04 | 0.308 | NA | NA | NA |
| EML4 | 1.01 | 0.99-1.02 | 0.303 | NA | NA | NA |
| EML5 | 1.25 | 0.91-1.72 | 0.173 | NA | NA | NA |
| ENC1 | 1 | 0.99-1.01 | 0.398 | NA | NA | NA |
| EP300 | 1.01 | 0.98-1.03 | 0.623 | NA | NA | NA |
| EPN1 | 1 | 0.97-1.02 | 0.879 | NA | NA | NA |
| EPN2 | 1 | 0.99-1.02 | 0.622 | NA | NA | NA |
| EPN3 | 1.08 | 0.27-4.38 | 0.909 | NA | NA | NA |
| EPS15 | 1.02 | 0.99-1.05 | 0.15 | NA | NA | NA |
| EPS15L1 | 0.97 | 0.91-1.03 | 0.34 | NA | NA | NA |
| ERCC6 | 1.25 | 0.88-1.77 | 0.209 | NA | NA | NA |
| ERCC8 | 0.93 | 0.82-1.06 | 0.298 | NA | NA | NA |
| ESR1 | 0.87 | 0.52-1.45 | 0.59 | NA | NA | NA |
| FAF1 | 1 | 0.97-1.04 | 0.896 | NA | NA | NA |
| FAM53C | 1.01 | 0.96-1.06 | 0.705 | NA | NA | NA |
| FAM63A | 1 | 0.99-1.02 | 0.742 | NA | NA | NA |
| FANCD2 | 0.97 | 0.9-1.04 | 0.409 | NA | NA | NA |
| FANCL | 1 | 0.98-1.02 | 0.948 | NA | NA | NA |
| FAU | 1 | 44562.00 | 0.069 | NA | NA | NA |
| FBXL12 | 1.02 | 0.99-1.05 | 0.265 | NA | NA | NA |
| FBXL14 | 0.98 | 0.94-1.01 | 0.215 | NA | NA | NA |
| FBXL15 | 0.99 | 0.96-1.03 | 0.794 | NA | NA | NA |
| FBXL17 | 0.92 | 0.83-1.03 | 0.14 | NA | NA | NA |
| FBXL19 | 1.01 | 0.98-1.04 | 0.452 | NA | NA | NA |
| FBXL2 | 1.16 | 0.94-1.43 | 0.163 | NA | NA | NA |
| FBXL20 | 0.95 | 0.87-1.05 | 0.31 | NA | NA | NA |
| FBXL4 | 1.06 | 0.98-1.15 | 0.126 | NA | NA | NA |
| FBXL6 | 1.01 | 0.98-1.05 | 0.473 | NA | NA | NA |
| FBXL7 | 0.99 | 0.96-1.01 | 0.228 | NA | NA | NA |
| FBXL8 | 1.06 | 0.95-1.17 | 0.287 | NA | NA | NA |
| FBXO10 | 0.99 | 0.91-1.08 | 0.894 | NA | NA | NA |
| FBXO11 | 0.99 | 0.95-1.03 | 0.577 | NA | NA | NA |
| FBXO18 | 1.01 | 0.98-1.03 | 0.675 | NA | NA | NA |
| FBXO2 | 0.98 | 0.83-1.15 | 0.799 | NA | NA | NA |
| FBXO22 | 0.99 | 0.88-1.1 | 0.801 | NA | NA | NA |
| FBXO28 | 1.01 | 0.98-1.04 | 0.461 | NA | NA | NA |
| FBXO3 | 0.91 | 0.78-1.07 | 0.259 | NA | NA | NA |
| FBXO30 | 0.96 | 0.89-1.05 | 0.365 | NA | NA | NA |
| FBXO31 | 0.99 | 0.93-1.05 | 0.666 | NA | NA | NA |
| FBXO33 | 0.98 | 0.94-1.02 | 0.362 | NA | NA | NA |
| FBXO4 | 0.99 | 0.93-1.06 | 0.776 | NA | NA | NA |
| FBXO40 | 1.24 | 0.95-1.61 | 0.113 | NA | NA | NA |
| FBXO42 | 1.01 | 0.95-1.07 | 0.784 | NA | NA | NA |
| FBXO44 | 1 | 0.96-1.04 | 0.984 | NA | NA | NA |
| FBXO6 | 0.99 | 0.95-1.02 | 0.373 | NA | NA | NA |
| FBXO7 | 0.98 | 0.95-1.01 | 0.213 | NA | NA | NA |
| FBXO8 | 1.01 | 0.98-1.05 | 0.401 | NA | NA | NA |
| FBXO9 | 1.01 | 0.98-1.04 | 0.558 | NA | NA | NA |
| FBXW10 | 0.99 | 0.78-1.27 | 0.95 | NA | NA | NA |
| FBXW11 | 1 | 0.98-1.03 | 0.861 | NA | NA | NA |
| FBXW2 | 0.98 | 0.94-1.02 | 0.3 | NA | NA | NA |
| FBXW5 | 1 | 0.99-1.01 | 0.444 | NA | NA | NA |
| FBXW7 | 1 | 0.96-1.04 | 0.95 | NA | NA | NA |
| FBXW8 | 1 | 0.96-1.04 | 0.899 | NA | NA | NA |
| FBXW9 | 1.03 | 1-1.06 | 0.079 | NA | NA | NA |
| FCHSD1 | 1.02 | 0.91-1.14 | 0.799 | NA | NA | NA |
| FCHSD2 | 1 | 0.97-1.03 | 0.952 | NA | NA | NA |
| FEM1B | 0.99 | 0.97-1.01 | 0.391 | NA | NA | NA |
| FEM1C | 1.02 | 0.98-1.05 | 0.356 | NA | NA | NA |
| FGR | 1.02 | 0.87-1.2 | 0.818 | NA | NA | NA |
| FNBP1L | 0.97 | 0.95-1 | 0.075 | NA | NA | NA |
| FXN | 1.02 | 0.97-1.08 | 0.349 | NA | NA | NA |
| FYCO1 | 1.01 | 0.96-1.07 | 0.566 | NA | NA | NA |
| FYN | 1 | 0.99-1.01 | 0.601 | NA | NA | NA |
| FZR1 | 1 | 0.98-1.02 | 0.864 | NA | NA | NA |
| GABARAP | 1 | 0.99-1.01 | 0.785 | NA | NA | NA |
| GABARAPL1 | 1 | 0.97-1.02 | 0.706 | NA | NA | NA |
| GABARAPL2 | 1 | 0.99-1.01 | 0.816 | NA | NA | NA |
| GCH1 | 0.96 | 0.89-1.04 | 0.345 | NA | NA | NA |
| GEMIN5 | 1 | 0.97-1.04 | 0.906 | NA | NA | NA |
| GGA1 | 0.95 | 0.89-1.02 | 0.167 | NA | NA | NA |
| GGA2 | 1.01 | 0.98-1.05 | 0.52 | NA | NA | NA |
| GGA3 | 0.98 | 0.94-1.03 | 0.47 | NA | NA | NA |
| GNB1 | 1 | 0.99-1 | 0.484 | NA | NA | NA |
| GNB1L | 0.98 | 0.87-1.1 | 0.7 | NA | NA | NA |
| GNB2 | 1 | 0.99-1 | 0.363 | NA | NA | NA |
| GNB2L1 | 1 | 44562.00 | 0.145 | NA | NA | NA |
| GNB3 | 0.96 | 0.83-1.11 | 0.573 | NA | NA | NA |
| GNB4 | 1 | 0.99-1.01 | 0.613 | NA | NA | NA |
| GNB5 | 1 | 0.88-1.14 | 0.993 | NA | NA | NA |
| GRAP | 1.04 | 0.73-1.47 | 0.833 | NA | NA | NA |
| GRAP2 | 0.6 | 0.33-1.12 | 0.108 | NA | NA | NA |
| GRB2 | 1 | 0.99-1.01 | 0.515 | NA | NA | NA |
| GRWD1 | 0.99 | 0.96-1.03 | 0.627 | NA | NA | NA |
| GTF3C1 | 1 | 1-1.01 | 0.33 | NA | NA | NA |
| GTF3C2 | 1 | 0.97-1.02 | 0.873 | NA | NA | NA |
| HACE1 | 1.06 | 0.95-1.17 | 0.317 | NA | NA | NA |
| HCK | 0.97 | 0.94-1.01 | 0.102 | NA | NA | NA |
| HDAC4 | 1.06 | 0.98-1.13 | 0.129 | NA | NA | NA |
| HDAC6 | 1 | 0.96-1.04 | 0.874 | NA | NA | NA |
| HECTD1 | 1 | 0.99-1.02 | 0.859 | NA | NA | NA |
| HECTD2 | 1.08 | 0.99-1.18 | 0.067 | NA | NA | NA |
| HECTD3 | 0.99 | 0.97-1.02 | 0.53 | NA | NA | NA |
| HECW1 | 0.87 | 0.59-1.29 | 0.493 | NA | NA | NA |
| HERC1 | 0.94 | 0.88-1.01 | 0.071 | NA | NA | NA |
| HERC2 | 0.99 | 0.94-1.04 | 0.641 | NA | NA | NA |
| HERC3 | 1.02 | 0.94-1.12 | 0.607 | NA | NA | NA |
| HERC4 | 1.09 | 0.98-1.22 | 0.128 | NA | NA | NA |
| HERC5 | 0.97 | 0.93-1.01 | 0.192 | NA | NA | NA |
| HERC6 | 0.98 | 0.94-1.03 | 0.437 | NA | NA | NA |
| HERPUD1 | 1 | 0.99-1.01 | 0.772 | NA | NA | NA |
| HGS | 1 | 0.98-1.03 | 0.913 | NA | NA | NA |
| HIC1 | 1 | 0.95-1.04 | 0.827 | NA | NA | NA |
| HIC2 | 1 | 0.93-1.07 | 0.928 | NA | NA | NA |
| HIRA | 0.96 | 0.92-1.01 | 0.149 | NA | NA | NA |
| HUWE1 | 0.99 | 0.98-1 | 0.126 | NA | NA | NA |
| IFIH1 | 0.98 | 0.95-1.02 | 0.443 | NA | NA | NA |
| IFT122 | 1.01 | 0.93-1.1 | 0.796 | NA | NA | NA |
| IFT172 | 1.01 | 0.97-1.05 | 0.535 | NA | NA | NA |
| IFT80 | 1.03 | 0.92-1.15 | 0.631 | NA | NA | NA |
| IKBKG | 0.93 | 0.81-1.07 | 0.334 | NA | NA | NA |
| ING1 | 1.03 | 0.99-1.06 | 0.168 | NA | NA | NA |
| ING2 | 1.02 | 0.98-1.05 | 0.367 | NA | NA | NA |
| ING4 | 0.97 | 0.94-1.01 | 0.123 | NA | NA | NA |
| ING5 | 1.05 | 0.99-1.12 | 0.117 | NA | NA | NA |
| IPP | 0.99 | 0.95-1.03 | 0.541 | NA | NA | NA |
| IRF2BP1 | 1 | 0.99-1.01 | 0.78 | NA | NA | NA |
| ITCH | 1 | 0.98-1.03 | 0.684 | NA | NA | NA |
| ITSN1 | 0.95 | 0.87-1.04 | 0.288 | NA | NA | NA |
| ITSN2 | 1.06 | 0.96-1.17 | 0.225 | NA | NA | NA |
| IVNS1ABP | 1.01 | 1-1.02 | 0.091 | NA | NA | NA |
| JOSD1 | 1 | 1-1.01 | 0.356 | NA | NA | NA |
| JOSD2 | 1 | 0.98-1.01 | 0.9 | NA | NA | NA |
| KATNB1 | 1.01 | 0.95-1.06 | 0.839 | NA | NA | NA |
| KBTBD2 | 1.02 | 1-1.05 | 0.072 | NA | NA | NA |
| KBTBD4 | 0.91 | 0.81-1.02 | 0.11 | NA | NA | NA |
| KBTBD6 | 1.03 | 0.98-1.08 | 0.307 | NA | NA | NA |
| KBTBD7 | 1.03 | 0.96-1.1 | 0.39 | NA | NA | NA |
| KBTBD8 | 0.76 | 0.56-1.02 | 0.066 | NA | NA | NA |
| KCMF1 | 1 | 0.96-1.03 | 0.774 | NA | NA | NA |
| KCTD10 | 1 | 0.97-1.03 | 0.938 | NA | NA | NA |
| KCTD11 | 1.03 | 0.99-1.07 | 0.175 | NA | NA | NA |
| KCTD13 | 1.02 | 0.99-1.05 | 0.213 | NA | NA | NA |
| KCTD17 | 0.97 | 0.94-1.01 | 0.136 | NA | NA | NA |
| KCTD2 | 0.99 | 0.97-1.02 | 0.461 | NA | NA | NA |
| KCTD3 | 1.03 | 1-1.05 | 0.063 | NA | NA | NA |
| KCTD5 | 1.01 | 0.99-1.02 | 0.46 | NA | NA | NA |
| KCTD6 | 1.02 | 0.95-1.09 | 0.591 | NA | NA | NA |
| KCTD7 | 0.99 | 0.92-1.07 | 0.851 | NA | NA | NA |
| KCTD9 | 1.02 | 0.99-1.05 | 0.152 | NA | NA | NA |
| KEAP1 | 1 | 1-1.01 | 0.216 | NA | NA | NA |
| KIF21B | 0.82 | 0.63-1.05 | 0.118 | NA | NA | NA |
| KLHL10 | 1.05 | 0.74-1.51 | 0.77 | NA | NA | NA |
| KLHL11 | 1 | 0.94-1.06 | 0.989 | NA | NA | NA |
| KLHL12 | 0.98 | 0.95-1.01 | 0.276 | NA | NA | NA |
| KLHL15 | 1.03 | 0.97-1.09 | 0.376 | NA | NA | NA |
| KLHL18 | 0.96 | 0.89-1.03 | 0.274 | NA | NA | NA |
| KLHL2 | 1.01 | 1-1.02 | 0.211 | NA | NA | NA |
| KLHL20 | 1.02 | 1-1.03 | 0.113 | NA | NA | NA |
| KLHL21 | 1 | 0.99-1.01 | 0.83 | NA | NA | NA |
| KLHL22 | 0.98 | 0.95-1.02 | 0.29 | NA | NA | NA |
| KLHL23 | 1.01 | 0.96-1.06 | 0.718 | NA | NA | NA |
| KLHL24 | 1 | 0.98-1.03 | 0.673 | NA | NA | NA |
| KLHL26 | 0.95 | 0.84-1.07 | 0.379 | NA | NA | NA |
| KLHL3 | 1.15 | 0.98-1.36 | 0.087 | NA | NA | NA |
| KLHL4 | 0.99 | 0.93-1.06 | 0.751 | NA | NA | NA |
| KLHL5 | 0.98 | 0.95-1.02 | 0.276 | NA | NA | NA |
| KLHL6 | 0.77 | 0.54-1.09 | 0.141 | NA | NA | NA |
| KLHL7 | 0.94 | 0.88-1.01 | 0.099 | NA | NA | NA |
| KLHL8 | 1.01 | 0.99-1.04 | 0.314 | NA | NA | NA |
| KLHL9 | 1 | 0.98-1.02 | 0.938 | NA | NA | NA |
| LATS1 | 1 | 0.95-1.05 | 0.98 | NA | NA | NA |
| LATS2 | 1.01 | 0.99-1.02 | 0.249 | NA | NA | NA |
| LCK | 0.91 | 0.76-1.08 | 0.264 | NA | NA | NA |
| LDB2 | 1.01 | 0.96-1.06 | 0.717 | NA | NA | NA |
| LLGL1 | 1 | 0.99-1 | 0.357 | NA | NA | NA |
| LNX1 | 0.95 | 0.87-1.03 | 0.224 | NA | NA | NA |
| LONRF1 | 1.02 | 0.96-1.08 | 0.574 | NA | NA | NA |
| LONRF2 | 1.34 | 0.8-2.23 | 0.261 | NA | NA | NA |
| LONRF3 | 0.97 | 0.8-1.18 | 0.764 | NA | NA | NA |
| LRRC41 | 1 | 0.98-1.02 | 0.997 | NA | NA | NA |
| LRSAM1 | 1.01 | 0.94-1.08 | 0.883 | NA | NA | NA |
| LYN | 0.99 | 0.95-1.02 | 0.476 | NA | NA | NA |
| LYST | 0.99 | 0.88-1.11 | 0.879 | NA | NA | NA |
| LZTR1 | 0.99 | 0.96-1.02 | 0.412 | NA | NA | NA |
| MALT1 | 0.98 | 0.91-1.07 | 0.69 | NA | NA | NA |
| MAP1LC3A | 0.99 | 0.98-1.01 | 0.578 | NA | NA | NA |
| MAP1LC3B | 0.99 | 0.98-1.01 | 0.394 | NA | NA | NA |
| MAP1LC3C | 0.97 | 0.85-1.11 | 0.623 | NA | NA | NA |
| MAP2K5 | 0.95 | 0.88-1.04 | 0.267 | NA | NA | NA |
| MAP3K1 | 0.95 | 0.88-1.03 | 0.197 | NA | NA | NA |
| MAP3K10 | 0.93 | 0.86-1.01 | 0.086 | NA | NA | NA |
| MAP3K11 | 1.01 | 0.98-1.04 | 0.667 | NA | NA | NA |
| MAP3K2 | 1 | 0.97-1.03 | 0.945 | NA | NA | NA |
| MAP3K3 | 0.98 | 0.95-1 | 0.099 | NA | NA | NA |
| MAP3K9 | 0.88 | 0.67-1.15 | 0.337 | NA | NA | NA |
| MAPKBP1 | 1.02 | 0.93-1.12 | 0.673 | NA | NA | NA |
| 1-Mar | 0.85 | 0.69-1.04 | 0.109 | NA | NA | NA |
| 2-Mar | 0.97 | 0.93-1 | 0.07 | NA | NA | NA |
| 3-Mar | 1.03 | 0.95-1.11 | 0.464 | NA | NA | NA |
| 5-Mar | 1.03 | 0.99-1.06 | 0.103 | NA | NA | NA |
| 6-Mar | 1.01 | 0.99-1.03 | 0.191 | NA | NA | NA |
| 7-Mar | 1 | 0.98-1.02 | 0.898 | NA | NA | NA |
| 8-Mar | 1.02 | 0.96-1.08 | 0.569 | NA | NA | NA |
| 9-Mar | 0.99 | 0.95-1.03 | 0.475 | NA | NA | NA |
| MARK1 | 1.02 | 0.92-1.14 | 0.667 | NA | NA | NA |
| MARK2 | 1.02 | 0.99-1.04 | 0.185 | NA | NA | NA |
| MARK4 | 1 | 0.98-1.02 | 0.929 | NA | NA | NA |
| MDM4 | 1.03 | 1-1.06 | 0.076 | NA | NA | NA |
| MED8 | 1 | 0.99-1.01 | 0.95 | NA | NA | NA |
| MGRN1 | 0.96 | 0.92-1.01 | 0.091 | NA | NA | NA |
| MIB1 | 1.01 | 0.99-1.03 | 0.354 | NA | NA | NA |
| MIB2 | 1 | 0.97-1.03 | 0.934 | NA | NA | NA |
| MID1 | 0.98 | 0.95-1.01 | 0.261 | NA | NA | NA |
| MID2 | 0.95 | 0.87-1.05 | 0.332 | NA | NA | NA |
| MIDN | 1 | 0.99-1.01 | 0.709 | NA | NA | NA |
| MKRN1 | 1.01 | 0.99-1.03 | 0.543 | NA | NA | NA |
| MNAT1 | 1.02 | 0.97-1.07 | 0.36 | NA | NA | NA |
| MTF2 | 1 | 0.96-1.03 | 0.871 | NA | NA | NA |
| MYCBP2 | 1.03 | 0.99-1.06 | 0.15 | NA | NA | NA |
| MYLIP | 1 | 0.98-1.01 | 0.672 | NA | NA | NA |
| MYNN | 1.02 | 0.94-1.11 | 0.641 | NA | NA | NA |
| MYO1E | 1.01 | 1-1.02 | 0.106 | NA | NA | NA |
| MYO1F | 0.95 | 0.88-1.03 | 0.189 | NA | NA | NA |
| MYSM1 | 1.02 | 0.97-1.08 | 0.395 | NA | NA | NA |
| N4BP2 | 1 | 0.95-1.06 | 0.866 | NA | NA | NA |
| NBEA | 1.04 | 0.85-1.27 | 0.709 | NA | NA | NA |
| NBEAL2 | 1.01 | 0.9-1.13 | 0.9 | NA | NA | NA |
| NBR1 | 0.99 | 0.97-1.02 | 0.453 | NA | NA | NA |
| NCF1 | 0.84 | 0.68-1.03 | 0.096 | NA | NA | NA |
| NCF2 | 0.98 | 0.93-1.04 | 0.529 | NA | NA | NA |
| NCK2 | 1 | 0.99-1.01 | 0.429 | NA | NA | NA |
| NEB | 1 | 0.98-1.02 | 0.86 | NA | NA | NA |
| NEDD1 | 0.99 | 0.94-1.05 | 0.758 | NA | NA | NA |
| NEDD4 | 0.98 | 0.94-1.03 | 0.502 | NA | NA | NA |
| NEDD4L | 0.99 | 0.95-1.04 | 0.68 | NA | NA | NA |
| NEDD8 | 0.99 | 0.98-1 | 0.224 | NA | NA | NA |
| NEDD9 | 1 | 0.98-1.02 | 0.906 | NA | NA | NA |
| NEURL2 | 1.09 | 0.99-1.21 | 0.08 | NA | NA | NA |
| NHLRC1 | 0.98 | 0.93-1.03 | 0.454 | NA | NA | NA |
| NLE1 | 1.01 | 0.97-1.06 | 0.61 | NA | NA | NA |
| NOL10 | 1 | 0.98-1.03 | 0.906 | NA | NA | NA |
| NOSIP | 0.99 | 0.96-1.02 | 0.381 | NA | NA | NA |
| NOSTRIN | 1.06 | 0.93-1.2 | 0.41 | NA | NA | NA |
| NPHP1 | 1.15 | 0.79-1.69 | 0.465 | NA | NA | NA |
| NPLOC4 | 1 | 0.98-1.02 | 0.977 | NA | NA | NA |
| NSD1 | 1.02 | 0.98-1.06 | 0.266 | NA | NA | NA |
| NSFL1C | 0.99 | 0.96-1.02 | 0.402 | NA | NA | NA |
| NSMAF | 1 | 0.97-1.03 | 0.867 | NA | NA | NA |
| NSMCE1 | 1 | 0.97-1.02 | 0.844 | NA | NA | NA |
| NTNG2 | 0.84 | 0.62-1.14 | 0.259 | NA | NA | NA |
| NUP153 | 0.99 | 0.98-1.01 | 0.448 | NA | NA | NA |
| NUP37 | 1.01 | 0.96-1.05 | 0.775 | NA | NA | NA |
| NUP43 | 0.99 | 0.97-1.02 | 0.582 | NA | NA | NA |
| NUP62 | 0.99 | 0.98-1.01 | 0.402 | NA | NA | NA |
| NXF1 | 1.02 | 1-1.04 | 0.08 | NA | NA | NA |
| OASL | 1 | 0.99-1.01 | 0.952 | NA | NA | NA |
| OPTN | 1.01 | 1-1.03 | 0.132 | NA | NA | NA |
| OSTF1 | 1 | 0.99-1 | 0.453 | NA | NA | NA |
| OSTM1 | 0.99 | 0.97-1.01 | 0.431 | NA | NA | NA |
| OTUB1 | 1 | 0.99-1.02 | 0.709 | NA | NA | NA |
| OTUD1 | 1.01 | 0.99-1.02 | 0.53 | NA | NA | NA |
| OTUD3 | 1 | 0.94-1.06 | 0.923 | NA | NA | NA |
| OTUD4 | 1.01 | 0.96-1.05 | 0.795 | NA | NA | NA |
| OTUD5 | 1 | 0.98-1.01 | 0.549 | NA | NA | NA |
| OTUD6B | 1 | 0.95-1.06 | 0.928 | NA | NA | NA |
| PACSIN1 | 0.94 | 0.73-1.21 | 0.612 | NA | NA | NA |
| PACSIN2 | 0.99 | 0.98-1.01 | 0.435 | NA | NA | NA |
| PACSIN3 | 1 | 0.98-1.02 | 0.671 | NA | NA | NA |
| PAFAH1B1 | 1 | 0.99-1.01 | 0.862 | NA | NA | NA |
| PAK1IP1 | 1 | 0.98-1.02 | 0.724 | NA | NA | NA |
| PARD6A | 1.01 | 0.88-1.15 | 0.936 | NA | NA | NA |
| PARD6B | 1.05 | 0.95-1.16 | 0.335 | NA | NA | NA |
| PARD6G | 1.01 | 1-1.02 | 0.155 | NA | NA | NA |
| PARK2 | 1.12 | 0.83-1.51 | 0.458 | NA | NA | NA |
| PCGF1 | 0.98 | 0.94-1.03 | 0.413 | NA | NA | NA |
| PCGF3 | 1 | 0.96-1.03 | 0.825 | NA | NA | NA |
| PCGF5 | 1.01 | 0.97-1.05 | 0.546 | NA | NA | NA |
| PDLIM2 | 1.07 | 0.95-1.19 | 0.263 | NA | NA | NA |
| PDZRN3 | 0.99 | 0.97-1.01 | 0.359 | NA | NA | NA |
| PDZRN4 | 0.94 | 0.84-1.05 | 0.247 | NA | NA | NA |
| PELI1 | 1.01 | 0.99-1.03 | 0.389 | NA | NA | NA |
| PELI2 | 1.02 | 1-1.05 | 0.107 | NA | NA | NA |
| PELI3 | 1.05 | 0.95-1.16 | 0.33 | NA | NA | NA |
| PEX10 | 1 | 0.94-1.06 | 0.997 | NA | NA | NA |
| PEX12 | 1.01 | 0.94-1.09 | 0.763 | NA | NA | NA |
| PEX7 | 0.94 | 0.84-1.06 | 0.321 | NA | NA | NA |
| PHF1 | 1.01 | 0.98-1.04 | 0.488 | NA | NA | NA |
| PHF10 | 1.01 | 0.99-1.02 | 0.514 | NA | NA | NA |
| PHF14 | 1 | 0.98-1.01 | 0.689 | NA | NA | NA |
| PHF2 | 0.99 | 0.97-1.02 | 0.612 | NA | NA | NA |
| PHF21A | 1.03 | 0.98-1.1 | 0.241 | NA | NA | NA |
| PHF8 | 0.96 | 0.91-1.01 | 0.133 | NA | NA | NA |
| PHIP | 1.03 | 0.98-1.07 | 0.26 | NA | NA | NA |
| PI4K2B | 0.99 | 0.96-1.02 | 0.578 | NA | NA | NA |
| PIAS1 | 0.97 | 0.89-1.06 | 0.507 | NA | NA | NA |
| PIAS2 | 1.05 | 0.96-1.15 | 0.252 | NA | NA | NA |
| PIAS3 | 1 | 0.99-1.02 | 0.734 | NA | NA | NA |
| PIAS4 | 1 | 0.97-1.02 | 0.737 | NA | NA | NA |
| PIK3R4 | 1.01 | 0.97-1.05 | 0.599 | NA | NA | NA |
| PJA1 | 0.99 | 0.97-1.01 | 0.225 | NA | NA | NA |
| PJA2 | 1 | 0.98-1.01 | 0.758 | NA | NA | NA |
| PLAA | 1.02 | 1-1.04 | 0.053 | NA | NA | NA |
| PLCG1 | 1 | 0.98-1.02 | 0.951 | NA | NA | NA |
| PLCG2 | 0.88 | 0.74-1.05 | 0.145 | NA | NA | NA |
| PLRG1 | 0.99 | 0.97-1.01 | 0.293 | NA | NA | NA |
| POLH | 1 | 0.99-1.02 | 0.348 | NA | NA | NA |
| POLI | 1.08 | 0.95-1.23 | 0.252 | NA | NA | NA |
| POLK | 1.03 | 0.89-1.19 | 0.691 | NA | NA | NA |
| PPARA | 1.02 | 0.95-1.1 | 0.637 | NA | NA | NA |
| PPIL2 | 0.96 | 0.91-1.01 | 0.107 | NA | NA | NA |
| PPP1R13B | 1.02 | 0.99-1.04 | 0.162 | NA | NA | NA |
| PPP1R13L | 0.99 | 0.95-1.03 | 0.525 | NA | NA | NA |
| PPP2CA | 1 | 0.98-1.01 | 0.762 | NA | NA | NA |
| PPP2R2A | 1.03 | 0.99-1.06 | 0.12 | NA | NA | NA |
| PPP2R2B | 0.89 | 0.55-1.45 | 0.632 | NA | NA | NA |
| PPP2R2C | 1 | 0.62-1.6 | 0.991 | NA | NA | NA |
| PPP2R2D | 1.04 | 0.97-1.12 | 0.256 | NA | NA | NA |
| PPWD1 | 1 | 0.97-1.03 | 0.848 | NA | NA | NA |
| PRKAA1 | 1 | 0.98-1.03 | 0.871 | NA | NA | NA |
| PRKAA2 | 0.97 | 0.84-1.11 | 0.637 | NA | NA | NA |
| PRKCI | 1.02 | 0.99-1.04 | 0.134 | NA | NA | NA |
| PRPF19 | 1 | 0.99-1 | 0.543 | NA | NA | NA |
| PRPF4 | 0.99 | 0.98-1.01 | 0.363 | NA | NA | NA |
| PRPF8 | 1 | 0.99-1 | 0.433 | NA | NA | NA |
| PSEN1 | 0.97 | 0.93-1.02 | 0.244 | NA | NA | NA |
| PSEN2 | 1 | 0.97-1.04 | 0.838 | NA | NA | NA |
| PSMD14 | 1 | 0.98-1.03 | 0.927 | NA | NA | NA |
| PSMD4 | 1 | 44562.00 | 0.443 | NA | NA | NA |
| PSMD7 | 1 | 0.99-1.01 | 0.549 | NA | NA | NA |
| PWP1 | 1.01 | 0.99-1.03 | 0.348 | NA | NA | NA |
| RAB40A | 1.2 | 0.7-2.06 | 0.516 | NA | NA | NA |
| RAB40B | 0.97 | 0.88-1.07 | 0.586 | NA | NA | NA |
| RAB40C | 0.93 | 0.86-1 | 0.052 | NA | NA | NA |
| RABGEF1 | 1.01 | 0.93-1.1 | 0.799 | NA | NA | NA |
| RAD18 | 0.94 | 0.83-1.06 | 0.284 | NA | NA | NA |
| RAD23B | 1 | 0.99-1.01 | 0.88 | NA | NA | NA |
| RAE1 | 1 | 0.97-1.03 | 0.861 | NA | NA | NA |
| RAG1 | 0.92 | 0.47-1.81 | 0.816 | NA | NA | NA |
| RANBP2 | 0.99 | 0.97-1.02 | 0.684 | NA | NA | NA |
| RASD2 | 0.98 | 0.91-1.07 | 0.72 | NA | NA | NA |
| RBBP4 | 1 | 0.99-1.01 | 0.553 | NA | NA | NA |
| RBBP5 | 0.99 | 0.95-1.03 | 0.59 | NA | NA | NA |
| RBBP6 | 1.02 | 1-1.05 | 0.091 | NA | NA | NA |
| RBBP7 | 0.99 | 0.99-1 | 0.269 | NA | NA | NA |
| RBX1 | 1 | 0.99-1.01 | 0.826 | NA | NA | NA |
| RCBTB1 | 1.01 | 0.97-1.06 | 0.632 | NA | NA | NA |
| RCBTB2 | 0.98 | 0.94-1.02 | 0.418 | NA | NA | NA |
| RCHY1 | 1 | 0.94-1.06 | 0.883 | NA | NA | NA |
| RFFL | 0.99 | 0.84-1.17 | 0.873 | NA | NA | NA |
| RFWD2 | 1.02 | 1-1.04 | 0.124 | NA | NA | NA |
| RFWD3 | 1 | 0.98-1.03 | 0.676 | NA | NA | NA |
| RHBDD3 | 1.01 | 0.99-1.03 | 0.44 | NA | NA | NA |
| RHOBTB2 | 0.93 | 0.87-1 | 0.057 | NA | NA | NA |
| RHOBTB3 | 1 | 0.98-1.01 | 0.802 | NA | NA | NA |
| RICTOR | 1.02 | 0.97-1.07 | 0.38 | NA | NA | NA |
| RIMBP2 | 1.01 | 0.75-1.36 | 0.933 | NA | NA | NA |
| RNF10 | 1.01 | 0.99-1.02 | 0.403 | NA | NA | NA |
| RNF103 | 1.04 | 0.97-1.1 | 0.275 | NA | NA | NA |
| RNF11 | 1 | 1-1.01 | 0.654 | NA | NA | NA |
| RNF111 | 0.97 | 0.91-1.04 | 0.432 | NA | NA | NA |
| RNF113A | 1.01 | 0.98-1.04 | 0.689 | NA | NA | NA |
| RNF121 | 1.01 | 0.99-1.04 | 0.348 | NA | NA | NA |
| RNF122 | 1.01 | 0.98-1.04 | 0.533 | NA | NA | NA |
| RNF123 | 1.01 | 0.93-1.1 | 0.755 | NA | NA | NA |
| RNF125 | 1.07 | 0.98-1.16 | 0.149 | NA | NA | NA |
| RNF126 | 1.01 | 0.99-1.03 | 0.196 | NA | NA | NA |
| RNF128 | 1.02 | 0.99-1.05 | 0.242 | NA | NA | NA |
| RNF13 | 1 | 0.99-1.01 | 0.743 | NA | NA | NA |
| RNF130 | 0.96 | 0.92-1.02 | 0.178 | NA | NA | NA |
| RNF135 | 0.98 | 0.94-1.03 | 0.514 | NA | NA | NA |
| RNF14 | 0.96 | 0.9-1.02 | 0.187 | NA | NA | NA |
| RNF141 | 0.93 | 0.85-1.03 | 0.187 | NA | NA | NA |
| RNF146 | 0.99 | 0.95-1.02 | 0.431 | NA | NA | NA |
| RNF149 | 0.99 | 0.96-1.02 | 0.459 | NA | NA | NA |
| RNF150 | 0.98 | 0.94-1.02 | 0.402 | NA | NA | NA |
| RNF152 | 0.99 | 0.91-1.08 | 0.894 | NA | NA | NA |
| RNF157 | 1.01 | 0.96-1.05 | 0.763 | NA | NA | NA |
| RNF165 | 1.09 | 0.87-1.36 | 0.453 | NA | NA | NA |
| RNF166 | 0.98 | 0.91-1.04 | 0.472 | NA | NA | NA |
| RNF167 | 1 | 0.99-1.01 | 0.539 | NA | NA | NA |
| RNF168 | 0.99 | 0.95-1.02 | 0.471 | NA | NA | NA |
| RNF170 | 1.01 | 0.92-1.11 | 0.813 | NA | NA | NA |
| RNF175 | 0.96 | 0.85-1.07 | 0.434 | NA | NA | NA |
| RNF180 | 1.01 | 0.92-1.11 | 0.831 | NA | NA | NA |
| RNF182 | 0.98 | 0.93-1.04 | 0.517 | NA | NA | NA |
| RNF185 | 0.97 | 0.94-1 | 0.082 | NA | NA | NA |
| RNF187 | 1 | 1-1.01 | 0.279 | NA | NA | NA |
| RNF2 | 1.02 | 0.99-1.05 | 0.201 | NA | NA | NA |
| RNF20 | 0.98 | 0.96-1.01 | 0.189 | NA | NA | NA |
| RNF24 | 1 | 0.97-1.03 | 0.879 | NA | NA | NA |
| RNF25 | 0.99 | 0.97-1.02 | 0.593 | NA | NA | NA |
| RNF26 | 1 | 0.99-1.01 | 0.499 | NA | NA | NA |
| RNF31 | 0.98 | 0.93-1.04 | 0.511 | NA | NA | NA |
| RNF34 | 0.96 | 0.91-1.01 | 0.097 | NA | NA | NA |
| RNF4 | 0.98 | 0.95-1.01 | 0.157 | NA | NA | NA |
| RNF40 | 0.99 | 0.97-1.02 | 0.675 | NA | NA | NA |
| RNF41 | 1.01 | 0.98-1.05 | 0.481 | NA | NA | NA |
| RNF43 | 1.03 | 0.86-1.24 | 0.714 | NA | NA | NA |
| RNF44 | 1 | 0.98-1.03 | 0.793 | NA | NA | NA |
| RNF6 | 1.01 | 0.98-1.04 | 0.626 | NA | NA | NA |
| RNF7 | 1 | 0.99-1.01 | 0.82 | NA | NA | NA |
| RNF8 | 0.99 | 0.94-1.05 | 0.791 | NA | NA | NA |
| RSPRY1 | 1 | 0.97-1.04 | 0.83 | NA | NA | NA |
| RWDD3 | 0.96 | 0.89-1.04 | 0.328 | NA | NA | NA |
| RYBP | 1 | 0.97-1.02 | 0.803 | NA | NA | NA |
| SACS | 1 | 0.98-1.03 | 0.754 | NA | NA | NA |
| SAE1 | 1 | 0.99-1 | 0.359 | NA | NA | NA |
| SART1 | 1 | 0.99-1.01 | 0.865 | NA | NA | NA |
| SATB1 | 1 | 0.96-1.04 | 0.884 | NA | NA | NA |
| SCAP | 0.98 | 0.95-1 | 0.103 | NA | NA | NA |
| SDCBP | 0.99 | 0.99-1 | 0.062 | NA | NA | NA |
| SEH1L | 1.01 | 0.96-1.07 | 0.604 | NA | NA | NA |
| SENP1 | 1 | 0.95-1.05 | 0.883 | NA | NA | NA |
| SENP2 | 1.01 | 0.97-1.05 | 0.545 | NA | NA | NA |
| SENP3 | 1.02 | 0.99-1.05 | 0.279 | NA | NA | NA |
| SENP5 | 0.99 | 0.96-1.03 | 0.671 | NA | NA | NA |
| SENP6 | 1.02 | 0.98-1.07 | 0.298 | NA | NA | NA |
| SENP7 | 1.05 | 0.98-1.13 | 0.182 | NA | NA | NA |
| SENP8 | 0.88 | 0.64-1.22 | 0.456 | NA | NA | NA |
| SETMAR | 0.94 | 0.88-1.01 | 0.109 | NA | NA | NA |
| SF3A1 | 0.99 | 0.98-1 | 0.163 | NA | NA | NA |
| SH3D19 | 1 | 0.98-1.03 | 0.801 | NA | NA | NA |
| SH3GL1 | 1 | 0.99-1 | 0.267 | NA | NA | NA |
| SH3GL2 | 0.93 | 0.87-1 | 0.067 | NA | NA | NA |
| SH3KBP1 | 1 | 0.99-1 | 0.322 | NA | NA | NA |
| SH3PXD2B | 1 | 0.99-1.01 | 0.739 | NA | NA | NA |
| SH3RF2 | 0.98 | 0.76-1.27 | 0.899 | NA | NA | NA |
| SH3YL1 | 1.04 | 0.93-1.16 | 0.465 | NA | NA | NA |
| SHARPIN | 1 | 0.99-1.01 | 0.594 | NA | NA | NA |
| SHKBP1 | 0.98 | 0.96-1 | 0.076 | NA | NA | NA |
| SHPRH | 1.14 | 0.89-1.45 | 0.312 | NA | NA | NA |
| SIAH1 | 1 | 0.91-1.1 | 0.953 | NA | NA | NA |
| SIAH2 | 1.01 | 1-1.03 | 0.133 | NA | NA | NA |
| SKP2 | 1 | 0.98-1.01 | 0.632 | NA | NA | NA |
| SMARCAD1 | 1 | 0.97-1.03 | 0.901 | NA | NA | NA |
| SMU1 | 1.02 | 1-1.04 | 0.106 | NA | NA | NA |
| SMURF1 | 0.99 | 0.94-1.04 | 0.619 | NA | NA | NA |
| SMURF2 | 0.98 | 0.94-1.03 | 0.468 | NA | NA | NA |
| SNX9 | 1 | 0.98-1.01 | 0.618 | NA | NA | NA |
| SOCS1 | 0.98 | 0.95-1 | 0.097 | NA | NA | NA |
| SOCS2 | 1.01 | 0.98-1.04 | 0.481 | NA | NA | NA |
| SOCS3 | 1 | 44562.00 | 0.801 | NA | NA | NA |
| SOCS4 | 1 | 0.95-1.06 | 0.899 | NA | NA | NA |
| SOCS5 | 1 | 0.96-1.03 | 0.865 | NA | NA | NA |
| SOCS6 | 1.01 | 0.96-1.07 | 0.622 | NA | NA | NA |
| SORBS1 | 1 | 0.9-1.11 | 0.986 | NA | NA | NA |
| SORBS3 | 1.01 | 1-1.02 | 0.127 | NA | NA | NA |
| SPAG16 | 1 | 0.87-1.16 | 0.957 | NA | NA | NA |
| SPG20 | 1.01 | 0.99-1.02 | 0.523 | NA | NA | NA |
| SPOP | 0.99 | 0.97-1.01 | 0.481 | NA | NA | NA |
| SPSB1 | 0.99 | 0.98-1.01 | 0.523 | NA | NA | NA |
| SPSB4 | 0.99 | 0.94-1.04 | 0.714 | NA | NA | NA |
| SPTAN1 | 0.99 | 0.98-1.01 | 0.534 | NA | NA | NA |
| SQSTM1 | 1 | 0.99-1 | 0.345 | NA | NA | NA |
| SRC | 1 | 0.98-1.02 | 0.649 | NA | NA | NA |
| SRM | 1 | 44562.00 | 0.959 | NA | NA | NA |
| SSR3 | 1 | 1-1.01 | 0.106 | NA | NA | NA |
| STAC | 1.02 | 0.94-1.1 | 0.694 | NA | NA | NA |
| STAC2 | 1 | 0.99-1.02 | 0.731 | NA | NA | NA |
| STAC3 | 1 | 0.99-1 | 0.609 | NA | NA | NA |
| STAM | 0.99 | 0.95-1.03 | 0.543 | NA | NA | NA |
| STAM2 | 0.99 | 0.93-1.04 | 0.601 | NA | NA | NA |
| STAMBP | 1 | 0.95-1.07 | 0.892 | NA | NA | NA |
| STAMBPL1 | 1.01 | 0.83-1.23 | 0.914 | NA | NA | NA |
| STC1 | 1.01 | 1-1.02 | 0.173 | NA | NA | NA |
| STRAP | 1 | 0.99-1 | 0.283 | NA | NA | NA |
| STRN | 1.04 | 1-1.08 | 0.056 | NA | NA | NA |
| STRN3 | 1 | 0.99-1.02 | 0.588 | NA | NA | NA |
| STRN4 | 1 | 0.98-1.02 | 0.906 | NA | NA | NA |
| STUB1 | 1 | 0.98-1.02 | 0.854 | NA | NA | NA |
| STXBP5 | 0.97 | 0.9-1.04 | 0.332 | NA | NA | NA |
| SUMO1 | 1.01 | 1-1.01 | 0.13 | NA | NA | NA |
| SUMO2 | 1 | 1-1.01 | 0.803 | NA | NA | NA |
| SUMO3 | 1 | 1-1.01 | 0.249 | NA | NA | NA |
| SYVN1 | 1.01 | 0.99-1.03 | 0.287 | NA | NA | NA |
| TAB3 | 0.92 | 0.8-1.07 | 0.293 | NA | NA | NA |
| TAF3 | 1.01 | 0.96-1.06 | 0.731 | NA | NA | NA |
| TAX1BP1 | 0.99 | 0.97-1.01 | 0.479 | NA | NA | NA |
| TBK1 | 1 | 0.96-1.04 | 0.952 | NA | NA | NA |
| TBL1X | 1 | 0.97-1.02 | 0.834 | NA | NA | NA |
| TBL1XR1 | 1 | 0.98-1.02 | 0.833 | NA | NA | NA |
| TBL2 | 1.01 | 0.99-1.02 | 0.483 | NA | NA | NA |
| TBL3 | 0.98 | 0.92-1.03 | 0.379 | NA | NA | NA |
| TCEB1 | 1 | 0.97-1.02 | 0.76 | NA | NA | NA |
| TCEB2 | 1 | 44562.00 | 0.45 | NA | NA | NA |
| TCEB3 | 0.99 | 0.97-1.01 | 0.473 | NA | NA | NA |
| TDRD3 | 0.97 | 0.95-1 | 0.075 | NA | NA | NA |
| TEP1 | 0.98 | 0.92-1.05 | 0.566 | NA | NA | NA |
| TFG | 1 | 0.99-1.01 | 0.439 | NA | NA | NA |
| THOC3 | 1.01 | 0.96-1.05 | 0.73 | NA | NA | NA |
| TLE1 | 1 | 0.98-1.02 | 0.963 | NA | NA | NA |
| TLE2 | 1 | 0.98-1.02 | 0.812 | NA | NA | NA |
| TLE3 | 0.99 | 0.96-1.03 | 0.665 | NA | NA | NA |
| TLE4 | 1 | 0.95-1.05 | 0.875 | NA | NA | NA |
| TLE6 | 1.19 | 0.83-1.71 | 0.333 | NA | NA | NA |
| TMEM129 | 0.98 | 0.95-1 | 0.087 | NA | NA | NA |
| TMF1 | 1.01 | 0.98-1.05 | 0.408 | NA | NA | NA |
| TNFAIP1 | 0.97 | 0.94-1 | 0.093 | NA | NA | NA |
| TNFAIP3 | 1.02 | 0.98-1.07 | 0.384 | NA | NA | NA |
| TNIP1 | 1 | 0.98-1.01 | 0.807 | NA | NA | NA |
| TNIP2 | 1 | 0.98-1.03 | 0.75 | NA | NA | NA |
| TNRC6C | 1 | 0.97-1.02 | 0.929 | NA | NA | NA |
| TOLLIP | 0.98 | 0.94-1.02 | 0.282 | NA | NA | NA |
| TOM1 | 1.01 | 0.99-1.03 | 0.253 | NA | NA | NA |
| TOM1L1 | 0.96 | 0.84-1.09 | 0.501 | NA | NA | NA |
| TOM1L2 | 1 | 0.99-1.01 | 0.684 | NA | NA | NA |
| TOPORS | 1.03 | 1-1.06 | 0.068 | NA | NA | NA |
| TRAF2 | 1 | 0.96-1.05 | 0.849 | NA | NA | NA |
| TRAF3 | 1 | 0.98-1.03 | 0.813 | NA | NA | NA |
| TRAF3IP2 | 1.01 | 0.96-1.07 | 0.612 | NA | NA | NA |
| TRAF4 | 1 | 0.98-1.02 | 0.986 | NA | NA | NA |
| TRAF5 | 1.04 | 0.94-1.15 | 0.424 | NA | NA | NA |
| TRAF6 | 0.98 | 0.86-1.1 | 0.689 | NA | NA | NA |
| TRAF7 | 0.99 | 0.97-1.01 | 0.471 | NA | NA | NA |
| TRAIP | 0.96 | 0.86-1.07 | 0.462 | NA | NA | NA |
| TRIM11 | 1.04 | 0.99-1.09 | 0.124 | NA | NA | NA |
| TRIM16 | 1 | 0.97-1.03 | 0.798 | NA | NA | NA |
| TRIM17 | 1.02 | 0.99-1.05 | 0.215 | NA | NA | NA |
| TRIM2 | 0.98 | 0.94-1.01 | 0.229 | NA | NA | NA |
| TRIM22 | 0.97 | 0.93-1.01 | 0.174 | NA | NA | NA |
| TRIM23 | 0.98 | 0.91-1.06 | 0.683 | NA | NA | NA |
| TRIM24 | 1.04 | 0.98-1.1 | 0.208 | NA | NA | NA |
| TRIM25 | 1 | 0.97-1.02 | 0.886 | NA | NA | NA |
| TRIM28 | 1 | 44562.00 | 0.739 | NA | NA | NA |
| TRIM3 | 1 | 0.87-1.16 | 1 | NA | NA | NA |
| TRIM32 | 0.96 | 0.92-1.01 | 0.11 | NA | NA | NA |
| TRIM33 | 1.01 | 0.99-1.04 | 0.329 | NA | NA | NA |
| TRIM35 | 1 | 0.94-1.07 | 0.976 | NA | NA | NA |
| TRIM36 | 1.02 | 0.99-1.05 | 0.14 | NA | NA | NA |
| TRIM37 | 0.97 | 0.92-1.01 | 0.137 | NA | NA | NA |
| TRIM38 | 0.97 | 0.93-1.01 | 0.141 | NA | NA | NA |
| TRIM4 | 0.99 | 0.95-1.02 | 0.507 | NA | NA | NA |
| TRIM41 | 0.99 | 0.88-1.11 | 0.858 | NA | NA | NA |
| TRIM44 | 0.97 | 0.94-1 | 0.091 | NA | NA | NA |
| TRIM45 | 0.98 | 0.92-1.04 | 0.571 | NA | NA | NA |
| TRIM47 | 1 | 0.98-1.02 | 0.72 | NA | NA | NA |
| TRIM5 | 0.96 | 0.9-1.02 | 0.156 | NA | NA | NA |
| TRIM52 | 1.01 | 0.86-1.18 | 0.926 | NA | NA | NA |
| TRIM54 | 1 | 0.96-1.04 | 0.949 | NA | NA | NA |
| TRIM55 | 0.99 | 0.96-1.02 | 0.483 | NA | NA | NA |
| TRIM56 | 0.98 | 0.96-1 | 0.062 | NA | NA | NA |
| TRIM59 | 1.01 | 0.95-1.07 | 0.821 | NA | NA | NA |
| TRIM63 | 0.95 | 0.81-1.11 | 0.534 | NA | NA | NA |
| TRIM65 | 1.01 | 0.99-1.02 | 0.411 | NA | NA | NA |
| TRIM7 | 1.03 | 0.98-1.09 | 0.238 | NA | NA | NA |
| TRIM9 | 1.02 | 0.97-1.07 | 0.505 | NA | NA | NA |
| TRIO | 1 | 0.98-1.03 | 0.757 | NA | NA | NA |
| TRIP12 | 1.01 | 1-1.02 | 0.177 | NA | NA | NA |
| TRPC4AP | 1 | 0.99-1.01 | 0.646 | NA | NA | NA |
| TSG101 | 0.98 | 0.95-1.01 | 0.122 | NA | NA | NA |
| TSSC1 | 1 | 0.94-1.06 | 0.941 | NA | NA | NA |
| TTC3 | 1 | 0.99-1.01 | 0.711 | NA | NA | NA |
| UBA52 | 1 | 44562.00 | 0.723 | NA | NA | NA |
| UBAP1 | 1 | 1-1.01 | 0.351 | NA | NA | NA |
| UBAP2L | 1 | 0.99-1.01 | 0.517 | NA | NA | NA |
| UBC | 1 | 44562.00 | 0.993 | NA | NA | NA |
| UBE2A | 1 | 0.97-1.02 | 0.766 | NA | NA | NA |
| UBE2B | 1 | 0.97-1.03 | 0.922 | NA | NA | NA |
| UBE2C | 1 | 44562.00 | 0.848 | NA | NA | NA |
| UBE2D1 | 1.01 | 0.96-1.06 | 0.765 | NA | NA | NA |
| UBE2D2 | 1 | 0.98-1.01 | 0.806 | NA | NA | NA |
| UBE2D3 | 1 | 0.99-1.01 | 0.926 | NA | NA | NA |
| UBE2E1 | 1 | 0.98-1.01 | 0.484 | NA | NA | NA |
| UBE2E3 | 1 | 0.99-1.01 | 0.72 | NA | NA | NA |
| UBE2F | 0.97 | 0.92-1.03 | 0.329 | NA | NA | NA |
| UBE2G1 | 1 | 0.98-1.02 | 0.68 | NA | NA | NA |
| UBE2G2 | 1.01 | 0.99-1.02 | 0.237 | NA | NA | NA |
| UBE2H | 1 | 0.99-1.02 | 0.741 | NA | NA | NA |
| UBE2I | 0.97 | 0.92-1.01 | 0.127 | NA | NA | NA |
| UBE2J1 | 1 | 0.99-1.01 | 0.823 | NA | NA | NA |
| UBE2J2 | 1 | 0.98-1.02 | 0.688 | NA | NA | NA |
| UBE2L6 | 0.99 | 0.98-1 | 0.077 | NA | NA | NA |
| UBE2M | 1 | 1-1.01 | 0.64 | NA | NA | NA |
| UBE2N | 1 | 0.98-1.02 | 0.922 | NA | NA | NA |
| UBE2O | 1.02 | 1-1.04 | 0.074 | NA | NA | NA |
| UBE2Q1 | 1.01 | 1-1.02 | 0.177 | NA | NA | NA |
| UBE2Q2 | 0.99 | 0.98-1.01 | 0.438 | NA | NA | NA |
| UBE2R2 | 1.01 | 1-1.02 | 0.05 | NA | NA | NA |
| UBE2T | 1 | 0.99-1.01 | 0.682 | NA | NA | NA |
| UBE2V1 | 0.99 | 0.92-1.07 | 0.832 | NA | NA | NA |
| UBE2V2 | 1 | 0.98-1.02 | 0.935 | NA | NA | NA |
| UBE2W | 1 | 0.94-1.07 | 0.979 | NA | NA | NA |
| UBE2Z | 0.99 | 0.97-1.01 | 0.217 | NA | NA | NA |
| UBE3A | 1 | 0.98-1.01 | 0.75 | NA | NA | NA |
| UBE3B | 1.03 | 0.94-1.13 | 0.568 | NA | NA | NA |
| UBE3C | 1.01 | 0.99-1.03 | 0.588 | NA | NA | NA |
| UBE4A | 1 | 0.98-1.02 | 0.987 | NA | NA | NA |
| UBE4B | 0.99 | 0.97-1.02 | 0.733 | NA | NA | NA |
| UBL3 | 1.01 | 0.99-1.02 | 0.489 | NA | NA | NA |
| UBL4A | 0.99 | 0.98-1.01 | 0.408 | NA | NA | NA |
| UBL5 | 1 | 44562.00 | 0.987 | NA | NA | NA |
| UBL7 | 1 | 0.99-1.02 | 0.397 | NA | NA | NA |
| UBLCP1 | 1 | 0.98-1.02 | 0.699 | NA | NA | NA |
| UBOX5 | 0.96 | 0.87-1.05 | 0.371 | NA | NA | NA |
| UBQLN1 | 1 | 0.98-1.02 | 0.823 | NA | NA | NA |
| UBQLN2 | 0.99 | 0.98-1.01 | 0.317 | NA | NA | NA |
| UBQLN4 | 1 | 0.99-1.01 | 0.532 | NA | NA | NA |
| UBR1 | 0.99 | 0.91-1.07 | 0.817 | NA | NA | NA |
| UBR2 | 1 | 0.97-1.02 | 0.877 | NA | NA | NA |
| UBTD1 | 0.99 | 0.98-1.01 | 0.399 | NA | NA | NA |
| UCHL1 | 1 | 44562.00 | 0.078 | NA | NA | NA |
| UCHL3 | 1.03 | 0.99-1.08 | 0.145 | NA | NA | NA |
| UCHL5 | 1.04 | 0.99-1.09 | 0.144 | NA | NA | NA |
| UFD1L | 0.98 | 0.95-1 | 0.091 | NA | NA | NA |
| UFM1 | 1.01 | 0.99-1.02 | 0.289 | NA | NA | NA |
| UHRF1 | 0.99 | 0.97-1.01 | 0.278 | NA | NA | NA |
| USP1 | 1 | 0.99-1.01 | 0.977 | NA | NA | NA |
| USP10 | 0.99 | 0.97-1.01 | 0.271 | NA | NA | NA |
| USP12 | 1.01 | 0.97-1.04 | 0.784 | NA | NA | NA |
| USP13 | 1.01 | 0.98-1.04 | 0.562 | NA | NA | NA |
| USP14 | 0.99 | 0.98-1.01 | 0.523 | NA | NA | NA |
| USP15 | 1.08 | 0.96-1.22 | 0.202 | NA | NA | NA |
| USP16 | 1 | 0.98-1.01 | 0.766 | NA | NA | NA |
| USP18 | 0.99 | 0.97-1.01 | 0.343 | NA | NA | NA |
| USP19 | 0.98 | 0.95-1.02 | 0.361 | NA | NA | NA |
| USP2 | 1.01 | 0.98-1.04 | 0.468 | NA | NA | NA |
| USP20 | 1.01 | 0.96-1.06 | 0.668 | NA | NA | NA |
| USP21 | 1.01 | 0.99-1.04 | 0.181 | NA | NA | NA |
| USP22 | 1 | 44562.00 | 0.689 | NA | NA | NA |
| USP24 | 1 | 0.96-1.05 | 0.937 | NA | NA | NA |
| USP25 | 1.01 | 1-1.02 | 0.201 | NA | NA | NA |
| USP27X | 0.91 | 0.82-1 | 0.056 | NA | NA | NA |
| USP28 | 1.02 | 0.99-1.05 | 0.225 | NA | NA | NA |
| USP3 | 1 | 0.93-1.06 | 0.907 | NA | NA | NA |
| USP30 | 0.98 | 0.9-1.07 | 0.694 | NA | NA | NA |
| USP31 | 1.01 | 0.92-1.11 | 0.877 | NA | NA | NA |
| USP32 | 0.99 | 0.95-1.04 | 0.678 | NA | NA | NA |
| USP33 | 0.99 | 0.97-1.02 | 0.564 | NA | NA | NA |
| USP34 | 1 | 0.95-1.04 | 0.925 | NA | NA | NA |
| USP35 | 1.01 | 0.92-1.1 | 0.896 | NA | NA | NA |
| USP36 | 1.02 | 0.99-1.06 | 0.242 | NA | NA | NA |
| USP37 | 1.1 | 0.99-1.22 | 0.074 | NA | NA | NA |
| USP38 | 0.98 | 0.93-1.04 | 0.478 | NA | NA | NA |
| USP39 | 1 | 0.98-1.02 | 0.725 | NA | NA | NA |
| USP4 | 0.96 | 0.92-1 | 0.072 | NA | NA | NA |
| USP40 | 1.02 | 0.95-1.08 | 0.625 | NA | NA | NA |
| USP42 | 0.99 | 0.92-1.05 | 0.65 | NA | NA | NA |
| USP44 | 1.13 | 0.85-1.49 | 0.406 | NA | NA | NA |
| USP46 | 0.96 | 0.92-1.01 | 0.128 | NA | NA | NA |
| USP47 | 0.99 | 0.95-1.03 | 0.633 | NA | NA | NA |
| USP48 | 1.02 | 0.99-1.05 | 0.285 | NA | NA | NA |
| USP49 | 1 | 0.93-1.07 | 0.907 | NA | NA | NA |
| USP5 | 0.99 | 0.98-1 | 0.141 | NA | NA | NA |
| USP51 | 0.97 | 0.87-1.08 | 0.573 | NA | NA | NA |
| USP53 | 0.96 | 0.84-1.1 | 0.538 | NA | NA | NA |
| USP54 | 1.02 | 0.92-1.14 | 0.665 | NA | NA | NA |
| USP6 | 1.44 | 0.96-2.15 | 0.081 | NA | NA | NA |
| USP7 | 0.99 | 0.97-1.02 | 0.467 | NA | NA | NA |
| USP8 | 0.96 | 0.87-1.08 | 0.519 | NA | NA | NA |
| USP9X | 0.99 | 0.97-1.01 | 0.189 | NA | NA | NA |
| USP9Y | 0.97 | 0.88-1.06 | 0.49 | NA | NA | NA |
| USPL1 | 1.02 | 0.96-1.07 | 0.574 | NA | NA | NA |
| UTP15 | 1.01 | 0.91-1.11 | 0.893 | NA | NA | NA |
| VAV2 | 1.01 | 0.99-1.03 | 0.183 | NA | NA | NA |
| VCP | 1 | 1-1.01 | 0.313 | NA | NA | NA |
| VCPIP1 | 1 | 0.94-1.06 | 0.969 | NA | NA | NA |
| VHL | 0.99 | 0.97-1 | 0.135 | NA | NA | NA |
| VPRBP | 0.99 | 0.93-1.05 | 0.673 | NA | NA | NA |
| VPS36 | 1.02 | 0.99-1.06 | 0.131 | NA | NA | NA |
| VPS41 | 0.98 | 0.95-1.02 | 0.313 | NA | NA | NA |
| WDFY1 | 1 | 0.98-1.02 | 0.963 | NA | NA | NA |
| WDFY2 | 1.04 | 0.93-1.17 | 0.459 | NA | NA | NA |
| WDFY3 | 1.01 | 0.95-1.08 | 0.726 | NA | NA | NA |
| WDHD1 | 0.99 | 0.95-1.03 | 0.653 | NA | NA | NA |
| WDR13 | 0.99 | 0.97-1.01 | 0.443 | NA | NA | NA |
| WDR17 | 1.14 | 0.86-1.51 | 0.359 | NA | NA | NA |
| WDR18 | 1 | 0.99-1.02 | 0.702 | NA | NA | NA |
| WDR20 | 1.01 | 0.94-1.09 | 0.711 | NA | NA | NA |
| WDR24 | 0.98 | 0.93-1.04 | 0.469 | NA | NA | NA |
| WDR25 | 0.98 | 0.9-1.08 | 0.7 | NA | NA | NA |
| WDR26 | 1.01 | 0.99-1.04 | 0.346 | NA | NA | NA |
| WDR27 | 1.01 | 0.97-1.04 | 0.696 | NA | NA | NA |
| WDR3 | 1.01 | 0.97-1.06 | 0.617 | NA | NA | NA |
| WDR31 | 1 | 0.85-1.17 | 0.991 | NA | NA | NA |
| WDR33 | 1.03 | 0.96-1.1 | 0.401 | NA | NA | NA |
| WDR34 | 1 | 0.99-1.01 | 0.653 | NA | NA | NA |
| WDR36 | 0.99 | 0.94-1.04 | 0.716 | NA | NA | NA |
| WDR37 | 1.02 | 0.92-1.14 | 0.688 | NA | NA | NA |
| WDR4 | 1.01 | 0.99-1.03 | 0.295 | NA | NA | NA |
| WDR41 | 1.02 | 0.97-1.08 | 0.411 | NA | NA | NA |
| WDR43 | 1.01 | 0.99-1.02 | 0.428 | NA | NA | NA |
| WDR45 | 0.98 | 0.96-1.01 | 0.204 | NA | NA | NA |
| WDR47 | 0.99 | 0.94-1.04 | 0.636 | NA | NA | NA |
| WDR48 | 0.99 | 0.94-1.05 | 0.735 | NA | NA | NA |
| WDR5 | 1 | 0.99-1.02 | 0.655 | NA | NA | NA |
| WDR53 | 0.99 | 0.93-1.04 | 0.612 | NA | NA | NA |
| WDR54 | 1.01 | 0.99-1.03 | 0.272 | NA | NA | NA |
| WDR55 | 0.99 | 0.9-1.08 | 0.821 | NA | NA | NA |
| WDR59 | 0.99 | 0.88-1.12 | 0.867 | NA | NA | NA |
| WDR5B | 0.99 | 0.91-1.07 | 0.757 | NA | NA | NA |
| WDR6 | 0.99 | 0.98-1.01 | 0.449 | NA | NA | NA |
| WDR60 | 1.02 | 0.98-1.07 | 0.255 | NA | NA | NA |
| WDR61 | 0.98 | 0.93-1.04 | 0.531 | NA | NA | NA |
| WDR62 | 1.06 | 0.97-1.15 | 0.194 | NA | NA | NA |
| WDR7 | 0.89 | 0.76-1.05 | 0.161 | NA | NA | NA |
| WDR70 | 1 | 0.95-1.05 | 0.921 | NA | NA | NA |
| WDR74 | 1.02 | 0.99-1.05 | 0.174 | NA | NA | NA |
| WDR76 | 1 | 0.98-1.02 | 0.773 | NA | NA | NA |
| WDR77 | 1 | 0.97-1.02 | 0.731 | NA | NA | NA |
| WDR78 | 0.99 | 0.8-1.22 | 0.898 | NA | NA | NA |
| WDR81 | 0.99 | 0.96-1.02 | 0.576 | NA | NA | NA |
| WDSUB1 | 0.99 | 0.92-1.07 | 0.848 | NA | NA | NA |
| WDTC1 | 0.99 | 0.96-1.01 | 0.294 | NA | NA | NA |
| WHSC1 | 0.98 | 0.95-1.02 | 0.328 | NA | NA | NA |
| WHSC1L1 | 1.02 | 0.99-1.05 | 0.229 | NA | NA | NA |
| WIPI2 | 1 | 0.98-1.02 | 0.969 | NA | NA | NA |
| WRNIP1 | 1 | 0.98-1.02 | 0.897 | NA | NA | NA |
| WSB1 | 1.01 | 1-1.03 | 0.07 | NA | NA | NA |
| WSB2 | 1 | 0.99-1.02 | 0.672 | NA | NA | NA |
| WWP1 | 1.01 | 1-1.03 | 0.058 | NA | NA | NA |
| WWP2 | 1 | 1-1.01 | 0.508 | NA | NA | NA |
| YAF2 | 1.01 | 0.84-1.22 | 0.92 | NA | NA | NA |
| YES1 | 1 | 0.99-1.01 | 0.928 | NA | NA | NA |
| YOD1 | 1.03 | 0.93-1.13 | 0.564 | NA | NA | NA |
| ZBTB1 | 1 | 0.97-1.04 | 0.821 | NA | NA | NA |
| ZBTB10 | 1.01 | 0.98-1.05 | 0.425 | NA | NA | NA |
| ZBTB11 | 1.03 | 0.95-1.1 | 0.506 | NA | NA | NA |
| ZBTB16 | 0.96 | 0.75-1.24 | 0.761 | NA | NA | NA |
| ZBTB17 | 0.98 | 0.94-1.02 | 0.279 | NA | NA | NA |
| ZBTB2 | 1.01 | 0.97-1.06 | 0.528 | NA | NA | NA |
| ZBTB20 | 0.99 | 0.93-1.07 | 0.88 | NA | NA | NA |
| ZBTB24 | 0.96 | 0.88-1.05 | 0.397 | NA | NA | NA |
| ZBTB25 | 1.03 | 0.91-1.16 | 0.649 | NA | NA | NA |
| ZBTB3 | 0.99 | 0.89-1.11 | 0.912 | NA | NA | NA |
| ZBTB33 | 1 | 0.97-1.03 | 0.978 | NA | NA | NA |
| ZBTB34 | 1 | 0.94-1.06 | 0.968 | NA | NA | NA |
| ZBTB37 | 1.11 | 0.98-1.26 | 0.094 | NA | NA | NA |
| ZBTB39 | 1.05 | 0.97-1.13 | 0.24 | NA | NA | NA |
| ZBTB4 | 1 | 0.98-1.02 | 0.966 | NA | NA | NA |
| ZBTB41 | 1.01 | 0.95-1.07 | 0.802 | NA | NA | NA |
| ZBTB5 | 1.03 | 0.99-1.08 | 0.172 | NA | NA | NA |
| ZBTB7A | 0.99 | 0.97-1.01 | 0.409 | NA | NA | NA |
| ZBTB7B | 1.01 | 0.99-1.03 | 0.222 | NA | NA | NA |
| ZBTB9 | 0 | 0-2.31 | 0.066 | NA | NA | NA |
| ZC3H12A | 1.01 | 0.99-1.03 | 0.35 | NA | NA | NA |
| ZC3HC1 | 1.01 | 0.97-1.05 | 0.684 | NA | NA | NA |
| ZFP91 | 1 | 0.99-1.02 | 0.657 | NA | NA | NA |
| ZNRF1 | 0.99 | 0.89-1.1 | 0.882 | NA | NA | NA |
| ZNRF2 | 1.02 | 0.95-1.11 | 0.559 | NA | NA | NA |
| ZNRF3 | 1.27 | 0.22-7.41 | 0.789 | NA | NA | NA |
| ZRANB1 | 1.03 | 0.98-1.07 | 0.252 | NA | NA | NA |
| ZRANB3 | 1.02 | 0.85-1.22 | 0.828 | NA | NA | NA |
