# Supplementary material for: The prognostic value of ubiquitin/ubiquitin-like-related genes along with immune cell infiltration and clinicopathological features in osteosarcoma
Source: J Orthop Surg Res. 2024 Jun 15;19:356. doi: 10.1186/s13018-024-04781-1 (PMC11179372; doi:10.1186/s13018-024-04781-1)
Supplement: Supplementary file 2 [file 13018_2024_4781_MOESM2_ESM.docx]

**Table S2 TME estimated by MCPcounter in TARGET-OS and GTEX cohorts**

| Sample-ID | T cells | CD8 T cells | Cytotoxic lymphocytes | B lineage | NK cells | Monocytic lineage | Myeloid dendritic cells | Neutrophils | Endothelial cells | Fibroblasts |
| --- | --- | --- | --- | --- | --- | --- | --- | --- | --- | --- |
| TARGET-40-PASUUH | 0.002218393 | 0.003314546 | 0.001612602 | 0.000308137 | 5.09732E-05 | 0.002127218 | 0.000242859 | 0.000302826 | 0.000594983 | 0.059219295 |
| TARGET-40-PAUTWB | 0.000390062 | 7.55992E-05 | 0.00015202 | 0.000251465 | 7.14348E-05 | 0.006437978 | 0.000662388 | 0.000854528 | 0.003899792 | 0.363574612 |
| TARGET-40-PAKUZU | 0.000321115 | 0.000148673 | 0.000599101 | 0.00078355 | 4.53903E-05 | 0.001404269 | 0.000188163 | 0.000386494 | 0.003613342 | 0.266298045 |
| TARGET-40-0A4I0S | 0.000426398 | 0.000138909 | 0.000225202 | 0.000381044 | 8.84624E-05 | 0.011877121 | 0.000536847 | 0.000886116 | 0.001421673 | 0.61407914 |
| TARGET-40-PARJXU | 0.000281492 | 1.74385E-05 | 0.001011443 | 0.000202912 | 0.000114617 | 0.000332104 | 0.000218038 | 0.001186978 | 0.0017462 | 0.674688467 |
| TARGET-40-PAPWWC | 0.000267481 | 6.6627E-05 | 0.002469508 | 0.000198186 | 4.3023E-05 | 0.003615468 | 0.000307572 | 0.000427334 | 0.003411836 | 0.102037206 |
| TARGET-40-PAUUML | 0.000306288 | 0.000113701 | 0.006895324 | 1.99093E-05 | 6.49596E-05 | 0.000522582 | 0.000137726 | 0.000923089 | 0.002004838 | 0.184974334 |
| TARGET-40-PAMHLF | 0.000444698 | 6.30222E-05 | 0.002268528 | 0.000168861 | 0.000108246 | 0.001631858 | 0.000253655 | 0.000722477 | 0.001397644 | 0.843896933 |
| TARGET-40-PAUBIT | 0.00049353 | 0.000157201 | 0.000243991 | 0.00016882 | 5.33678E-05 | 0.003086886 | 0.00064252 | 0.000561182 | 0.00104026 | 0.349864296 |
| TARGET-40-PASFCV | 0.000311653 | 1.90871E-05 | 0.000235273 | 0.000329412 | 0.000108802 | 0.007697026 | 0.000576439 | 0.000773096 | 0.001387151 | 0.39854377 |
| TARGET-40-PAKXLD | 0.000129812 | 2.67948E-05 | 0.000510125 | 0.009020377 | 1.88664E-05 | 0.000366458 | 0.000191367 | 0.000838744 | 0.00160525 | 0.285043413 |
| TARGET-40-0A4I4O | 0.000470365 | 4.19546E-05 | 0.000487344 | 0.000230909 | 5.63397E-05 | 0.002421193 | 6.82256E-05 | 0.000763834 | 0.000786755 | 0.275697821 |
| TARGET-40-0A4I48 | 0.000550855 | 0.000107013 | 0.000233532 | 0.00012669 | 4.36971E-05 | 0.006232755 | 0.000415203 | 0.000757512 | 0.003116246 | 0.140076716 |
| TARGET-40-PASEFS | 0.000665825 | 0.000117434 | 0.00016725 | 0.00027681 | 0.000101441 | 0.000285313 | 0.000352842 | 0.000741933 | 0.00187865 | 0.046302242 |
| TARGET-40-PARKAF | 0.000133494 | 0 | 0.001039274 | 5.68098E-05 | 3.08476E-05 | 0.004279061 | 0.000124353 | 0.000448604 | 0.002015593 | 0.460950133 |
| TARGET-40-PATUXZ | 0.000368372 | 0.000155913 | 0.000233036 | 0.000205705 | 0.000316296 | 0.001231437 | 0.000388934 | 0.000580875 | 0.002185289 | 0.143742217 |
| TARGET-40-PAMHYN | 0.000232629 | 0.000113412 | 9.07749E-05 | 0.000127383 | 4.88139E-05 | 0.003747179 | 0.000157939 | 0.000487952 | 0.001433507 | 0.449418655 |
| TARGET-40-PASEBY | 0.000237095 | 3.97417E-05 | 0.000219063 | 0.000196832 | 0.000126386 | 0.004173413 | 0.000226882 | 0.000592297 | 0.002544665 | 0.309771742 |
| TARGET-40-PANMIG | 0.000256665 | 2.40015E-05 | 0.000231082 | 0.000176354 | 4.4318E-05 | 0.002052323 | 0.000260293 | 0.000658828 | 0.001227523 | 0.97292005 |
| TARGET-40-PAKFVX | 7.97335E-05 | 2.54845E-05 | 4.49568E-05 | 8.70426E-05 | 1.71531E-05 | 0.005999882 | 6.97511E-05 | 0.000336254 | 0.001613061 | 0.332845891 |
| TARGET-40-PASNZV | 0.000459829 | 0.000156699 | 0.000345983 | 0.018227387 | 0.000107211 | 0.005985783 | 0.000393897 | 0.000523136 | 0.001817337 | 0.367008844 |
| TARGET-40-0A4I4M | 0.000331456 | 0.000182327 | 0.000145724 | 0.000792417 | 5.17371E-05 | 0.005204392 | 0.000539769 | 0.000472488 | 0.003508071 | 0.081645291 |
| TARGET-40-PAMYYJ | 0.000455801 | 9.11336E-05 | 0.00019756 | 7.13613E-05 | 4.68846E-05 | 0.002738986 | 0.000211622 | 0.000609991 | 0.001845895 | 0.261742541 |
| TARGET-40-0A4I0Q | 0.000245273 | 3.82065E-05 | 0.011153675 | 0.000367126 | 5.24137E-05 | 0.001307042 | 0.000463646 | 0.000563563 | 0.001613889 | 0.740162627 |
| TARGET-40-0A4I42 | 0.000194569 | 9.56359E-06 | 0.003430085 | 7.95318E-05 | 0.000104579 | 0.002958673 | 0.000207967 | 0.001313056 | 0.002019143 | 0.42321018 |
| TARGET-40-PALECC | 0.00017584 | 9.96552E-05 | 0.00079281 | 0.000151231 | 5.55497E-05 | 0.005303896 | 8.3972E-05 | 0.000413114 | 0.001642708 | 0.318342649 |
| TARGET-40-PAMLKS | 8.29908E-05 | 6.09874E-06 | 0.000548353 | 8.02327E-05 | 4.51268E-06 | 0.000513562 | 4.657E-05 | 0.000280782 | 0.000524261 | 0.050112644 |
| TARGET-40-PANSEN | 0.000308349 | 4.83035E-05 | 0.000147232 | 0.000161593 | 0.000170401 | 0.00346393 | 0.000581684 | 0.000763317 | 0.00214684 | 0.708889277 |
| TARGET-40-PALHRL | 0.000236543 | 5.92589E-06 | 0.00036405 | 2.62178E-05 | 1.37073E-05 | 0.002773998 | 0.000133079 | 0.000242183 | 0.000774916 | 0.104161867 |
| TARGET-40-PANZHX | 0.000461679 | 0.000339486 | 0.000382157 | 0.001579666 | 8.66095E-05 | 0.005799831 | 0.000390967 | 0.000878557 | 0.001857385 | 0.214687123 |
| TARGET-40-PALKDP | 0.000103963 | 4.8218E-05 | 3.40829E-05 | 0.000522343 | 8.69628E-05 | 0.000435669 | 0.00014384 | 0.000885806 | 0.003952284 | 0.239819174 |
| TARGET-40-0A4I65 | 0.000186699 | 7.26939E-05 | 0.001383647 | 5.82078E-05 | 6.29983E-05 | 0.003649134 | 9.6157E-05 | 0.000615488 | 0.001388839 | 0.36813992 |
| TARGET-40-0A4I6O | 0.000197831 | 3.44321E-05 | 0.001843331 | 0.000174192 | 0.000105869 | 0.000805331 | 0.000864284 | 0.000651977 | 0.003178163 | 0.60711896 |
| TARGET-40-PALFYN | 0.001114384 | 0.001580606 | 0.001464359 | 0.013339277 | 0.000171681 | 0.004468119 | 0.000420952 | 0.000555209 | 0.002284621 | 0.382262108 |
| TARGET-40-PATPBS | 0.000367518 | 2.18553E-05 | 0.000182087 | 0.000367987 | 8.6549E-05 | 0.006968065 | 0.000178588 | 0.000708639 | 0.001314198 | 0.27800431 |
| TARGET-40-PAPXGT | 0.000425269 | 8.47162E-05 | 0.000242504 | 0.000101162 | 7.45517E-05 | 0.004558073 | 0.000473006 | 0.000287004 | 0.00200884 | 0.081536075 |
| TARGET-40-0A4I4E | 0.000384963 | 5.11741E-05 | 0.002099676 | 3.57889E-05 | 2.71429E-05 | 0.000544485 | 0.000131321 | 0.000578883 | 0.002616557 | 0.263104165 |
| TARGET-40-0A4HLD | 0.000426948 | 0.000419243 | 0.000326336 | 0.000124158 | 0.000778343 | 0.000840406 | 0.000157183 | 0.001132648 | 0.000880321 | 0.147679447 |
| TARGET-40-PAUYTT | 0.00020117 | 0 | 0.000126192 | 4.92309E-05 | 3.67614E-05 | 0.000263496 | 3.31563E-05 | 0.000479698 | 0.001451315 | 0.182531322 |
| TARGET-40-0A4I0W | 0.000150427 | 4.40187E-05 | 9.19224E-05 | 9.06967E-05 | 7.65E-05 | 0.001816497 | 0.000117036 | 0.000548599 | 0.001954605 | 0.341366374 |
| TARGET-40-PARFTG | 0.000698454 | 0 | 0.000119246 | 0.002077647 | 4.29556E-05 | 0.003806265 | 0.000320491 | 0.000403502 | 0.002688133 | 0.295544305 |
| TARGET-40-0A4HX8 | 0.001033827 | 0.000387504 | 0.002965554 | 6.68007E-05 | 9.70946E-05 | 0.003121377 | 0.000264504 | 0.001524011 | 0.002427615 | 0.139467228 |
| TARGET-40-PASSLM | 0.000213362 | 2.76546E-05 | 0.001466477 | 4.10743E-05 | 0.000128551 | 0.001774665 | 0.000107929 | 0.001075672 | 0.000913474 | 0.276955199 |
| TARGET-40-PATKSS | 0.000326226 | 6.05377E-06 | 0.000810224 | 8.01879E-06 | 2.76939E-05 | 0.000367781 | 0.000237344 | 0.000652034 | 0.001172332 | 0.385722918 |
| TARGET-40-PASYUK | 0.001451691 | 0.002659628 | 0.002096155 | 0.008789003 | 0.000171799 | 0.006743412 | 0.001606771 | 0.000822011 | 0.002762265 | 0.208712694 |
| TARGET-40-PAPNVD | 0.000475105 | 0.000462725 | 0.000383847 | 0.000249868 | 0.000546434 | 0.001996184 | 0.000381711 | 0.001012036 | 0.003395166 | 0.062004485 |
| TARGET-40-PATMPU | 0.000173941 | 0 | 9.98322E-05 | 5.91937E-05 | 5.3521E-05 | 0.003292231 | 0.00024907 | 0.00070161 | 0.00189025 | 0.25937576 |
| TARGET-40-PAPFLB | 0.000132321 | 1.88351E-05 | 0.001173542 | 2.42939E-05 | 2.34065E-05 | 0.00448355 | 6.60097E-05 | 0.000834885 | 0.000745024 | 0.112628766 |
| TARGET-40-PAPIJR | 0.000371295 | 5.83746E-05 | 0.000113137 | 0.00187204 | 8.31648E-05 | 0.007253081 | 0.000349454 | 0.000483088 | 0.002475978 | 0.231463087 |
| TARGET-40-PAMEKS | 0.000215776 | 3.27196E-05 | 0.000147274 | 9.51627E-05 | 0.000140896 | 0.001429859 | 0.000251387 | 0.000946712 | 0.002097854 | 1 |
| TARGET-40-PAVDTY | 0.000115423 | 0 | 3.79559E-05 | 0.00017073 | 3.50042E-05 | 0.002205211 | 0.000142737 | 0.00045216 | 0.001287667 | 0.391859097 |
| TARGET-40-PANGPE | 0.000140019 | 1.51978E-05 | 0.000135384 | 6.73749E-05 | 0.000205453 | 0.004165196 | 0.000105697 | 0.000514218 | 0.00163302 | 0.486872897 |
| TARGET-40-PAMJXS | 0.000195276 | 0.000106756 | 0.001804596 | 0.000178904 | 6.04432E-05 | 0.00649978 | 0.000152886 | 0.000647206 | 0.001666911 | 0.395921191 |
| TARGET-40-PAKZZK | 0.000423167 | 9.73021E-05 | 0.000193187 | 5.1113E-05 | 6.81547E-05 | 0.002002624 | 0.00035082 | 0.000554112 | 0.002412764 | 0.202093835 |
| TARGET-40-PALWWX | 0.000316287 | 6.80469E-05 | 0.00087183 | 0.000580401 | 4.15255E-05 | 0.002398421 | 0.000378009 | 0.000715377 | 0.001277183 | 0.493254114 |
| TARGET-40-PASKZZ | 0.000222089 | 1.68063E-05 | 0.002982595 | 0.002008403 | 0.000197019 | 0.00352582 | 0.000202252 | 0.000638986 | 0.001980185 | 0.743608714 |
| TARGET-40-0A4HXS | 0.000257342 | 6.50465E-05 | 0.007673302 | 0.001250035 | 3.13213E-05 | 0.001744992 | 0.000183005 | 0.000513868 | 0.001054365 | 0.560341852 |
| TARGET-40-PATEEM | 0.000434666 | 0.001088741 | 0.000231794 | 0.00120194 | 0.000176377 | 0.001893408 | 0.000691254 | 0.000657428 | 0.002859981 | 0.205864251 |
| TARGET-40-PAVECB | 0.000150604 | 5.63951E-06 | 5.79477E-05 | 0.000229732 | 8.05438E-05 | 0.001479307 | 0.000334119 | 0.000600019 | 0.0016139 | 0.956400034 |
| TARGET-40-PALKGN | 0.000384705 | 0.000149393 | 0.000162168 | 0.00179841 | 2.63433E-05 | 0.004343423 | 0.000318579 | 0.00036533 | 0.002027001 | 0.159171304 |
| TARGET-40-PASRNE | 0.000259002 | 6.01464E-06 | 0.00038038 | 0.000116289 | 9.0001E-05 | 0.006078254 | 0.000306506 | 0.000505339 | 0.003169425 | 0.177607747 |
| TARGET-40-PARGTM | 0.000145302 | 0 | 8.20494E-06 | 0.000116001 | 0.00011583 | 0.005261539 | 0.000225768 | 0.000742559 | 0.001421733 | 0.375805574 |
| TARGET-40-PAUVUL | 0.000184566 | 9.08304E-05 | 0.00016915 | 0.000229669 | 3.76544E-05 | 0.001546241 | 0.000116004 | 0.00032268 | 0.001291763 | 0.188934604 |
| TARGET-40-PANGRW | 0.000210182 | 0.000974494 | 0.016108996 | 0.000331004 | 3.21821E-05 | 0.003793158 | 0.000166052 | 0.000760026 | 0.001158329 | 0.250349626 |
| TARGET-40-PAVCLP | 0.000330746 | 1.95898E-05 | 9.15745E-05 | 0.000660074 | 7.25027E-05 | 0.004942479 | 0.000272509 | 0.000515258 | 0.001732303 | 0.79565482 |
| TARGET-40-PAPKWD | 0.000189829 | 4.94336E-06 | 0.000140743 | 2.08411E-05 | 2.68483E-05 | 0.001358772 | 9.01851E-05 | 0.000349234 | 0.001150787 | 0.083141898 |
| TARGET-40-PATAWV | 0.000236049 | 6.33883E-05 | 0.000170187 | 0.010181759 | 0.000157504 | 0.00157412 | 0.000175297 | 0.001292766 | 0.001193173 | 0.268176102 |
| TARGET-40-PARBGW | 0.000243328 | 1.80807E-05 | 0.000872483 | 8.99408E-05 | 4.29205E-05 | 0.001546537 | 0.00020051 | 0.000288619 | 0.002241389 | 0.34904321 |
| TARGET-40-PANXSC | 0.000542875 | 0.00033233 | 0.000207114 | 0.001110897 | 5.32577E-05 | 0.005128521 | 0.000351731 | 0.000396402 | 0.001836878 | 0.131121198 |
| TARGET-40-0A4HMC | 0.000308166 | 0.00012019 | 0.000292888 | 0.000740538 | 3.87641E-05 | 0.005852869 | 0.000382413 | 0.000828657 | 0.001372722 | 0.152905676 |
| TARGET-40-PAUXPZ | 0.000161587 | 2.97247E-05 | 5.84333E-05 | 6.31002E-05 | 0.000130113 | 0.001260707 | 7.56321E-05 | 0.000385937 | 0.001126738 | 0.279267407 |
| TARGET-40-PATJVI | 0.000532708 | 0.000509905 | 0.00064826 | 0.004828 | 0.001977243 | 0.001771401 | 0.000565468 | 0.000804306 | 0.002719005 | 0.152139798 |
| TARGET-40-PAMRHD | 0.000181934 | 0 | 9.1492E-05 | 0.000119465 | 0.000127343 | 0.005876241 | 0.000276081 | 0.000590469 | 0.003514858 | 0.215692155 |
| TARGET-40-PALZGU | 0.000454714 | 6.88776E-05 | 6.57271E-05 | 0.000156523 | 6.23313E-05 | 0.002920445 | 0.000206761 | 0.000541059 | 0.003684867 | 0.097285014 |
| TARGET-40-PANZZJ | 0.000283016 | 7.99611E-05 | 0.000699934 | 0.000150602 | 3.82802E-05 | 0.003158066 | 0.000215152 | 0.000417651 | 0.00101412 | 0.570144208 |
| TARGET-40-PATMXR | 0.000307362 | 9.35404E-06 | 0.00113098 | 5.35284E-05 | 4.16102E-05 | 0.002047653 | 0.000473954 | 0.000513089 | 0.002235334 | 0.096601417 |
| TARGET-40-PATMIF | 0.000431005 | 0.000148482 | 0.000256671 | 0.000549206 | 0.000117043 | 0.00712912 | 0.000310056 | 0.000642461 | 0.003012696 | 0.183474819 |
| TARGET-40-PANPUM | 0.000338017 | 0.00133311 | 0.007227982 | 0.000340209 | 5.09015E-05 | 0.001329657 | 0.000250219 | 0.001046031 | 0.001847514 | 0.658249139 |
| TARGET-40-PARDAX | 0.000196012 | 5.50732E-05 | 0.000119059 | 8.53485E-05 | 8.87606E-05 | 0.002172181 | 0.000217698 | 0.000646417 | 0.002207332 | 0.164038094 |
| TARGET-40-PAUTYB | 0.001467986 | 0.000159163 | 0.000549308 | 0.000216211 | 6.63932E-05 | 0.001826596 | 0.000305906 | 0.000266988 | 0.001425677 | 0.260131447 |
| TARGET-40-PAMTCM | 0.000203554 | 0.001607955 | 0.001794584 | 2.78679E-05 | 1.29131E-05 | 0.000323683 | 0.000137055 | 0.000508764 | 0.000594525 | 0.539684173 |
| TARGET-40-0A4HY5 | 0.000345029 | 5.98797E-05 | 0.003027681 | 5.52232E-05 | 4.88989E-05 | 0.001714077 | 0.000278183 | 0.001221556 | 0.001270937 | 0.332934268 |
| TARGET-40-PAVALD | 0.000228927 | 0.000347761 | 0.002938593 | 6.42201E-05 | 1.70938E-05 | 0.000123823 | 1.91795E-05 | 0.000624577 | 0.000431689 | 0.448317366 |
| TARGET-40-0A4I8U | 0.000324613 | 5.01673E-05 | 0.000639014 | 0.000141509 | 7.15399E-05 | 0.005815906 | 6.9911E-05 | 0.000455698 | 0.001227368 | 0.201048735 |
| TARGET-40-0A4I9K | 0.000205756 | 4.15363E-05 | 0.004815167 | 0.000135231 | 5.20647E-05 | 0.006195558 | 0.001390758 | 0.000619339 | 0.00106427 | 0.592242163 |
| TARGET-40-0A4I3S | 0.000317729 | 0.001596573 | 0.039382475 | 0.000352442 | 0.000156003 | 0.001771609 | 0.002070431 | 0.001296674 | 0.001980262 | 0.120569966 |
| TARGET-40-PANVJJ | 0.00038778 | 0.000132902 | 0.000265186 | 0.018246897 | 0.00013699 | 0.001868837 | 0.000643355 | 0.000682017 | 0.001190868 | 0.795372914 |
| TARGET-40-0A4I5B | 0.000647828 | 0.000338875 | 0.000733451 | 0.030754935 | 0.000121419 | 0.004497807 | 0.000630182 | 0.000929671 | 0.003664316 | 0.181758337 |
| GTEX-1117F | 0.000175991 | 2.68293E-05 | 0.000102417 | 0.000119043 | 0.000267539 | 0.000112202 | 0.000154894 | 0.000353585 | 0.001464867 | 0.003069814 |
| GTEX-111CU | 6.26604E-05 | 1.1203E-05 | 0.000151428 | 4.50277E-05 | 0.000253632 | 0.000201059 | 0.000125425 | 0.000288441 | 0.000768618 | 0.00186383 |
| GTEX-111FC | 8.10238E-05 | 6.41001E-06 | 4.25672E-05 | 4.56037E-05 | 0.000124847 | 0.000142608 | 7.12016E-05 | 0.000243348 | 0.000634167 | 0.003343233 |
| GTEX-111YS | 5.96959E-05 | 1.75132E-05 | 7.16082E-05 | 5.08839E-05 | 0.00049385 | 0.000277687 | 0.000200875 | 0.000349934 | 0.000926561 | 0.003507505 |
| GTEX-1122O | 5.31091E-05 | 5.27929E-06 | 6.59562E-05 | 5.83384E-05 | 0.000835778 | 0.000396588 | 0.000136324 | 0.000292077 | 0.00065716 | 0.003235132 |
| GTEX-113JC | 9.13423E-05 | 1.33801E-05 | 2.18104E-05 | 0.00010944 | 7.2966E-05 | 0.000119397 | 0.000102918 | 0.000287621 | 0.000954787 | 0.00455651 |
| GTEX-117YW | 6.44472E-05 | 3.41466E-06 | 2.85714E-05 | 5.21937E-05 | 0.00011869 | 6.43982E-05 | 8.04363E-05 | 0.000208736 | 0.000663128 | 0.002473661 |
| GTEX-117YX | 6.48734E-05 | 4.80206E-06 | 8.74643E-05 | 3.6895E-05 | 0.000839652 | 0.000124002 | 9.60842E-05 | 0.000343284 | 0.000920771 | 0.001882757 |
| GTEX-1192X | 0.000108856 | 5.43997E-06 | 0.000102395 | 9.77784E-05 | 0.000606552 | 0.00021845 | 0.000178811 | 0.000630248 | 0.001322741 | 0.002312753 |
| GTEX-11DXW | 6.73195E-05 | 9.28336E-06 | 2.3213E-05 | 8.3839E-05 | 4.91891E-05 | 0.000121925 | 7.83833E-05 | 0.00019995 | 0.000673352 | 0.003993679 |
| GTEX-11DXX | 4.88716E-05 | 0 | 9.77339E-05 | 1.84482E-05 | 0.000343496 | 0.000315447 | 0.000164287 | 0.000356622 | 0.000826365 | 0.004770885 |
| GTEX-11DXY | 9.21232E-05 | 2.5142E-05 | 7.56419E-05 | 7.89987E-05 | 0.000163742 | 0.000166924 | 0.00015667 | 0.000290229 | 0.000788551 | 0.002639261 |
| GTEX-11DXZ | 6.40099E-05 | 8.22205E-06 | 8.61338E-05 | 2.50762E-05 | 0.000571394 | 0.000108996 | 7.44026E-05 | 0.000295727 | 0.000889112 | 0.001484982 |
| GTEX-11DZ1 | 9.7159E-05 | 2.19513E-05 | 5.95861E-05 | 1.98777E-05 | 0.000254983 | 8.32571E-05 | 0.000235183 | 0.000905816 | 0.001081001 | 0.002498299 |
| GTEX-11EI6 | 7.90353E-05 | 1.85873E-05 | 8.80793E-05 | 7.05504E-05 | 0.000132817 | 9.99835E-05 | 0.000139138 | 0.000457301 | 0.000769196 | 0.002660049 |
| GTEX-11EM3 | 7.04737E-05 | 5.93453E-06 | 4.84404E-05 | 6.87906E-05 | 0.000216778 | 0.000212837 | 0.000157018 | 0.000265578 | 0.000727854 | 0.002653867 |
| GTEX-11EMC | 7.5397E-05 | 3.63947E-05 | 4.48354E-05 | 5.29535E-05 | 0.000412586 | 0.000224098 | 0.00013549 | 0.000376599 | 0.001000568 | 0.003734068 |
| GTEX-11EQ8 | 0.000118599 | 0 | 3.7695E-05 | 0.000184649 | 0.00047468 | 0.000118843 | 0.000424727 | 0.00047608 | 0.000732889 | 0.002532818 |
| GTEX-11EQ9 | 5.02435E-05 | 9.13814E-06 | 9.81326E-05 | 1.8921E-05 | 0.000388301 | 0.000325595 | 0.000148615 | 0.000320191 | 0.000646014 | 0.003154547 |
| GTEX-11GS4 | 4.56737E-05 | 3.13352E-06 | 3.31798E-05 | 7.54964E-05 | 5.7651E-05 | 0.000103128 | 7.86169E-05 | 0.000211948 | 0.000589863 | 0.001581735 |
| GTEX-11GSO | 5.53393E-05 | 4.98342E-06 | 2.83903E-05 | 7.60684E-05 | 0.00030013 | 9.92677E-05 | 5.85158E-05 | 0.000273714 | 0.000625873 | 0.001685773 |
| GTEX-11GSP | 7.0855E-05 | 1.9825E-05 | 3.94706E-05 | 6.79682E-05 | 5.29199E-05 | 0.000129403 | 0.000151971 | 0.000262959 | 0.00064448 | 0.0040278 |
| GTEX-11H98 | 5.62048E-05 | 3.6822E-06 | 6.53882E-05 | 4.91008E-05 | 0.000183551 | 0.000191878 | 0.000278342 | 0.00040072 | 0.001903648 | 0.005212359 |
| GTEX-11I78 | 4.69284E-05 | 7.70461E-06 | 6.10796E-05 | 4.68844E-05 | 9.6978E-05 | 0.000262176 | 9.6586E-05 | 0.000241432 | 0.000496767 | 0.001621735 |
| GTEX-11LCK | 0.000100687 | 3.93717E-06 | 0.000106113 | 7.24469E-05 | 0.000888329 | 0.000275522 | 0.00013644 | 0.000273218 | 0.000885638 | 0.002913862 |
| GTEX-11NSD | 6.21864E-05 | 3.99829E-06 | 3.14358E-05 | 3.40215E-05 | 0.000576453 | 0.000121116 | 0.000124404 | 0.000201289 | 0.000521748 | 0.001551833 |
| GTEX-11NV4 | 7.18643E-05 | 1.36211E-05 | 4.24464E-05 | 2.21844E-05 | 0.000178489 | 9.59309E-05 | 7.57166E-05 | 0.000167459 | 0.000607804 | 0.002743116 |
| GTEX-11O72 | 0.000112831 | 9.64262E-06 | 7.63813E-05 | 7.94706E-05 | 6.93539E-05 | 7.98997E-05 | 0.000189785 | 0.001009887 | 0.001108207 | 0.004961079 |
| GTEX-11OC5 | 6.29588E-05 | 7.50667E-06 | 1.68757E-05 | 2.42506E-05 | 2.89685E-05 | 0.000160023 | 0.000139745 | 0.00026109 | 0.00067331 | 0.00254812 |
| GTEX-11OF3 | 7.74419E-05 | 8.88633E-06 | 3.91068E-05 | 5.56823E-05 | 4.41918E-05 | 0.000169045 | 0.000121527 | 0.000314993 | 0.000785156 | 0.003147712 |
| GTEX-11P7K | 4.99061E-05 | 4.4575E-06 | 3.08233E-05 | 0.00015728 | 0.000398598 | 0.000218033 | 9.36163E-05 | 0.00028301 | 0.000931584 | 0.001177067 |
| GTEX-11P81 | 5.42787E-05 | 9.93745E-06 | 1.57374E-05 | 0.000295595 | 0.000124227 | 8.16237E-05 | 0.0001027 | 0.000251301 | 0.000593872 | 0.001588905 |
| GTEX-11P82 | 4.99235E-05 | 2.4064E-06 | 3.28835E-05 | 2.68003E-05 | 0.000608763 | 0.000204283 | 5.54689E-05 | 0.0002189 | 0.000522029 | 0.000830869 |
| GTEX-11PRG | 0.000120358 | 1.93989E-05 | 5.68158E-05 | 0.000192096 | 0.000201218 | 0.000178976 | 0.00016492 | 0.000239974 | 0.000777635 | 0.006755407 |
| GTEX-11TT1 | 3.03374E-05 | 1.7452E-05 | 0.00018327 | 0.000112784 | 7.55605E-05 | 9.22842E-05 | 6.8624E-05 | 0.000364952 | 0.000648277 | 0.001333846 |
| GTEX-11TTK | 0.000135734 | 7.08504E-05 | 8.06561E-05 | 4.90366E-05 | 3.73968E-05 | 0.000199778 | 0.000437348 | 0.000628868 | 0.001186353 | 0.004435666 |
| GTEX-11TUW | 3.65149E-05 | 3.57579E-05 | 2.18073E-05 | 0.000167186 | 0.000102398 | 0.00012642 | 0.000129958 | 0.000286493 | 0.000639191 | 0.002961656 |
| GTEX-11UD1 | 0.000184413 | 6.02078E-05 | 0.000433913 | 0.000118996 | 0.000818725 | 0.000196188 | 0.000431022 | 0.000407258 | 0.001245781 | 0.015128213 |
| GTEX-11VI4 | 7.22288E-05 | 1.78158E-05 | 0.000178815 | 0.000105006 | 0.000378938 | 0.000257043 | 0.000161229 | 0.000342195 | 0.000759769 | 0.002191692 |
| GTEX-11WQC | 0.000136881 | 1.63928E-05 | 6.25769E-05 | 7.78735E-05 | 3.72587E-05 | 0.000173611 | 0.000226693 | 0.000370612 | 0.000860643 | 0.009876734 |
| GTEX-11WQK | 5.27475E-05 | 2.06167E-05 | 8.16182E-05 | 0.000375326 | 9.4199E-05 | 0.000150815 | 0.000272144 | 0.000525047 | 0.000532154 | 0.002435813 |
| GTEX-11XUK | 6.7996E-05 | 4.09395E-06 | 8.75502E-05 | 0.000116655 | 0.000265947 | 0.000254121 | 0.000133457 | 0.000276773 | 0.000729517 | 0.003417425 |
| GTEX-11ZTS | 5.26146E-05 | 3.57809E-06 | 4.27525E-05 | 7.66957E-05 | 1.20002E-05 | 8.71837E-05 | 6.73269E-05 | 0.000234862 | 0.000519671 | 0.001686037 |
| GTEX-11ZTT | 6.50836E-05 | 0 | 5.88811E-05 | 7.23225E-05 | 0.000806885 | 0.000307516 | 7.40568E-05 | 0.000303136 | 0.000689546 | 0.001836 |
| GTEX-11ZUS | 4.96602E-05 | 6.31328E-06 | 3.20559E-05 | 3.41336E-05 | 6.1656E-05 | 0.000128234 | 7.83231E-05 | 0.000207041 | 0.000603883 | 0.001876325 |
| GTEX-11ZVC | 8.75404E-05 | 0.000100941 | 5.39123E-05 | 0.000184176 | 8.04338E-05 | 0.000230582 | 0.000298402 | 0.000336647 | 0.000936808 | 0.006452935 |
| GTEX-1211K | 5.66144E-05 | 3.86859E-05 | 3.75515E-05 | 7.16077E-05 | 0.000170672 | 0.000230848 | 0.000113758 | 0.000378311 | 0.000837667 | 0.003161005 |
| GTEX-12126 | 6.17775E-05 | 8.0852E-06 | 3.78118E-05 | 1.48588E-05 | 0.000140922 | 0.000102677 | 7.42825E-05 | 0.000252926 | 0.000374679 | 0.002527016 |
| GTEX-1269C | 6.68835E-05 | 7.63678E-06 | 3.12123E-05 | 1.52053E-05 | 0.000236356 | 9.44544E-05 | 0.000105471 | 0.000338045 | 0.00071057 | 0.002229238 |
| GTEX-12BJ1 | 4.19674E-05 | 0 | 4.8332E-05 | 4.25982E-05 | 0.00047575 | 0.000260985 | 0.000186474 | 0.000265427 | 0.000508521 | 0.002792933 |
| GTEX-12C56 | 6.91162E-05 | 1.25472E-05 | 3.08378E-05 | 7.90399E-05 | 0.000673823 | 0.000147433 | 0.000137106 | 0.000243713 | 0.00082972 | 0.002191414 |
| GTEX-12KS4 | 6.60838E-05 | 7.4334E-06 | 2.3643E-05 | 3.4034E-05 | 0.000225788 | 0.000104204 | 8.72965E-05 | 0.000299462 | 0.000588941 | 0.002332479 |
| GTEX-12WSA | 0.000116609 | 3.16591E-05 | 7.87547E-05 | 6.37444E-05 | 0.000197964 | 0.000132739 | 0.000180436 | 0.00048504 | 0.000934329 | 0.005874409 |
| GTEX-12WSB | 0.000149352 | 7.1836E-05 | 0.00014182 | 0.000104074 | 4.87383E-05 | 0.000179456 | 0.000257971 | 0.000649349 | 0.001173026 | 0.003400581 |
| GTEX-12WSC | 8.22198E-05 | 1.84091E-05 | 2.31545E-05 | 1.90833E-05 | 0.000104603 | 0.000162296 | 0.000113888 | 0.00029549 | 0.000834676 | 0.004260594 |
| GTEX-12WSH | 4.08387E-05 | 7.98434E-06 | 3.70448E-05 | 6.69118E-05 | 0.000294359 | 0.000105208 | 0.000155471 | 0.000342624 | 0.000474235 | 0.00257505 |
| GTEX-12WSJ | 5.14669E-05 | 2.52028E-05 | 7.7955E-05 | 8.49049E-05 | 7.32795E-05 | 0.000465764 | 0.000117347 | 0.000316795 | 0.000773734 | 0.001883135 |
| GTEX-12WSL | 7.06446E-05 | 1.83933E-05 | 8.74763E-05 | 0.000120132 | 0.000292475 | 0.000187914 | 0.000123684 | 0.000444077 | 0.000962925 | 0.003093551 |
| GTEX-12WSM | 4.6048E-05 | 3.45841E-05 | 2.2044E-05 | 3.22776E-05 | 8.41652E-05 | 0.000275788 | 9.23322E-05 | 0.000226039 | 0.000944706 | 0.004145243 |
| GTEX-12WSN | 4.91548E-05 | 2.11552E-05 | 5.02761E-05 | 2.05027E-05 | 0.0006236 | 0.000120555 | 9.56698E-05 | 0.000344231 | 0.000775887 | 0.002875041 |
| GTEX-12ZZX | 0.000144851 | 4.67642E-06 | 7.7938E-05 | 9.94712E-05 | 0.00038476 | 0.000248 | 0.000191545 | 0.000284054 | 0.000911556 | 0.006854475 |
| GTEX-12ZZZ | 0.00011216 | 1.6635E-05 | 9.17287E-05 | 4.39932E-05 | 2.39646E-05 | 0.000202233 | 0.000259221 | 0.000358639 | 0.00061048 | 0.007330257 |
| GTEX-13111 | 7.16503E-05 | 7.13628E-06 | 4.84624E-05 | 0.000241175 | 0.000200849 | 0.00020477 | 8.30043E-05 | 0.000196224 | 0.000581098 | 0.00156534 |
| GTEX-13112 | 7.21785E-05 | 1.39123E-05 | 2.96271E-05 | 6.58528E-05 | 0.000124893 | 0.000112805 | 0.000147832 | 0.00027197 | 0.000801043 | 0.002524559 |
| GTEX-1313W | 7.74305E-05 | 1.57437E-05 | 2.00222E-05 | 6.44188E-05 | 4.9962E-05 | 0.000184866 | 0.000139018 | 0.000290385 | 0.000898681 | 0.004209328 |
| GTEX-1314G | 8.06573E-05 | 2.54978E-05 | 5.51477E-05 | 9.42005E-05 | 5.02723E-05 | 0.000100642 | 0.000119044 | 0.000409783 | 0.000735581 | 0.002589235 |
| GTEX-131XE | 5.83649E-05 | 1.01662E-05 | 4.94362E-05 | 4.44819E-05 | 0.000200879 | 7.73938E-05 | 0.000137765 | 0.000465445 | 0.000915907 | 0.001540641 |
| GTEX-131XF | 6.5805E-05 | 5.31614E-06 | 0.000117631 | 2.98835E-05 | 0.001376303 | 0.000478939 | 9.63458E-05 | 0.00028405 | 0.000830811 | 0.002587503 |
| GTEX-131XH | 8.13365E-05 | 2.43081E-05 | 5.5502E-05 | 0.000111812 | 0.000296896 | 0.000214705 | 0.000167622 | 0.00032489 | 0.000979851 | 0.009021839 |
| GTEX-131XW | 6.49765E-05 | 1.49709E-05 | 9.78317E-05 | 6.13747E-05 | 0.000154806 | 0.000161636 | 0.000127351 | 0.000290576 | 0.000707027 | 0.004915934 |
| GTEX-131YS | 9.06992E-05 | 1.52444E-05 | 2.88476E-05 | 2.74829E-05 | 0.000123476 | 0.000168276 | 0.000116875 | 0.000266484 | 0.000726697 | 0.003540186 |
| GTEX-132AR | 0.000112064 | 2.26054E-05 | 0.000100526 | 0.000177299 | 0.000247956 | 0.00025441 | 0.000464631 | 0.001130987 | 0.001499292 | 0.003703004 |
| GTEX-132NY | 0.000102326 | 2.41547E-05 | 2.54266E-05 | 5.51775E-05 | 0.000129456 | 0.000188811 | 0.000147466 | 0.000325871 | 0.001004873 | 0.007062521 |
| GTEX-132Q8 | 0.000162425 | 0 | 0.000501506 | 2.54104E-05 | 0.000762259 | 0.000200559 | 0.000145715 | 0.000622673 | 0.001089367 | 0.004416502 |
| GTEX-1339X | 3.13694E-05 | 7.46385E-06 | 3.27583E-05 | 6.64484E-05 | 0.000101186 | 6.49025E-05 | 5.36006E-05 | 0.000302392 | 0.000579181 | 0.000907201 |
| GTEX-133LE | 7.91406E-05 | 2.58954E-05 | 5.45464E-05 | 1.21667E-05 | 0.000288109 | 0.000229503 | 0.000132974 | 0.000349309 | 0.000801821 | 0.001725431 |
| GTEX-1399Q | 0.000174098 | 6.6804E-06 | 7.92467E-05 | 0.000740992 | 3.26287E-05 | 0.000193961 | 0.00031771 | 0.00045601 | 0.001103926 | 0.004425639 |
| GTEX-1399R | 7.60277E-05 | 1.94673E-05 | 7.13504E-05 | 0.000191143 | 0.000741713 | 0.000118869 | 0.000124072 | 0.000362707 | 0.0008911 | 0.001983834 |
| GTEX-1399S | 0.000101064 | 2.2998E-05 | 8.26498E-05 | 9.06868E-05 | 3.83661E-05 | 0.000187472 | 8.64673E-05 | 0.000387143 | 0.000670073 | 0.002438888 |
| GTEX-1399U | 3.93972E-05 | 8.53427E-06 | 4.02263E-05 | 1.99467E-05 | 0.000573036 | 0.000134472 | 5.80106E-05 | 0.000185837 | 0.000519698 | 0.001165769 |
| GTEX-139D8 | 0.000189264 | 4.52516E-05 | 0.000353167 | 9.56561E-05 | 0.000600447 | 0.000393831 | 0.000278411 | 0.000485766 | 0.000948108 | 0.003675114 |
| GTEX-139T4 | 0.000228384 | 0.000104056 | 0.000127434 | 6.23508E-05 | 6.84512E-05 | 0.000372381 | 0.000532201 | 0.000850439 | 0.001877987 | 0.024623415 |
| GTEX-139T6 | 4.81793E-05 | 1.54819E-05 | 7.14201E-05 | 0.000120828 | 0.000326006 | 0.000211623 | 0.000105004 | 0.000188431 | 0.000510183 | 0.002344631 |
| GTEX-139T8 | 0.000200601 | 0 | 0.000124383 | 0.000133884 | 0.000204943 | 0.000151283 | 0.000317665 | 0.000626318 | 0.001526301 | 0.00323289 |
| GTEX-139TS | 7.91545E-05 | 1.12036E-05 | 3.35272E-05 | 5.26163E-05 | 3.83826E-05 | 0.000129304 | 0.000107937 | 0.000395865 | 0.000721057 | 0.002737408 |
| GTEX-139TT | 8.57413E-05 | 8.37898E-06 | 4.2043E-05 | 6.65283E-05 | 0.000358525 | 0.000164416 | 0.000110283 | 0.000275385 | 0.000766189 | 0.005879657 |
| GTEX-139TU | 7.44851E-05 | 2.04093E-05 | 3.45793E-05 | 3.04273E-05 | 0.000129669 | 0.000177903 | 0.000140759 | 0.000285825 | 0.000762353 | 0.003460431 |
| GTEX-139UC | 7.95475E-05 | 4.2038E-05 | 3.73753E-05 | 2.60186E-05 | 6.01944E-05 | 0.000185573 | 9.46201E-05 | 0.00037212 | 0.000906191 | 0.0038376 |
| GTEX-139YR | 5.96856E-05 | 3.42155E-06 | 8.35915E-05 | 0.000101713 | 0.000103908 | 6.49195E-05 | 9.35637E-05 | 0.000331172 | 0.000791565 | 0.001397636 |
| GTEX-13CF2 | 8.85812E-05 | 1.07655E-05 | 5.03494E-05 | 4.6071E-05 | 8.82519E-05 | 0.000141091 | 0.000151262 | 0.000275416 | 0.001211466 | 0.006200312 |
| GTEX-13CF3 | 3.85401E-05 | 9.90376E-06 | 6.66019E-05 | 3.73918E-05 | 2.45459E-05 | 0.000132676 | 0.000147723 | 0.000353876 | 0.000938659 | 0.002161043 |
| GTEX-13D11 | 8.45137E-05 | 1.22623E-05 | 0.00010187 | 4.07293E-05 | 0.000381535 | 0.00033531 | 0.000339708 | 0.000338556 | 0.000859998 | 0.004917597 |
| GTEX-13FH7 | 4.20722E-05 | 4.44047E-06 | 4.01784E-05 | 2.6773E-05 | 5.6322E-05 | 0.000186399 | 6.09177E-05 | 0.000279082 | 0.00089441 | 0.001659544 |
| GTEX-13FHO | 8.85932E-05 | 1.16771E-05 | 4.55324E-05 | 8.57601E-05 | 0.000122799 | 0.000157212 | 0.000248974 | 0.000451758 | 0.00111484 | 0.002827507 |
| GTEX-13FHP | 7.49144E-05 | 1.27455E-05 | 3.00837E-05 | 0.000136907 | 6.82687E-05 | 5.21067E-05 | 0.000136139 | 0.000272658 | 0.000712901 | 0.001803222 |
| GTEX-13FTW | 5.73927E-05 | 0 | 0.000102422 | 1.75376E-05 | 0.000629814 | 0.000267816 | 0.000164286 | 0.000252603 | 0.000711516 | 0.002425184 |
| GTEX-13FTX | 6.26473E-05 | 1.03514E-05 | 0.000226326 | 6.73614E-05 | 0.00040534 | 0.000134954 | 0.000181253 | 0.000645179 | 0.000951528 | 0.001893995 |
| GTEX-13FTY | 0.000138053 | 2.9439E-05 | 6.63147E-05 | 1.98722E-05 | 7.14533E-05 | 0.000163609 | 0.000319022 | 0.000342915 | 0.001036539 | 0.022787081 |
| GTEX-13FTZ | 7.29349E-05 | 0 | 2.14381E-05 | 4.97286E-05 | 0.000108666 | 0.000181551 | 0.000107838 | 0.000349251 | 0.001043077 | 0.005765819 |
| GTEX-13FXS | 0.000116271 | 2.34668E-05 | 5.22515E-05 | 1.23852E-05 | 0.000163549 | 0.00016911 | 0.000128872 | 0.000291976 | 0.000538925 | 0.009007116 |
| GTEX-13G51 | 8.75873E-05 | 2.21753E-05 | 3.83662E-05 | 1.44847E-05 | 2.87966E-05 | 0.000128476 | 0.000110393 | 0.000209211 | 0.000762347 | 0.004910277 |
| GTEX-13IVO | 0.000219793 | 7.20749E-05 | 0.000145838 | 4.51384E-05 | 0.000221752 | 0.000420717 | 0.000337155 | 0.00080554 | 0.001663816 | 0.006310307 |
| GTEX-13JUV | 4.37224E-05 | 1.14916E-05 | 3.11123E-05 | 2.92052E-05 | 0.000148743 | 8.16883E-05 | 0.000219389 | 0.000382067 | 0.000840593 | 0.002612248 |
| GTEX-13JVG | 0.000126483 | 2.3894E-05 | 0.000139667 | 7.67042E-05 | 0.000207638 | 0.00015197 | 0.00017933 | 0.000626234 | 0.001605747 | 0.003445775 |
| GTEX-13N11 | 5.95271E-05 | 2.1558E-05 | 5.91354E-05 | 4.31407E-05 | 0.000380321 | 0.000106021 | 9.66737E-05 | 0.000265808 | 0.000901052 | 0.001431165 |
| GTEX-13N1W | 6.64764E-05 | 3.8794E-06 | 3.13574E-05 | 1.87077E-05 | 0.000314534 | 0.000140124 | 7.87507E-05 | 0.000356484 | 0.000872706 | 0.004196188 |
| GTEX-13N2G | 7.22243E-05 | 2.13587E-05 | 5.52824E-05 | 5.62663E-05 | 0.000570953 | 0.000180562 | 0.000172517 | 0.000277035 | 0.000798721 | 0.002320187 |
| GTEX-13NYB | 0.000133457 | 1.69642E-05 | 6.04425E-05 | 8.92016E-05 | 0.000174666 | 0.000237879 | 0.000144134 | 0.000401648 | 0.000964627 | 0.004581105 |
| GTEX-13NYS | 0.000172557 | 9.35656E-06 | 0.000128948 | 0.000726752 | 0.000146126 | 0.000103308 | 0.000384205 | 0.000962591 | 0.001760789 | 0.005226317 |
| GTEX-13NZ8 | 0.000128442 | 4.33209E-06 | 6.65918E-05 | 4.01783E-05 | 0.000235861 | 0.000169915 | 0.000348331 | 0.000321948 | 0.000932374 | 0.003534686 |
| GTEX-13NZ9 | 9.08688E-05 | 7.81519E-06 | 4.87836E-05 | 2.84488E-05 | 1.72059E-05 | 9.88628E-05 | 0.000182357 | 0.000353827 | 0.000608462 | 0.00355297 |
| GTEX-13NZA | 5.72222E-05 | 1.19909E-05 | 2.54017E-05 | 1.60456E-05 | 0.000230957 | 0.000166276 | 0.000100468 | 0.000324683 | 0.000648263 | 0.002801055 |
| GTEX-13NZB | 6.27294E-05 | 3.87239E-06 | 1.55859E-05 | 1.81048E-05 | 0.000215461 | 0.000147875 | 0.000105431 | 0.000315865 | 0.000534553 | 0.001756306 |
| GTEX-13O1R | 2.82157E-05 | 1.09055E-05 | 4.16611E-05 | 1.80772E-05 | 2.53004E-05 | 9.28335E-05 | 0.000136322 | 0.000388998 | 0.000661838 | 0.001622425 |
| GTEX-13O21 | 6.74574E-05 | 1.53898E-05 | 8.86389E-05 | 3.22148E-05 | 8.32469E-05 | 0.000531063 | 0.000171076 | 0.000349744 | 0.000832487 | 0.003361248 |
| GTEX-13O3O | 5.70687E-05 | 1.13532E-05 | 4.11012E-05 | 6.25405E-05 | 0.000454839 | 0.000200449 | 0.000149002 | 0.000343932 | 0.000864079 | 0.004398401 |
| GTEX-13O3P | 4.44768E-05 | 7.49666E-06 | 3.67515E-05 | 4.31475E-05 | 0.000310484 | 0.000185161 | 0.000258982 | 0.000426636 | 0.000672376 | 0.002293526 |
| GTEX-13O3Q | 9.71839E-05 | 1.25822E-05 | 1.81149E-05 | 2.15198E-05 | 5.95587E-05 | 0.000199635 | 7.22969E-05 | 0.00028105 | 0.000596434 | 0.002909532 |
| GTEX-13O61 | 3.9458E-05 | 9.36779E-06 | 5.98849E-05 | 3.32308E-05 | 0.000945041 | 0.000249057 | 0.00012101 | 0.000283295 | 0.00072531 | 0.002232085 |
| GTEX-13OVG | 5.53422E-05 | 1.28508E-05 | 0.000523532 | 6.68325E-05 | 0.000189775 | 0.000261236 | 8.63895E-05 | 0.000265904 | 0.000653943 | 0.003202864 |
| GTEX-13OVI | 8.2875E-05 | 1.29581E-05 | 0.000163784 | 4.5634E-05 | 0.001214604 | 0.00019033 | 0.000159922 | 0.000508732 | 0.001016143 | 0.006454964 |
| GTEX-13OVJ | 5.12375E-05 | 9.39138E-06 | 6.97757E-05 | 0.000117008 | 0.000345189 | 6.50247E-05 | 0.000195007 | 0.000715308 | 0.001083121 | 0.003119717 |
| GTEX-13OVL | 0.000107346 | 9.7027E-06 | 4.39705E-05 | 4.66059E-05 | 1.38609E-05 | 0.000211948 | 0.000174625 | 0.000250946 | 0.000775272 | 0.005133244 |
| GTEX-13OW5 | 7.15677E-05 | 0 | 3.38019E-05 | 2.23489E-05 | 0.000550494 | 0.000123438 | 8.66413E-05 | 0.000411307 | 0.000563227 | 0.002062425 |
| GTEX-13OW6 | 8.65928E-05 | 1.31646E-05 | 4.74929E-05 | 5.03652E-05 | 7.14681E-05 | 0.000133379 | 7.50339E-05 | 0.000279401 | 0.000728132 | 0.0020755 |
| GTEX-13OW7 | 6.0396E-05 | 1.06833E-05 | 2.45628E-05 | 3.01267E-05 | 0.000279442 | 0.000113684 | 8.73168E-05 | 0.000301902 | 0.000491067 | 0.003752906 |
| GTEX-13OW8 | 9.85389E-05 | 6.581E-05 | 3.75741E-05 | 9.42113E-05 | 4.22002E-05 | 0.000227537 | 0.000282962 | 0.000370637 | 0.001556938 | 0.005854465 |
| GTEX-13PDP | 0.000194959 | 3.14606E-05 | 0.00012136 | 4.54094E-05 | 0.00091781 | 0.000216876 | 0.000400673 | 0.000638527 | 0.001107251 | 0.005664584 |
| GTEX-13PL6 | 5.87993E-05 | 8.41604E-06 | 2.45366E-05 | 3.23474E-05 | 9.73083E-05 | 0.000117632 | 0.000160535 | 0.000288012 | 0.000680422 | 0.003043351 |
| GTEX-13PL7 | 5.8568E-05 | 0 | 0.000135664 | 5.30568E-05 | 0.000834087 | 0.000167303 | 9.74841E-05 | 0.000272355 | 0.000622778 | 0.001952344 |
| GTEX-13PVQ | 3.10273E-05 | 1.57595E-05 | 2.46103E-05 | 1.69401E-05 | 8.93029E-05 | 0.000100054 | 6.7796E-05 | 0.000247303 | 0.000503183 | 0.001935426 |
| GTEX-13PVR | 3.7521E-05 | 1.64772E-05 | 3.05393E-05 | 7.69571E-05 | 0.000871532 | 0.000248569 | 0.00011146 | 0.0002773 | 0.000673894 | 0.002913557 |
| GTEX-13QBU | 4.6536E-05 | 7.65842E-06 | 0.000137333 | 3.98775E-05 | 0.000524512 | 0.000179635 | 0.000106599 | 0.000299172 | 0.000809634 | 0.002225487 |
| GTEX-13QIC | 8.99651E-05 | 3.77341E-05 | 4.04308E-05 | 2.33095E-05 | 0.000114399 | 0.000109718 | 0.000117785 | 0.000343551 | 0.00066801 | 0.006376186 |
| GTEX-13QJ3 | 0.000101254 | 1.5566E-05 | 4.09452E-05 | 0.00013669 | 0.00021299 | 0.000136192 | 0.000165024 | 0.000369623 | 0.00101842 | 0.002901347 |
| GTEX-13RTK | 5.89642E-05 | 0 | 0.000106866 | 2.93535E-05 | 0.000120722 | 0.000144238 | 7.27402E-05 | 0.000173077 | 0.000505685 | 0.001330562 |
| GTEX-13S7M | 0.000110149 | 0.000166988 | 7.39774E-05 | 0.000237499 | 6.88815E-05 | 0.000249844 | 0.000152891 | 0.000730625 | 0.001065539 | 0.005857097 |
| GTEX-13S86 | 0.000102217 | 3.47762E-05 | 8.89844E-05 | 7.24054E-05 | 3.17391E-05 | 0.000203114 | 0.000311892 | 0.0005615 | 0.000837787 | 0.006210057 |
| GTEX-13SLW | 0.000191879 | 3.32034E-05 | 0.000321476 | 8.6292E-05 | 0.000103961 | 0.000773219 | 0.000402961 | 0.000635725 | 0.001998301 | 0.013747571 |
| GTEX-13SLX | 0.000110115 | 1.06241E-05 | 6.82557E-05 | 3.0756E-05 | 5.03148E-05 | 0.000108884 | 0.000127184 | 0.000261144 | 0.000984984 | 0.00473144 |
| GTEX-13U4I | 9.33484E-05 | 2.98834E-05 | 0.000104208 | 0.000503407 | 2.93148E-05 | 0.000187002 | 0.000184528 | 0.000305723 | 0.000921169 | 0.00506474 |
| GTEX-13VXT | 7.89083E-05 | 3.10076E-05 | 5.18849E-05 | 0.000221607 | 0.000343441 | 0.000287549 | 0.000144594 | 0.000346417 | 0.000693858 | 0.002421552 |
| GTEX-13W3W | 5.10251E-05 | 1.99566E-05 | 7.13474E-05 | 4.89247E-05 | 1.58588E-05 | 0.000698836 | 9.06046E-05 | 0.000231606 | 0.000642983 | 0.001456951 |
| GTEX-13W46 | 0.000144975 | 7.27739E-05 | 7.75187E-05 | 6.48102E-05 | 0.000446143 | 0.000182651 | 0.000196981 | 0.000403677 | 0.000943733 | 0.003250687 |
| GTEX-13X6I | 0.000194787 | 1.16937E-05 | 0.000194958 | 0.000178385 | 3.31282E-05 | 0.000375882 | 0.000425267 | 0.000966958 | 0.002674722 | 0.012852747 |
| GTEX-13X6K | 7.74082E-05 | 5.52469E-05 | 7.967E-05 | 0.000127474 | 0.000684238 | 0.000238105 | 0.000184135 | 0.000361294 | 0.001180765 | 0.004998121 |
| GTEX-13YAN | 0.000321582 | 0.000131026 | 0.000412191 | 0.000461294 | 0.000109886 | 0.000491586 | 0.000540104 | 0.000871885 | 0.002103313 | 0.013172803 |
| GTEX-1445S | 8.45934E-05 | 1.57148E-05 | 4.45783E-05 | 8.91842E-05 | 0.000205259 | 7.89627E-05 | 0.000155615 | 0.000744933 | 0.000830031 | 0.002150532 |
| GTEX-144FL | 0.000179586 | 5.59449E-05 | 0.000407559 | 0.000276997 | 0.000179115 | 0.000552875 | 0.000503917 | 0.000637692 | 0.001592878 | 0.010566321 |
| GTEX-144GL | 7.39976E-05 | 1.39802E-05 | 3.69435E-05 | 2.3346E-05 | 4.92248E-05 | 0.000145356 | 0.00010061 | 0.000262288 | 0.000572739 | 0.004452314 |
| GTEX-144GM | 4.07324E-05 | 5.76453E-06 | 1.9425E-05 | 3.73011E-05 | 0.000127225 | 7.10134E-05 | 0.000144697 | 0.000406661 | 0.000374611 | 0.001583802 |
| GTEX-144GN | 6.33299E-05 | 3.4385E-06 | 6.58972E-05 | 8.1328E-05 | 9.10726E-05 | 0.000227226 | 0.000162292 | 0.000343759 | 0.000852817 | 0.002392943 |
| GTEX-144GO | 0.000109105 | 9.43497E-06 | 9.47844E-05 | 3.48238E-05 | 0.000258474 | 0.000233558 | 0.000224242 | 0.000546772 | 0.000980405 | 0.005096528 |
| GTEX-145LS | 0.000111809 | 5.37799E-05 | 5.7758E-05 | 0.000434484 | 7.15259E-05 | 0.000233831 | 0.000228958 | 0.000542149 | 0.000794502 | 0.003536612 |
| GTEX-145LT | 5.05105E-05 | 7.61334E-06 | 5.8661E-05 | 2.61875E-05 | 0.000386956 | 0.000233854 | 0.000107485 | 0.000405447 | 0.000671132 | 0.002085224 |
| GTEX-145LU | 7.28704E-05 | 2.05965E-05 | 2.76102E-05 | 1.78203E-05 | 0.000114924 | 0.000181513 | 0.00011817 | 0.000303731 | 0.0007651 | 0.003771046 |
| GTEX-145ME | 5.20429E-05 | 5.29863E-06 | 2.01091E-05 | 3.03667E-05 | 7.84828E-05 | 0.000173909 | 0.000124565 | 0.000237481 | 0.000723599 | 0.003273537 |
| GTEX-145MH | 6.5414E-05 | 1.13625E-05 | 4.48732E-05 | 6.30197E-05 | 8.42351E-05 | 0.000133748 | 9.97991E-05 | 0.000244151 | 0.000701687 | 0.0027879 |
| GTEX-145MI | 4.97484E-05 | 3.30735E-06 | 1.7598E-05 | 0.000166152 | 3.19214E-05 | 8.15817E-05 | 0.000143783 | 0.000294534 | 0.000717198 | 0.001884927 |
| GTEX-145MO | 6.69722E-05 | 9.9449E-06 | 5.13828E-05 | 0.00010138 | 0.000422405 | 0.000166531 | 0.000126839 | 0.000269214 | 0.000786903 | 0.003144327 |
| GTEX-146FQ | 0.000179558 | 2.96723E-05 | 0.00018317 | 8.01236E-05 | 0.000130271 | 0.000180585 | 0.000516832 | 0.0003902 | 0.001074068 | 0.009877393 |
| GTEX-146FR | 7.02874E-05 | 4.20517E-05 | 9.82364E-05 | 3.52965E-05 | 0.001150218 | 0.000384929 | 0.000211082 | 0.00030988 | 0.001008631 | 0.004077809 |
| GTEX-14753 | 3.42035E-05 | 1.73532E-05 | 3.24924E-05 | 1.06782E-05 | 0.000790856 | 0.00013869 | 0.000105142 | 0.000274821 | 0.000452297 | 0.002125919 |
| GTEX-1477Z | 9.51981E-05 | 5.79606E-05 | 7.0223E-05 | 0.000308804 | 7.8985E-05 | 0.000171808 | 0.000229015 | 0.000481804 | 0.001380251 | 0.004691266 |
| GTEX-147F3 | 5.49856E-05 | 3.47716E-06 | 4.00459E-05 | 2.83901E-05 | 9.96556E-05 | 0.00016451 | 0.000126568 | 0.000204008 | 0.000672848 | 0.00702909 |
| GTEX-148VJ | 7.10831E-05 | 4.50252E-06 | 5.90524E-05 | 0.000104513 | 8.327E-05 | 0.000149993 | 0.000191531 | 0.000312738 | 0.001101091 | 0.003811311 |
| GTEX-14A5H | 6.81214E-05 | 4.86694E-05 | 8.78465E-05 | 8.32417E-05 | 0.000108663 | 0.000107289 | 0.000218308 | 0.000533415 | 0.000545974 | 0.005399399 |
| GTEX-14A5I | 6.72221E-05 | 3.18359E-06 | 3.90882E-05 | 6.17546E-05 | 0.000841608 | 0.000130421 | 0.000114263 | 0.000395528 | 0.001038235 | 0.003425411 |
| GTEX-14A6H | 0.00014697 | 0.000149508 | 4.32148E-05 | 6.26529E-05 | 7.11178E-05 | 0.000125503 | 0.000283886 | 0.000438896 | 0.000936662 | 0.005997929 |
| GTEX-14AS3 | 6.90966E-05 | 2.22554E-05 | 9.23702E-05 | 2.17912E-05 | 0.000363352 | 9.41988E-05 | 0.000129868 | 0.000332595 | 0.00082718 | 0.00239707 |
| GTEX-14ASI | 0.000269319 | 4.09009E-05 | 0.000500364 | 0.002473738 | 7.77707E-05 | 0.000645169 | 0.000655993 | 0.000501951 | 0.001713385 | 0.041845852 |
| GTEX-14BIL | 5.88833E-05 | 2.73333E-05 | 2.38469E-05 | 0.000133814 | 0.00019581 | 0.000143924 | 0.000132067 | 0.000354917 | 0.000739321 | 0.003121959 |
| GTEX-14BIM | 8.7909E-05 | 3.67329E-06 | 7.13427E-05 | 6.72749E-05 | 0.000481443 | 0.000233527 | 0.000324674 | 0.000386894 | 0.00070901 | 0.00495562 |
| GTEX-14BMU | 3.60835E-05 | 3.33771E-06 | 1.8844E-05 | 1.13886E-05 | 0.000426359 | 0.000240031 | 8.75907E-05 | 0.00025368 | 0.000647259 | 0.00102753 |
| GTEX-14BMV | 5.3816E-05 | 8.36924E-06 | 2.01701E-05 | 2.82055E-05 | 5.45502E-05 | 8.49689E-05 | 5.9044E-05 | 0.000173993 | 0.000400049 | 0.001377051 |
| GTEX-14C39 | 3.1676E-05 | 6.25649E-06 | 0.000128821 | 5.14492E-05 | 0.000542636 | 0.000156753 | 0.000179044 | 0.000335338 | 0.001042705 | 0.002814832 |
| GTEX-14ICL | 5.13762E-05 | 0 | 0.000267557 | 5.17331E-05 | 0.001755801 | 0.000210094 | 6.18738E-05 | 0.000497291 | 0.000814471 | 0.001137696 |
| GTEX-N7MS- | 0.000137505 | 9.26961E-05 | 5.03742E-05 | 3.63529E-05 | 9.06585E-05 | 0.00024347 | 0.000150175 | 0.000329572 | 0.000766433 | 0.006639854 |
| GTEX-NFK9- | 7.40976E-05 | 1.2292E-05 | 6.26126E-05 | 5.55761E-05 | 0.000815132 | 0.000214167 | 0.000113712 | 0.00020634 | 0.000622447 | 0.002403189 |
| GTEX-NPJ8- | 0.000125859 | 4.85777E-05 | 0.000116661 | 0.000119495 | 0.001752265 | 0.000140821 | 0.000170174 | 0.000963759 | 0.000986824 | 0.002003703 |
| GTEX-O5YT- | 4.93833E-05 | 1.73034E-05 | 3.67051E-05 | 3.97719E-05 | 0.000439539 | 0.00016107 | 0.000146689 | 0.000411891 | 0.000803342 | 0.001907425 |
| GTEX-O5YV- | 4.38161E-05 | 2.47784E-05 | 0.000220805 | 9.46555E-05 | 9.93894E-05 | 0.000201739 | 0.000197333 | 0.000452402 | 0.000833907 | 0.002206903 |
| GTEX-OHPJ- | 3.7435E-05 | 1.0093E-05 | 1.88029E-05 | 4.22798E-05 | 0.000163856 | 0.000113346 | 5.48897E-05 | 0.000250238 | 0.000503269 | 0.001212658 |
| GTEX-OHPK- | 4.74573E-05 | 1.32671E-05 | 3.91222E-05 | 0.000124957 | 0.001085933 | 0.000169414 | 6.20392E-05 | 0.000223296 | 0.000544129 | 0.001443217 |
| GTEX-OHPL- | 7.2155E-05 | 3.18191E-05 | 8.71728E-05 | 9.90452E-05 | 0.000122369 | 0.000176809 | 0.00019816 | 0.000435643 | 0.000746808 | 0.004456582 |
| GTEX-OIZG- | 5.98372E-05 | 0 | 4.62417E-05 | 5.1375E-05 | 3.56574E-05 | 6.06568E-05 | 8.2126E-05 | 0.000311367 | 0.000569493 | 0.001477764 |
| GTEX-OIZH- | 4.08893E-05 | 2.77107E-06 | 3.18385E-05 | 0.000104578 | 0.000239983 | 0.000118098 | 9.12273E-05 | 0.000300449 | 0.000459508 | 0.00132482 |
| GTEX-OIZI- | 4.39641E-05 | 6.56226E-06 | 2.49906E-05 | 5.371E-05 | 0.00040384 | 0.000185322 | 0.000166066 | 0.000288635 | 0.00071907 | 0.002173397 |
| GTEX-OOBJ- | 4.74256E-05 | 1.59913E-05 | 3.84068E-05 | 3.58503E-05 | 0.000820985 | 9.59802E-05 | 6.91462E-05 | 0.000363683 | 0.000554797 | 0.001183943 |
| GTEX-OOBK- | 4.16118E-05 | 5.57864E-06 | 5.10755E-05 | 0.000137737 | 0.000588357 | 7.98736E-05 | 6.71555E-05 | 0.000394973 | 0.000543977 | 0.001552407 |
| GTEX-OXRK- | 5.80752E-05 | 1.86295E-05 | 3.74295E-05 | 0.000129701 | 0.000763197 | 0.000232634 | 0.000118748 | 0.000365325 | 0.000548039 | 0.00178785 |
| GTEX-OXRL- | 7.03376E-05 | 1.46778E-05 | 4.3243E-05 | 9.69526E-06 | 0.000513188 | 0.000303664 | 7.6743E-05 | 0.000312005 | 0.000604683 | 0.002270318 |
| GTEX-OXRN- | 8.13098E-05 | 2.72221E-05 | 3.74115E-05 | 0.0001334 | 0.000344364 | 0.000117526 | 0.000133884 | 0.000532797 | 0.000833552 | 0.003796235 |
| GTEX-OXRO- | 0.000254176 | 0.000110468 | 0.000126832 | 0.000371395 | 0.000759586 | 0.000523688 | 0.000500207 | 0.000841924 | 0.002552677 | 0.023772918 |
| GTEX-OXRP- | 4.13425E-05 | 3.51799E-05 | 2.67384E-05 | 2.34128E-05 | 0.000247271 | 0.000145163 | 9.54004E-05 | 0.000345782 | 0.000664976 | 0.0041623 |
| GTEX-P44G- | 9.00832E-05 | 4.88823E-05 | 3.34708E-05 | 8.61045E-05 | 1.16515E-05 | 0.000164594 | 0.00014073 | 0.000340679 | 0.000841748 | 0.005984009 |
| GTEX-P4PP- | 0.000114055 | 4.26472E-05 | 5.94509E-05 | 0.000118183 | 2.61697E-05 | 0.000146417 | 0.000163809 | 0.000452474 | 0.000908099 | 0.004097331 |
| GTEX-P4PQ- | 5.98826E-05 | 3.17124E-05 | 0.000222832 | 5.50302E-05 | 0.000247397 | 0.000163944 | 9.46575E-05 | 0.000375198 | 0.000808196 | 0.002810913 |
| GTEX-P4QS- | 4.42259E-05 | 0 | 4.66809E-05 | 7.42869E-05 | 0.001139579 | 0.00024406 | 7.83882E-05 | 0.000376275 | 0.000733149 | 0.002321039 |
| GTEX-P4QT- | 6.40386E-05 | 5.80636E-06 | 7.40242E-05 | 5.12466E-05 | 0.000646682 | 0.000143157 | 8.76612E-05 | 0.000382434 | 0.000753379 | 0.001979932 |
| GTEX-P78B- | 5.91522E-05 | 1.3221E-05 | 3.68709E-05 | 1.67719E-05 | 0.000174023 | 8.15535E-05 | 7.37169E-05 | 0.000365454 | 0.000758757 | 0.001312654 |
| GTEX-PLZ6- | 8.84958E-05 | 0.000101208 | 4.26601E-05 | 2.73007E-05 | 0.00092203 | 0.000299852 | 0.000153739 | 0.000413088 | 0.000735808 | 0.003122035 |
| GTEX-POMQ- | 0.000107019 | 2.7907E-05 | 9.03868E-05 | 9.33521E-05 | 7.68622E-05 | 0.000207043 | 0.000181723 | 0.000398027 | 0.000851637 | 0.003640608 |
| GTEX-POYW- | 8.71307E-05 | 6.81182E-05 | 0.000947926 | 0.000183459 | 0.000264582 | 0.00019789 | 0.00064714 | 0.001048897 | 0.002073043 | 0.004999882 |
| GTEX-PSDG- | 9.02422E-05 | 1.8736E-05 | 6.36112E-05 | 0.000136469 | 0.000472042 | 0.000216634 | 0.000114308 | 0.000287673 | 0.00084197 | 0.00276885 |
| GTEX-PW2O- | 8.9557E-05 | 0 | 2.90295E-05 | 8.05718E-05 | 0.000202111 | 7.78477E-05 | 6.42248E-05 | 0.000348006 | 0.000689436 | 0.00202708 |
| GTEX-PWCY- | 3.52694E-05 | 0 | 2.03005E-05 | 2.01652E-05 | 0.000455634 | 0.000173975 | 0.000105729 | 0.000193187 | 0.000444635 | 0.000975896 |
| GTEX-PWN1- | 3.59505E-05 | 0 | 5.19796E-05 | 1.84379E-05 | 0.000588686 | 0.000261796 | 0.000166088 | 0.000298284 | 0.00079013 | 0.002338203 |
| GTEX-PWOO- | 5.29538E-05 | 2.14047E-05 | 0.000115328 | 3.77542E-05 | 1.70588E-05 | 0.000246486 | 0.000135546 | 0.00045053 | 0.000871138 | 0.004893684 |
| GTEX-PX3G- | 0.000161719 | 0 | 9.71554E-05 | 6.9748E-05 | 0.00017193 | 0.000162744 | 0.000148778 | 0.000565583 | 0.000967078 | 0.003127504 |
| GTEX-Q2AG- | 0.000105838 | 1.26298E-05 | 5.82149E-05 | 6.78935E-06 | 0.000513308 | 0.000249492 | 9.92975E-05 | 0.000404572 | 0.000913513 | 0.004680174 |
| GTEX-Q2AH- | 7.88345E-05 | 2.9841E-05 | 5.74008E-05 | 0.000649003 | 0.000843112 | 0.000225341 | 0.000214175 | 0.000578282 | 0.001013792 | 0.00323258 |
| GTEX-Q2AI- | 3.18137E-05 | 1.03746E-05 | 5.05078E-05 | 3.65664E-05 | 0.000112721 | 0.000207205 | 0.00031071 | 0.000537993 | 0.001043974 | 0.002660547 |
| GTEX-Q734- | 0.000140821 | 1.02594E-05 | 9.64302E-05 | 0.000119804 | 3.54586E-05 | 0.000152822 | 0.000285893 | 0.00067995 | 0.001368668 | 0.002520987 |
| GTEX-QCQG- | 5.65487E-05 | 1.44774E-05 | 5.27511E-05 | 8.75806E-06 | 0.000169471 | 0.000157266 | 8.18262E-05 | 0.000309627 | 0.00078844 | 0.001677183 |
| GTEX-QDT8- | 8.54234E-05 | 1.30001E-05 | 4.94233E-05 | 3.89871E-05 | 9.1168E-05 | 0.00017731 | 0.000212346 | 0.000483895 | 0.001216916 | 0.006492248 |
| GTEX-QDVJ- | 6.80758E-05 | 0 | 9.05859E-05 | 4.33125E-05 | 2.77187E-05 | 0.00015898 | 0.000162196 | 0.000524103 | 0.001283844 | 0.002587149 |
| GTEX-QDVN- | 0.000103131 | 3.29346E-05 | 0.000129526 | 4.91239E-05 | 0.000105127 | 0.000290141 | 0.000162253 | 0.000838749 | 0.001769613 | 0.005893746 |
| GTEX-QEG4- | 7.10629E-05 | 0 | 2.29441E-05 | 1.88709E-05 | 0.000296677 | 0.000135676 | 0.000120842 | 0.000328371 | 0.000792247 | 0.002855525 |
| GTEX-QEL4- | 4.79998E-05 | 0 | 6.48644E-05 | 2.50557E-05 | 0.000293696 | 0.000122639 | 0.000152858 | 0.000347264 | 0.001064735 | 0.000952463 |
| GTEX-QESD- | 3.87926E-05 | 8.42011E-05 | 1.67911E-05 | 1.6634E-05 | 0.000182244 | 9.73488E-05 | 0.00016386 | 0.000315634 | 0.000420832 | 0.00256208 |
| GTEX-QLQ7- | 7.27222E-05 | 1.64775E-05 | 7.12629E-05 | 1.79886E-05 | 0.000698938 | 0.000306057 | 0.00017842 | 0.000396937 | 0.000927479 | 0.004723277 |
| GTEX-QLQW- | 7.31473E-05 | 1.24523E-05 | 4.22804E-05 | 2.87903E-05 | 8.28249E-05 | 0.000230989 | 0.000133632 | 0.00034636 | 0.000846568 | 0.003177552 |
| GTEX-QV31- | 3.86828E-05 | 0 | 6.69407E-05 | 4.67076E-05 | 0.000602161 | 0.000253682 | 0.000111377 | 0.000234952 | 0.000504945 | 0.001899541 |
| GTEX-QV44- | 8.33398E-05 | 2.58351E-05 | 7.27489E-05 | 5.35325E-05 | 0.00053605 | 0.000117977 | 0.000100993 | 0.000656456 | 0.000827357 | 0.001928954 |
| GTEX-QVJO- | 0.000100577 | 0 | 7.04678E-05 | 4.12E-05 | 9.03587E-05 | 0.000169106 | 0.000244127 | 0.000582133 | 0.001462526 | 0.004548583 |
| GTEX-QXCU- | 7.32869E-05 | 1.7114E-05 | 2.84977E-05 | 8.71823E-05 | 0.000108479 | 0.000107058 | 8.29388E-05 | 0.000265626 | 0.000722894 | 0.003906965 |
| GTEX-R3RS- | 0.000162633 | 9.51041E-05 | 0.000134768 | 0.000415152 | 0.000115724 | 0.000128761 | 0.000222193 | 0.000662797 | 0.001793165 | 0.004711096 |
| GTEX-R53T- | 6.77778E-05 | 0 | 4.34041E-05 | 4.37345E-05 | 0.000152181 | 8.12395E-05 | 0.000111367 | 0.000281896 | 0.000478322 | 0.000953146 |
| GTEX-R55C- | 2.79204E-05 | 3.70391E-06 | 2.52726E-05 | 3.26808E-05 | 0.000347607 | 0.000250696 | 3.63067E-05 | 0.000161289 | 0.000360067 | 0.000760942 |
| GTEX-R55D- | 7.78467E-05 | 3.85629E-05 | 0.000344115 | 3.96801E-05 | 0.000856368 | 0.00039974 | 0.000159755 | 0.000550211 | 0.000993406 | 0.002004466 |
| GTEX-R55E- | 8.36034E-05 | 6.95779E-06 | 3.6248E-05 | 2.44451E-05 | 6.43421E-05 | 0.000111779 | 0.00012384 | 0.000294119 | 0.000779552 | 0.002315395 |
| GTEX-R55F- | 8.93181E-05 | 1.0841E-05 | 2.4485E-05 | 2.70603E-05 | 0.000907079 | 0.000215822 | 0.000187358 | 0.000654859 | 0.000737821 | 0.003201212 |
| GTEX-R55G- | 5.12749E-05 | 7.01331E-06 | 0.000136335 | 4.85906E-05 | 5.75231E-05 | 0.00037042 | 0.000177438 | 0.000322583 | 0.000912268 | 0.002469517 |
| GTEX-REY6- | 5.12992E-05 | 4.5695E-06 | 2.33857E-05 | 6.70145E-05 | 0.00069045 | 0.000180826 | 0.000106425 | 0.000465055 | 0.00110176 | 0.004157698 |
| GTEX-RM2N- | 5.98131E-05 | 1.9318E-05 | 6.26899E-05 | 2.3953E-05 | 0.000763801 | 0.000469633 | 0.000120602 | 0.000512524 | 0.000844348 | 0.003463543 |
| GTEX-RN64- | 5.17601E-05 | 0 | 8.53965E-06 | 2.10514E-05 | 7.18398E-05 | 0.000103477 | 7.52915E-05 | 0.000260009 | 0.000532502 | 0.002848762 |
| GTEX-RNOR- | 6.85604E-05 | 9.09668E-06 | 2.27233E-05 | 1.98206E-05 | 0.000110387 | 0.000135665 | 0.000119933 | 0.000419165 | 0.001074852 | 0.002988693 |
| GTEX-RTLS- | 7.53273E-05 | 5.68675E-05 | 5.1796E-05 | 2.74042E-05 | 0.000191712 | 0.000127278 | 0.000161548 | 0.00040765 | 0.000905323 | 0.003339942 |
| GTEX-RU1J- | 6.68586E-05 | 0 | 5.58878E-05 | 5.7402E-05 | 0.000558026 | 0.000258906 | 0.000118126 | 0.000314813 | 0.000678371 | 0.002247431 |
| GTEX-RU72- | 9.47015E-05 | 1.31989E-05 | 5.61603E-05 | 2.44084E-05 | 0.000409693 | 0.00023898 | 0.000162441 | 0.000403643 | 0.000969684 | 0.004606371 |
| GTEX-RUSQ- | 3.95736E-05 | 2.38409E-05 | 5.26861E-05 | 7.86181E-05 | 0.000326173 | 9.00537E-05 | 6.94213E-05 | 0.000952019 | 0.000928737 | 0.001627776 |
| GTEX-RVPU- | 4.03226E-05 | 0 | 4.45184E-05 | 2.27923E-05 | 0.000532547 | 0.000144933 | 8.74581E-05 | 0.000324052 | 0.000726552 | 0.001755385 |
| GTEX-RVPV- | 9.34662E-05 | 1.82004E-05 | 5.67479E-05 | 5.77232E-05 | 4.06041E-05 | 9.91757E-05 | 9.30659E-05 | 0.00020363 | 0.000369173 | 0.004907456 |
| GTEX-RWS6- | 6.28922E-05 | 2.55582E-05 | 3.39435E-05 | 2.30639E-05 | 0.000423863 | 0.000306873 | 0.000225894 | 0.000275728 | 0.000871342 | 0.004065214 |
| GTEX-S32W- | 3.47827E-05 | 2.09426E-05 | 3.96889E-05 | 3.94655E-05 | 0.000206495 | 0.000176459 | 0.000121471 | 0.00044183 | 0.001186993 | 0.002394827 |
| GTEX-S33H- | 8.59774E-05 | 0 | 9.25531E-05 | 1.89067E-05 | 4.54515E-05 | 0.000106048 | 0.000114624 | 0.000201375 | 0.000547434 | 0.002807256 |
| GTEX-S3XE- | 4.80235E-05 | 2.43585E-05 | 5.73505E-05 | 0.000188863 | 0.000120281 | 0.000183516 | 8.21872E-05 | 0.000349876 | 0.000642946 | 0.002095481 |
| GTEX-S4P3- | 5.32331E-05 | 2.24329E-05 | 9.48701E-05 | 0.00022325 | 0.001109938 | 0.000353565 | 0.000125996 | 0.000230886 | 0.000466887 | 0.001793282 |
| GTEX-S4Z8- | 5.95154E-05 | 2.27891E-05 | 9.25804E-05 | 0.000123345 | 0.000719859 | 0.000449978 | 0.000299462 | 0.000514746 | 0.000820972 | 0.004138772 |
| GTEX-S7PM- | 6.87188E-05 | 1.51632E-05 | 2.63634E-05 | 0.000102856 | 0.000397126 | 0.000168778 | 0.000156956 | 0.000323947 | 0.000993891 | 0.006067331 |
| GTEX-S7SF- | 4.29269E-05 | 0 | 3.27799E-05 | 8.87627E-05 | 0.000508521 | 9.33298E-05 | 7.1264E-05 | 0.000325245 | 0.000763305 | 0.002109443 |
| GTEX-S95S- | 5.40049E-05 | 2.21475E-05 | 4.49266E-05 | 3.48171E-05 | 0.000364924 | 0.000357537 | 0.000114475 | 0.000279767 | 0.000853372 | 0.001630732 |
| GTEX-SIU7- | 0.00014311 | 9.28953E-05 | 0.000322612 | 0.000215059 | 0.000836739 | 0.00077677 | 0.000335762 | 0.000380352 | 0.000943781 | 0.015173998 |
| GTEX-SJXC- | 7.23074E-05 | 8.4747E-06 | 5.00853E-05 | 0.000220154 | 0.000483209 | 0.000261737 | 0.000221557 | 0.000474397 | 0.000924931 | 0.006261956 |
| GTEX-SNMC- | 5.5129E-05 | 3.30863E-06 | 5.48455E-05 | 2.17515E-05 | 0.000427331 | 0.000233294 | 0.00010916 | 0.0004134 | 0.000639414 | 0.001416609 |
| GTEX-SSA3- | 0.000179848 | 0.000106757 | 0.000100163 | 0.004549109 | 0.000203147 | 0.000290107 | 0.000434047 | 0.000542877 | 0.001127241 | 0.00800001 |
| GTEX-SUCS- | 7.97081E-05 | 3.03893E-05 | 0.000170378 | 3.27962E-05 | 0.000217884 | 0.000308845 | 0.000122603 | 0.000352227 | 0.001019176 | 0.003502533 |
| GTEX-T2YK- | 0.000261776 | 5.06545E-05 | 0.000198024 | 0.000257805 | 6.91403E-05 | 0.000239364 | 0.000371657 | 0.000401276 | 0.000894448 | 0.00487441 |
| GTEX-T5JC- | 0.000101877 | 2.57348E-06 | 5.0749E-05 | 4.31842E-05 | 9.32952E-05 | 0.000269981 | 0.000158823 | 0.000390936 | 0.001057377 | 0.003086011 |
| GTEX-T5JW- | 8.76262E-05 | 1.27305E-05 | 4.16743E-05 | 3.59898E-05 | 0.000308426 | 0.000425985 | 0.000165421 | 0.000421574 | 0.001183744 | 0.002918475 |
| GTEX-TKQ2- | 4.76207E-05 | 1.54069E-05 | 6.04837E-05 | 2.77384E-05 | 1.40251E-05 | 0.000115487 | 0.000130349 | 0.000235306 | 0.00045339 | 0.001644801 |
| GTEX-TMZS- | 8.66602E-05 | 1.69222E-05 | 6.78268E-05 | 7.11687E-05 | 0.00081736 | 0.00026955 | 0.000170327 | 0.000297994 | 0.000504208 | 0.002317278 |
| GTEX-U3ZH- | 0.000189344 | 2.36794E-05 | 0.000119511 | 0.000163555 | 0.000348548 | 0.000181969 | 0.000178976 | 0.000750111 | 0.00220166 | 0.00760885 |
| GTEX-U3ZM- | 6.37775E-05 | 4.88058E-06 | 0.000138513 | 3.08128E-05 | 0.000297936 | 0.000485922 | 0.000143582 | 0.000323366 | 0.001201756 | 0.003182651 |
| GTEX-U412- | 9.33987E-05 | 1.18402E-05 | 4.69811E-05 | 4.47484E-05 | 0.000509469 | 0.000178383 | 0.000102487 | 0.000229125 | 0.000735475 | 0.003238659 |
| GTEX-U4B1- | 6.10208E-05 | 2.76842E-05 | 6.03981E-05 | 3.52162E-05 | 0.001263936 | 0.00024416 | 0.000136414 | 0.000318577 | 0.000814815 | 0.002611212 |
| GTEX-U8XE- | 3.74144E-05 | 1.97198E-05 | 4.96291E-05 | 9.23012E-06 | 9.13384E-05 | 8.98248E-05 | 9.22317E-05 | 0.000578208 | 0.000512117 | 0.00131756 |
| GTEX-UJMC- | 3.9294E-05 | 2.39104E-05 | 2.35502E-05 | 2.39087E-05 | 0.001062303 | 0.000285275 | 0.000106498 | 0.000410873 | 0.000689853 | 0.002226065 |
| GTEX-UPJH- | 5.72706E-05 | 1.32777E-05 | 2.14799E-05 | 4.42322E-05 | 0.000678691 | 0.000169982 | 0.000102327 | 0.00022707 | 0.000612836 | 0.002175923 |
| GTEX-VJYA- | 2.98686E-05 | 0 | 0.000129437 | 0.000110468 | 0.000108194 | 0.000111405 | 0.000235421 | 0.000579278 | 0.001062713 | 0.003698712 |
| GTEX-VUSG- | 0.000221506 | 3.48953E-05 | 0.00015401 | 0.000420226 | 0.001105078 | 0.000799731 | 0.00067588 | 0.000595281 | 0.001421416 | 0.012078099 |
| GTEX-VUSH- | 9.03537E-05 | 1.53519E-05 | 6.08384E-05 | 0.000115127 | 9.67011E-05 | 0.000178747 | 0.000147338 | 0.000470851 | 0.000913721 | 0.018139348 |
| GTEX-WEY5- | 3.54765E-05 | 0 | 3.79151E-05 | 4.07689E-05 | 0.000180029 | 0.000307789 | 7.48148E-05 | 0.000187062 | 0.0007063 | 0.001034889 |
| GTEX-WFG8- | 1.74108E-05 | 0 | 0.000164306 | 7.91355E-05 | 0.000368419 | 0.00019839 | 0.000178127 | 0.000385259 | 0.000693357 | 0.003084977 |
| GTEX-WFON- | 5.83108E-05 | 1.25252E-05 | 0.000135942 | 1.92638E-05 | 0.001420659 | 0.000381422 | 0.000132078 | 0.000301477 | 0.000703667 | 0.003399173 |
| GTEX-WHPG- | 6.45692E-05 | 2.39263E-05 | 3.02616E-05 | 7.67992E-05 | 0.000194641 | 0.000173194 | 0.000263661 | 0.000349486 | 0.000662327 | 0.002635089 |
| GTEX-WHSE- | 8.36412E-05 | 2.91606E-05 | 4.63039E-05 | 5.00728E-05 | 3.69483E-05 | 0.00016283 | 0.00033343 | 0.000397334 | 0.000911839 | 0.015172828 |
| GTEX-WK11- | 6.7159E-05 | 5.57334E-06 | 3.65932E-05 | 5.81966E-05 | 0.000189838 | 0.000168123 | 0.00010759 | 0.000297851 | 0.000618272 | 0.003682768 |
| GTEX-WL46- | 0.000128565 | 8.15506E-06 | 0.000101961 | 8.08691E-05 | 0.000127613 | 0.000178142 | 0.000219561 | 0.000344145 | 0.000899852 | 0.004631228 |
| GTEX-WOFL- | 0.000152806 | 3.99599E-05 | 0.000169661 | 0.000150599 | 4.86128E-05 | 0.000221994 | 0.000543777 | 0.000772783 | 0.002339018 | 0.009767321 |
| GTEX-WOFM- | 7.05278E-05 | 1.85318E-05 | 9.57629E-05 | 4.26189E-05 | 0.001799732 | 0.000168515 | 0.000110356 | 0.000301127 | 0.000723242 | 0.001537542 |
| GTEX-WRHK- | 8.67539E-05 | 1.72976E-05 | 5.60873E-05 | 3.36816E-05 | 0.000803448 | 0.000248786 | 0.000188946 | 0.000278266 | 0.000656417 | 0.003166958 |
| GTEX-WRHU- | 0.000107776 | 2.46496E-05 | 3.85544E-05 | 2.89565E-05 | 7.08417E-05 | 0.000253542 | 0.000202451 | 0.000326079 | 0.001294337 | 0.012405141 |
| GTEX-WWTW- | 0.000152899 | 0 | 0.000558297 | 0.000299391 | 0.000213349 | 0.000354458 | 0.000468801 | 0.001011662 | 0.001219033 | 0.00394112 |
| GTEX-WWYW- | 8.47029E-05 | 7.42078E-06 | 3.19924E-05 | 8.84574E-05 | 0.000172554 | 0.000154429 | 0.000486669 | 0.000556674 | 0.0006594 | 0.003870104 |
| GTEX-WXYG- | 8.43521E-05 | 3.18836E-06 | 4.92397E-05 | 0.00016025 | 0.000765634 | 0.000470366 | 0.000251205 | 0.000267362 | 0.000661977 | 0.001570512 |
| GTEX-WY7C- | 5.20206E-05 | 2.98238E-06 | 7.64149E-05 | 5.71935E-05 | 0.000192557 | 0.000357888 | 0.000131316 | 0.000380318 | 0.000801759 | 0.002053987 |
| GTEX-WYJK- | 5.52895E-05 | 1.15995E-05 | 0.000131767 | 5.6333E-05 | 0.001445649 | 0.000184053 | 6.36092E-05 | 0.000286628 | 0.000640428 | 0.001395225 |
| GTEX-WYVS- | 5.34051E-05 | 2.13037E-06 | 3.92749E-05 | 6.72827E-05 | 4.32534E-05 | 7.91191E-05 | 6.9195E-05 | 0.000230317 | 0.000565192 | 0.001649896 |
| GTEX-WZTO- | 7.39449E-05 | 2.26004E-05 | 2.95985E-05 | 6.12916E-05 | 8.01204E-05 | 0.000128696 | 0.00010264 | 0.000284423 | 0.000773082 | 0.002985399 |
| GTEX-X261- | 5.93744E-05 | 1.10299E-05 | 0.000110615 | 7.87406E-05 | 0.001690555 | 0.000105661 | 0.000100631 | 0.000417222 | 0.00105246 | 0.002008523 |
| GTEX-X4EO- | 8.15697E-05 | 2.74644E-05 | 5.01689E-05 | 0.00015012 | 3.06739E-05 | 0.000145453 | 0.000132922 | 0.000261102 | 0.000675197 | 0.003471639 |
| GTEX-X4XX- | 0.000145046 | 4.76692E-05 | 7.49964E-05 | 0.000524086 | 0.000698634 | 0.000237171 | 0.000355583 | 0.00096913 | 0.001542545 | 0.007092795 |
| GTEX-X4XY- | 4.21673E-05 | 1.86715E-05 | 5.60601E-05 | 4.91591E-05 | 4.36967E-05 | 0.000114 | 0.00012314 | 0.000342366 | 0.000862153 | 0.004358827 |
| GTEX-X5EB- | 5.23118E-05 | 2.51524E-05 | 2.54792E-05 | 1.9641E-05 | 0.001088317 | 0.000209022 | 5.3711E-05 | 0.000210441 | 0.000680294 | 0.001797676 |
| GTEX-X88G- | 0.000294158 | 2.01293E-05 | 0.000353102 | 0.00083941 | 0.000243625 | 0.001103745 | 0.000390822 | 0.000650013 | 0.001363302 | 0.005236383 |
| GTEX-XAJ8- | 7.95678E-05 | 6.58047E-06 | 8.01659E-05 | 5.50539E-05 | 0.000125101 | 0.000385725 | 0.000137141 | 0.0002425 | 0.000500906 | 0.00235262 |
| GTEX-XBEC- | 5.40147E-05 | 3.23103E-06 | 2.74816E-05 | 4.63005E-05 | 0.000390235 | 0.000143232 | 6.58196E-05 | 0.000196338 | 0.000572861 | 0.002502516 |
| GTEX-XBED- | 5.28208E-05 | 7.47545E-06 | 2.82188E-05 | 6.71491E-05 | 0.000239566 | 0.000181076 | 9.61953E-05 | 0.00033864 | 0.001007332 | 0.002288958 |
| GTEX-XBEW- | 4.42757E-05 | 6.77851E-06 | 0.00010296 | 2.5016E-05 | 0.000589499 | 0.000315068 | 5.93237E-05 | 0.000270376 | 0.000479425 | 0.00123268 |
| GTEX-XGQ4- | 8.00043E-05 | 8.9182E-06 | 9.06732E-05 | 9.01924E-05 | 0.001133312 | 0.000209898 | 0.00012074 | 0.000265135 | 0.000712052 | 0.002861354 |
| GTEX-XOT4- | 8.60601E-05 | 4.39041E-05 | 0.000110691 | 8.16427E-05 | 0.000268025 | 0.000185897 | 0.000184989 | 0.000314254 | 0.000854255 | 0.003309514 |
| GTEX-XOTO- | 9.81286E-05 | 1.29827E-05 | 5.39156E-05 | 7.01845E-05 | 5.74583E-05 | 0.000236709 | 0.000114129 | 0.00028393 | 0.000733397 | 0.004310322 |
| GTEX-XPT6- | 8.39211E-05 | 2.86822E-06 | 7.51739E-05 | 2.55321E-05 | 0.000743602 | 0.000174885 | 0.000135623 | 0.000309124 | 0.000705771 | 0.001765617 |
| GTEX-XPVG- | 3.80322E-05 | 1.02301E-05 | 7.00405E-05 | 3.93229E-05 | 0.000701553 | 0.00022659 | 8.7425E-05 | 0.000207581 | 0.000508356 | 0.002092731 |
| GTEX-XQ3S- | 5.06701E-05 | 6.61951E-05 | 3.15417E-05 | 2.32228E-05 | 3.22315E-05 | 0.000127279 | 5.81616E-05 | 0.000220291 | 0.000495376 | 0.002137102 |
| GTEX-XQ8I- | 4.99713E-05 | 2.82604E-05 | 0.00010347 | 4.4533E-05 | 0.00166338 | 0.000141232 | 0.000168845 | 0.000758218 | 0.000867759 | 0.002604303 |
| GTEX-XUJ4- | 6.74805E-05 | 0 | 6.14285E-05 | 0.000150874 | 0.000560117 | 0.000234365 | 9.85928E-05 | 0.000255552 | 0.000579979 | 0.001595406 |
| GTEX-XUW1- | 8.38409E-05 | 1.00355E-05 | 7.17081E-05 | 5.01452E-05 | 5.67826E-05 | 0.000265654 | 0.000178316 | 0.000307994 | 0.000853092 | 0.00372245 |
| GTEX-XUYS- | 7.60858E-05 | 1.73329E-05 | 8.91856E-05 | 0.000377743 | 7.76935E-05 | 0.000162381 | 0.00011728 | 0.000220664 | 0.000685308 | 0.005556789 |
| GTEX-XUZC- | 5.49552E-05 | 1.2652E-05 | 0.000289681 | 0.000100334 | 0.000961301 | 0.000285699 | 0.000121277 | 0.000324114 | 0.000716777 | 0.001956516 |
| GTEX-XV7Q- | 0.000128413 | 4.35948E-05 | 9.78108E-05 | 0.000263583 | 0.000140705 | 0.000194173 | 0.000348379 | 0.0010052 | 0.001629057 | 0.005579846 |
| GTEX-XYKS- | 0.000102153 | 4.51433E-05 | 0.000186557 | 0.000239043 | 9.13815E-05 | 0.00030221 | 0.000380296 | 0.000599201 | 0.001893993 | 0.008212558 |
| GTEX-Y111- | 6.23287E-05 | 0 | 2.68095E-05 | 3.34127E-05 | 0.00013244 | 0.000101762 | 0.000127072 | 0.000296622 | 0.000932753 | 0.004651603 |
| GTEX-Y114- | 6.57164E-05 | 3.77254E-05 | 0.000251889 | 0.000118262 | 0.000699081 | 0.000181429 | 9.11415E-05 | 0.000246981 | 0.000640136 | 0.00289372 |
| GTEX-Y3IK- | 7.99038E-05 | 4.01722E-05 | 3.93193E-05 | 0.000287507 | 0.000143892 | 7.04822E-05 | 0.000110064 | 0.000291757 | 0.000624685 | 0.002342775 |
| GTEX-Y5LM- | 8.45199E-05 | 1.07875E-05 | 3.33027E-05 | 6.7732E-05 | 0.000111751 | 0.000344603 | 0.000498029 | 0.000280557 | 0.000688802 | 0.003357082 |
| GTEX-Y5V5- | 0.00014596 | 4.68739E-05 | 8.47172E-05 | 9.41624E-05 | 0.000688687 | 0.000542716 | 0.000782765 | 0.000432597 | 0.000879033 | 0.01357353 |
| GTEX-Y5V6- | 4.45419E-05 | 6.32804E-06 | 3.66574E-05 | 6.72602E-05 | 0.000469388 | 0.000267073 | 0.000111778 | 0.000200611 | 0.000467407 | 0.001305888 |
| GTEX-Y8DK- | 7.85669E-05 | 1.54204E-05 | 5.46024E-05 | 3.64615E-05 | 0.000133656 | 0.000148502 | 0.000108441 | 0.000212509 | 0.000597465 | 0.002949368 |
| GTEX-Y8E5- | 0.000259747 | 0.000111331 | 0.000263446 | 0.000501265 | 5.35836E-05 | 0.000175121 | 0.000327931 | 0.0003467 | 0.001697552 | 0.005939533 |
| GTEX-Y8LW- | 7.82489E-05 | 3.30705E-05 | 6.08193E-05 | 0.000178874 | 0.000169762 | 0.000285092 | 0.000195065 | 0.000291501 | 0.001008784 | 0.004461649 |
| GTEX-Y9LG- | 6.37375E-05 | 1.53667E-05 | 7.48166E-05 | 9.84447E-05 | 0.000540452 | 0.000127371 | 6.02879E-05 | 0.000196553 | 0.000400112 | 0.001161164 |
| GTEX-YB5E- | 9.86905E-05 | 1.31139E-05 | 0.000381083 | 0.000151993 | 0.00113532 | 0.000527203 | 0.000333468 | 0.000319248 | 0.000627749 | 0.002764344 |
| GTEX-YB5K- | 3.74572E-05 | 5.48238E-06 | 1.81574E-05 | 3.39025E-05 | 0.00019581 | 0.000209998 | 8.22854E-05 | 0.000219726 | 0.000601151 | 0.001830504 |
| GTEX-YBZK- | 0.000182218 | 0.000171782 | 0.000253021 | 0.000735435 | 0.000195003 | 0.000477104 | 0.000346674 | 0.000498091 | 0.001362322 | 0.015056811 |
| GTEX-YEC3- | 4.03569E-05 | 2.94987E-06 | 5.30163E-05 | 3.6942E-05 | 0.000166827 | 0.000170535 | 5.20575E-05 | 0.000153576 | 0.000462158 | 0.000992024 |
| GTEX-YEC4- | 7.80527E-05 | 0 | 6.21269E-05 | 6.64998E-05 | 0.000302638 | 0.000306971 | 0.000280348 | 0.000275836 | 0.000836397 | 0.00358754 |
| GTEX-YECK- | 7.82638E-05 | 1.72371E-05 | 4.13939E-05 | 5.3597E-05 | 0.000165443 | 9.77161E-05 | 7.2275E-05 | 0.000229835 | 0.000669166 | 0.003662662 |
| GTEX-YFC4- | 7.07436E-05 | 1.14573E-05 | 2.60606E-05 | 2.37874E-05 | 5.76935E-05 | 9.59495E-05 | 0.000111286 | 0.000308557 | 0.000648391 | 0.002380167 |
| GTEX-YFCO- | 6.63462E-05 | 1.03298E-05 | 0.000110976 | 8.77855E-05 | 0.001605061 | 0.000215358 | 0.000139775 | 0.000244499 | 0.000619575 | 0.001898475 |
| GTEX-YJ8O- | 6.35683E-05 | 1.80086E-05 | 1.85558E-05 | 4.68764E-05 | 8.56236E-05 | 9.40976E-05 | 7.97731E-05 | 0.000175117 | 0.000472818 | 0.002480466 |
| GTEX-Z93S- | 6.43045E-05 | 2.12137E-05 | 9.27833E-05 | 7.54923E-05 | 0.00012933 | 0.00012504 | 8.77784E-05 | 0.000248322 | 0.000816331 | 0.002949411 |
| GTEX-Z9EW- | 3.63637E-05 | 7.45249E-06 | 3.22586E-05 | 5.43915E-05 | 0.000350075 | 0.000251097 | 0.000114575 | 0.000231187 | 0.000607927 | 0.001928632 |
| GTEX-ZA64- | 4.98557E-05 | 1.11198E-05 | 5.26169E-05 | 8.27167E-05 | 0.000797719 | 0.000234107 | 0.000118088 | 0.000230327 | 0.000487837 | 0.00202386 |
| GTEX-ZAB4- | 0.000112039 | 2.13641E-05 | 0.000112196 | 0.000137916 | 0.000259786 | 0.000346397 | 0.000170887 | 0.000236772 | 0.000829361 | 0.004726292 |
| GTEX-ZAB5- | 0.000109311 | 7.04998E-06 | 7.08391E-05 | 8.17804E-05 | 0.000719435 | 0.000302064 | 0.000238414 | 0.000287213 | 0.000614993 | 0.0035762 |
| GTEX-ZAJG- | 7.27478E-05 | 1.615E-05 | 4.81454E-05 | 4.00861E-05 | 0.00073593 | 9.79141E-05 | 0.000147834 | 0.000458888 | 0.000604712 | 0.001899006 |
| GTEX-ZAK1- | 8.21656E-05 | 0 | 4.39074E-05 | 4.17974E-05 | 6.8782E-05 | 0.000124283 | 0.000138483 | 0.000312901 | 0.000796031 | 0.002886053 |
| GTEX-ZAKK- | 0.000154301 | 3.13285E-05 | 0.001027427 | 0.000424433 | 0.00013093 | 0.000606775 | 0.000331732 | 0.000555343 | 0.001182239 | 0.010121288 |
| GTEX-ZC5H- | 0.000119999 | 5.07213E-05 | 3.9295E-05 | 0.000193462 | 3.70992E-05 | 0.000163888 | 0.000422678 | 0.000714941 | 0.001059579 | 0.003597191 |
| GTEX-ZDTS- | 7.12315E-05 | 1.39866E-05 | 2.07988E-05 | 5.76379E-05 | 0.000283626 | 0.000112865 | 9.91121E-05 | 0.000203329 | 0.000639947 | 0.003483033 |
| GTEX-ZDXO- | 9.57234E-05 | 3.86807E-05 | 5.71538E-05 | 0.000127416 | 8.79264E-05 | 0.000340744 | 0.000502502 | 0.000340525 | 0.000780135 | 0.019407275 |
| GTEX-ZDYS- | 6.7209E-05 | 2.64361E-05 | 5.15312E-05 | 3.96431E-05 | 0.000276248 | 0.000300751 | 0.000176803 | 0.000225437 | 0.000835246 | 0.006882641 |
| GTEX-ZE7O- | 0.000160079 | 3.85514E-05 | 8.12554E-05 | 0.000270056 | 1.76142E-05 | 0.00015095 | 0.000240425 | 0.000302939 | 0.000538133 | 0.009308463 |
| GTEX-ZF28- | 0.000108441 | 3.4653E-05 | 9.66415E-05 | 0.000109277 | 0.001267308 | 0.000225491 | 0.000414047 | 0.000567228 | 0.001420855 | 0.007844366 |
| GTEX-ZF2S- | 0.000103641 | 2.41253E-05 | 4.70107E-05 | 0.000206742 | 0.000276829 | 0.00024729 | 0.000239528 | 0.000332434 | 0.000881739 | 0.004113516 |
| GTEX-ZF3C- | 9.51631E-05 | 1.19336E-05 | 5.27216E-05 | 7.51653E-05 | 0.000268127 | 9.91282E-05 | 0.000200933 | 0.00034237 | 0.00092077 | 0.002563304 |
| GTEX-ZLV1- | 7.00325E-05 | 6.99131E-06 | 9.07752E-05 | 7.44489E-05 | 0.000711544 | 0.000570539 | 0.000192573 | 0.000445535 | 0.000879309 | 0.002906511 |
| GTEX-ZP4G- | 0.002422279 | 1.31887E-05 | 0.000116746 | 0.002521311 | 0.000392515 | 0.00039921 | 0.000843996 | 0.000393553 | 0.000561684 | 0.002435571 |
| GTEX-ZPCL- | 9.82697E-05 | 0 | 4.86183E-05 | 0.00010209 | 0.000666283 | 0.000379156 | 0.000131875 | 0.000195625 | 0.000483986 | 0.002290767 |
| GTEX-ZQUD- | 5.60141E-05 | 1.00725E-05 | 4.65888E-05 | 8.03336E-05 | 0.000810002 | 0.000113917 | 0.000126147 | 0.000203124 | 0.000414237 | 0.001269631 |
| GTEX-ZT9X- | 6.91277E-05 | 1.58926E-05 | 2.43578E-05 | 9.73118E-05 | 0.000292936 | 0.000181775 | 0.00018815 | 0.000538538 | 0.000960121 | 0.002285202 |
| GTEX-ZTTD- | 0.000601781 | 5.97777E-05 | 0.001073202 | 0.003344247 | 7.39845E-05 | 0.00554128 | 0.001004376 | 0.001494991 | 0.003531069 | 0.034738401 |
| GTEX-ZTX8- | 4.20591E-05 | 0 | 4.82065E-05 | 5.10026E-05 | 0.000533686 | 0.000110775 | 8.58424E-05 | 0.000199077 | 0.000547257 | 0.001340878 |
| GTEX-ZUA1- | 0.00010074 | 2.21618E-05 | 2.57594E-05 | 3.68796E-05 | 8.12147E-05 | 0.000138712 | 0.000125676 | 0.000266627 | 0.000804796 | 0.003696047 |
| GTEX-ZV68- | 5.22371E-05 | 5.95867E-06 | 3.30144E-05 | 3.7922E-05 | 0.000429947 | 7.20477E-05 | 6.2631E-05 | 0.000271266 | 0.000480095 | 0.001275654 |
| GTEX-ZV6S- | 8.57149E-05 | 1.81894E-05 | 6.9126E-05 | 8.06787E-05 | 0.000769781 | 0.00018317 | 0.000121678 | 0.000316069 | 0.000864871 | 0.002384453 |
| GTEX-ZV7C- | 5.0302E-05 | 1.46576E-05 | 0.00012673 | 6.68318E-05 | 0.001270945 | 0.000160332 | 0.000143612 | 0.000373258 | 0.000659633 | 0.002312171 |
| GTEX-ZVE1- | 0.00012878 | 1.70381E-05 | 9.13759E-05 | 0.000180692 | 0.001243887 | 0.000133581 | 0.000308976 | 0.000676625 | 0.000893197 | 0.003904928 |
| GTEX-ZVP2- | 6.16194E-05 | 1.7448E-05 | 0.000151112 | 0.000237535 | 0.00215334 | 0.000193685 | 8.32248E-05 | 0.000282174 | 0.000652767 | 0.001945379 |
| GTEX-ZVT2- | 4.02993E-05 | 0 | 0.000103401 | 5.31815E-05 | 0.000450066 | 0.000362743 | 8.24593E-05 | 0.000253002 | 0.00044898 | 0.001883346 |
| GTEX-ZVT4- | 5.9136E-05 | 0 | 2.30095E-05 | 8.72466E-05 | 0.00016253 | 0.000184693 | 0.000119092 | 0.000274458 | 0.000642332 | 0.004542687 |
| GTEX-ZVTK- | 7.93854E-05 | 4.18984E-06 | 2.28741E-05 | 0.010035183 | 7.22687E-05 | 0.000113843 | 8.08874E-05 | 0.000209594 | 0.000694028 | 0.001482687 |
| GTEX-ZVZO- | 0.00013727 | 8.71821E-06 | 0.000161554 | 0.000226042 | 0.000380368 | 0.000250425 | 0.000225818 | 0.000396701 | 0.001465386 | 0.010188215 |
| GTEX-ZVZP- | 4.32624E-05 | 1.00028E-05 | 0.000123173 | 0.000181959 | 0.000126338 | 0.000328887 | 0.000119926 | 0.000342916 | 0.000543496 | 0.002391944 |
| GTEX-ZWKS- | 0.000155935 | 1.13865E-05 | 0.00027044 | 7.34304E-05 | 0.0003211 | 0.000176827 | 0.000874301 | 0.000607099 | 0.000891631 | 0.003800053 |
| GTEX-ZXG5- | 0.00010376 | 7.16886E-05 | 5.96241E-05 | 4.10611E-05 | 0.000893866 | 0.000191197 | 0.000244076 | 0.00034392 | 0.000657002 | 0.00410139 |
| GTEX-ZYFC- | 8.56266E-05 | 1.21478E-05 | 4.31301E-05 | 6.64223E-05 | 5.01945E-05 | 0.000140962 | 8.93779E-05 | 0.000189098 | 0.000715936 | 0.002960021 |
| GTEX-ZYFD- | 2.94072E-05 | 8.57476E-06 | 1.12634E-05 | 3.36946E-05 | 3.02291E-05 | 7.30634E-05 | 0.000101509 | 0.000257375 | 0.000485184 | 0.001909849 |
| GTEX-ZYT6- | 3.67449E-05 | 2.8591E-06 | 6.85261E-06 | 2.20031E-05 | 0.000128469 | 9.29903E-05 | 5.79668E-05 | 0.000155503 | 0.000492705 | 0.001614678 |
| GTEX-ZYVF- | 8.48959E-05 | 6.31043E-05 | 4.41736E-05 | 0.000207585 | 3.89498E-05 | 0.000210201 | 0.00043451 | 0.00056836 | 0.00168698 | 0.007882596 |
| GTEX-ZYW4- | 8.08685E-05 | 1.7121E-05 | 2.65575E-05 | 4.28254E-05 | 0.000199042 | 9.63007E-05 | 0.000197891 | 0.000344058 | 0.000978231 | 0.007093841 |
| GTEX-ZYWO- | 5.60158E-05 | 6.52075E-06 | 0.000100844 | 0.000144681 | 0.000951346 | 0.000173667 | 0.000131244 | 0.000334419 | 0.000791496 | 0.002016117 |
| GTEX-ZYY3- | 6.67358E-05 | 2.79027E-05 | 0.000364048 | 0.000725544 | 0.000191053 | 0.000152331 | 0.000185371 | 0.00030456 | 0.001034612 | 0.002309426 |
| GTEX-ZZ64- | 6.60827E-05 | 1.7656E-05 | 3.66681E-05 | 5.37292E-05 | 0.000150459 | 0.000296102 | 0.000193567 | 0.000246934 | 0.001037303 | 0.006212462 |
| GTEX-ZZPT- | 0.000222133 | 2.34928E-05 | 0.000123222 | 0.000130198 | 1.36869E-05 | 0.000393103 | 0.000424584 | 0.000955258 | 0.001951582 | 0.006394722 |
| GTEX-ZZPU- | 4.4381E-05 | 2.54546E-05 | 7.20849E-05 | 0.000111455 | 0.000326786 | 0.000460191 | 0.000261032 | 0.000272845 | 0.000678311 | 0.003593867 |
