# Supplementary material for: The prognostic value of ubiquitin/ubiquitin-like-related genes along with immune cell infiltration and clinicopathological features in osteosarcoma
Source: J Orthop Surg Res. 2024 Jun 15;19:356. doi: 10.1186/s13018-024-04781-1 (PMC11179372; doi:10.1186/s13018-024-04781-1)
Supplement: Supplementary file 3 [file 13018_2024_4781_MOESM3_ESM.docx]

**Table S3 TME estimated by MCPcounter in GSE21257 cohort**

| Sample-ID | T cells | Cytotoxic lymphocytes | B lineage | NK cells | Monocytic lineage | Myeloid dendritic cells | Neutrophils | Endothelial cells | Fibroblasts |
| --- | --- | --- | --- | --- | --- | --- | --- | --- | --- |
| GSM530667 | 0.07112513 | 0.02497644 | 0.023953909 | 0.019222276 | 0.387337064 | 0.072830027 | 0.067129975 | 0.149625261 | 0.867052545 |
| GSM530899 | 0.055799205 | 0.029656675 | 0.015628469 | 0.008445358 | 0.342471538 | 0.024634296 | 0.075787144 | 0.121389033 | 0.807852895 |
| GSM531283 | 0.051534564 | 0.013146248 | 0.021796468 | 0.008920484 | 0.255041109 | 0.035522423 | 0.07564445 | 0.182792566 | 0.73774423 |
| GSM531284 | 0.050035486 | 0.01622078 | 0.018627627 | 0.007844667 | 0.293310023 | 0.05301597 | 0.065141434 | 0.115392899 | 0.917679483 |
| GSM531285 | 0.070131645 | 0.064992243 | 0.035969963 | 0.056476852 | 0.282121581 | 0.023680691 | 0.053585971 | 0.14177894 | 0.55981515 |
| GSM531286 | 0.081526233 | 0.019455992 | 0.027828008 | 0.001389386 | 0.162939872 | 0.021289489 | 0.068490525 | 0.187216059 | 0.842544309 |
| GSM531287 | 0.096873068 | 0.048728938 | 0.0244972 | 0 | 0.342200477 | 0.036911899 | 0.063439597 | 0.202238966 | 0.772519482 |
| GSM531288 | 0.086989745 | 0.05840059 | 0.024830304 | 0.010717128 | 0.329058554 | 0.04458797 | 0.076120918 | 0.262422942 | 0.96669308 |
| GSM531289 | 0.048844909 | 0.0018065 | 0.040455865 | 0.007643896 | 0.247242568 | 0.029523785 | 0.061525438 | 0.163001509 | 0.775375692 |
| GSM531290 | 0.039892824 | 0.011958418 | 0.02634873 | 0.026646101 | 0.227697521 | 0.024147991 | 0.058321758 | 0.139924466 | 0.486874729 |
| GSM531291 | 0.114223022 | 0.132857738 | 0.018690157 | 0.050959538 | 0.326779936 | 0.046365959 | 0.055584904 | 0.228894731 | 0.726565243 |
| GSM531292 | 0.073209397 | 0.042460994 | 0.025524202 | 0.031123871 | 0.363762822 | 0.052568949 | 0.068155762 | 0.161410149 | 0.682802139 |
| GSM531293 | 0.091432899 | 0.047338351 | 0.017262424 | 0.015301887 | 0.327849142 | 0.084704475 | 0.063150788 | 0.235878536 | 0.743144929 |
| GSM531294 | 0.061601825 | 0.02305077 | 0.019045884 | 0.001866181 | 0.273074759 | 0.02590786 | 0.063775481 | 0.182493089 | 0.64007959 |
| GSM531295 | 0.066642421 | 0.036386249 | 0.01116316 | 0.009027588 | 0.24418759 | 0.02912767 | 0.055200123 | 0.174837986 | 0.755028697 |
| GSM531296 | 0.073124064 | 0.024877634 | 0.023805081 | 0.025815423 | 0.393268409 | 0.043580826 | 0.069042341 | 0.160486743 | 0.791458611 |
| GSM531297 | 0.060586154 | 0.028518563 | 0.02360328 | 0.005707331 | 0.281575303 | 0.03682861 | 0.068359856 | 0.16534667 | 1 |
| GSM531298 | 0.07191402 | 0.034025042 | 0.018612608 | 0.003343337 | 0.135083559 | 0.033864501 | 0.10868102 | 0.208794819 | 0.738389125 |
| GSM531299 | 0.062114148 | 0.003815003 | 0.011359074 | 0.007269132 | 0.26360635 | 0.01504686 | 0.074102297 | 0.196667546 | 0.806916176 |
| GSM531300 | 0.036059547 | 0.010719152 | 0.008980246 | 0.009351536 | 0.302243685 | 0.010058941 | 0.080257802 | 0.170346663 | 0.721439977 |
| GSM531301 | 0.059282905 | 0.03735001 | 0.021780245 | 0.009132809 | 0.301876 | 0.048495342 | 0.065740504 | 0.222724806 | 0.769598461 |
| GSM531302 | 0.175655062 | 0.175619331 | 0.018908134 | 0.052921692 | 0.389979013 | 0.279308776 | 0.06133427 | 0.225023855 | 0.64437895 |
| GSM531303 | 0.086037832 | 0.141110166 | 0.020572697 | 0.048880706 | 0.525146159 | 0.213566245 | 0.081823759 | 0.121071715 | 0.766711038 |
| GSM531304 | 0.143211812 | 0.192380739 | 0.019905742 | 0.057806949 | 0.384139718 | 0.010991872 | 0.081719921 | 0.177931924 | 0.841184184 |
| GSM531305 | 0.143738133 | 0.163458269 | 0.036236642 | 0.064085135 | 0.395069379 | 0.08002607 | 0.083485946 | 0.158728704 | 0.720129072 |
| GSM531306 | 0.047388327 | 0.054269338 | 0.020121052 | 0.01590921 | 0.325429053 | 0.02726552 | 0.06938821 | 0.14316609 | 0.510286438 |
| GSM531307 | 0.113598717 | 0.074637724 | 0.032860018 | 0.019611131 | 0.37937067 | 0.090764122 | 0.078633447 | 0.161927693 | 0.872536926 |
| GSM531308 | 0.044246757 | 0.039526335 | 0.039534996 | 0.006877981 | 0.347605186 | 0.037424669 | 0.171418792 | 0.132022831 | 0.691354859 |
| GSM531309 | 0.152390223 | 0.156111765 | 0.055343255 | 0.05233686 | 0.401400623 | 0.039781764 | 0.153181159 | 0.205846648 | 0.752742173 |
| GSM531310 | 0.144113546 | 0.109713251 | 0.031376141 | 0.027098154 | 0.4422727 | 0.055267864 | 0.076943827 | 0.155289519 | 0.687512721 |
| GSM531311 | 0.054124475 | 0.029991092 | 0.019330997 | 0.00383941 | 0.447272035 | 0.044298834 | 0.088718472 | 0.126909 | 0.833800999 |
| GSM531312 | 0.057845293 | 0.066822423 | 0.019449735 | 0.007832413 | 0.291905464 | 0.044945512 | 0.098326917 | 0.13490055 | 0.646033552 |
| GSM531313 | 0.141092963 | 0.181082008 | 0.119499048 | 0.034560903 | 0.374144314 | 0.041606796 | 0.08490638 | 0.126233743 | 0.726059244 |
| GSM531314 | 0.091987745 | 0.062417492 | 0.031855207 | 0.021405455 | 0.372762993 | 0.044044106 | 0.177696443 | 0.175822951 | 0.834486745 |
| GSM531319 | 0.06552588 | 0.048679019 | 0.016629303 | 0.00983068 | 0.346884456 | 0.060412873 | 0.080410061 | 0.164687105 | 0.674955119 |
| GSM531320 | 0.069158683 | 0.015203896 | 0.047512931 | 0.005459123 | 0.378357307 | 0.027100072 | 0.109439393 | 0.159260553 | 0.815378476 |
| GSM531321 | 0.14450684 | 0.091156844 | 0.029786678 | 0.015367489 | 0.512755708 | 0.118589755 | 0.084570734 | 0.173270578 | 0.803980526 |
| GSM531322 | 0.089354953 | 0.073922672 | 0.014158934 | 0.023266483 | 0.354329683 | 0.063040793 | 0.090683886 | 0.153159729 | 0.604151454 |
| GSM531323 | 0.105089727 | 0.047539306 | 0.026727412 | 0.026271721 | 0.392970511 | 0.040490181 | 0.127938294 | 0.162675214 | 0.826456401 |
| GSM531324 | 0.170120859 | 0.204259672 | 0.027129814 | 0.029951796 | 0.377807443 | 0.050602473 | 0.098742416 | 0.223748174 | 0.728237469 |
| GSM531325 | 0.066642135 | 0.019422203 | 0.011528063 | 0.000627744 | 0.325969001 | 0.024449629 | 0.098747584 | 0.170799982 | 0.831584071 |
| GSM531326 | 0.119911232 | 0.063201735 | 0.02482555 | 0.017817841 | 0.449921672 | 0.03501626 | 0.087625096 | 0.195054904 | 0.615284751 |
| GSM531327 | 0.054189618 | 0.010221265 | 0.010032765 | 0.00575673 | 0.265735136 | 0.025640131 | 0.039665434 | 0.16434241 | 0.402553354 |
| GSM531328 | 0.086502827 | 0.093616946 | 0.029954224 | 0.009592264 | 0.338794807 | 0.035927946 | 0.115793534 | 0.180833082 | 0.841068493 |
| GSM531329 | 0.06588067 | 0.023847296 | 0.024805579 | 0.004632146 | 0.130877179 | 0.02440787 | 0.086365028 | 0.094291069 | 0.586878256 |
| GSM531330 | 0.071264169 | 0.102893374 | 0.023308757 | 0.036065358 | 0.440599773 | 0.018816234 | 0.084061516 | 0.153988444 | 0.75823081 |
| GSM531331 | 0.079013114 | 0.057641299 | 0.031548193 | 0.010155371 | 0.282001109 | 0.031704061 | 0.086948471 | 0.129942217 | 0.773198238 |
| GSM531332 | 0.116803592 | 0.063281524 | 0.019248186 | 0.017075932 | 0.34399111 | 0.041931431 | 0.146842923 | 0.233557343 | 0.875513319 |
| GSM531333 | 0.089325098 | 0.109481515 | 0.037167555 | 0.076029059 | 0.429069242 | 0.034508075 | 0.084627349 | 0.150135379 | 0.812240829 |
| GSM531334 | 0.09139584 | 0.040059426 | 0.026307661 | 0.013110477 | 0.305578322 | 0.094575769 | 0.100538629 | 0.136171806 | 0.693819684 |
| GSM531335 | 0.102710554 | 0.094561126 | 0.019590564 | 0.055057542 | 0.374412535 | 0.02362759 | 0.138281815 | 0.195843253 | 0.715145163 |
| GSM531351 | 0.05015677 | 0.024665523 | 0.027767261 | 0.012002314 | 0.363362538 | 0.045924369 | 0.104026575 | 0.136066999 | 0.680268474 |
| GSM531352 | 0.070250633 | 0.033986378 | 0.027590702 | 0.006754688 | 0.356818894 | 0.032322328 | 0.069438353 | 0.182689879 | 0.644808736 |
