# Supplementary material for: The prognostic value of ubiquitin/ubiquitin-like-related genes along with immune cell infiltration and clinicopathological features in osteosarcoma
Source: J Orthop Surg Res. 2024 Jun 15;19:356. doi: 10.1186/s13018-024-04781-1 (PMC11179372; doi:10.1186/s13018-024-04781-1)
Supplement: Supplementary file 4 [file 13018_2024_4781_MOESM4_ESM.docx]

**Table S4 DEGs between high and low gene significance score groups**

| gene_name | baseMean | log2FoldChange | lfcSE | stat | pvalue | padj |
| --- | --- | --- | --- | --- | --- | --- |
| TRIM8 | 5232.485529 | 1.2917994 | 0.116776152 | 11.06218499 | 1.91378E-28 | 3.77837E-24 |
| RP13-487C10.1 | 12.01425495 | 3.456186711 | 0.417262567 | 8.283002081 | 1.20108E-16 | 1.18565E-12 |
| RP11-47A8.5 | 572.4017101 | 1.545288417 | 0.187850166 | 8.22617542 | 1.93286E-16 | 1.27201E-12 |
| COMP | 2881.964532 | -3.552543704 | 0.466059783 | -7.622506453 | 2.48797E-14 | 1.228E-10 |
| OXTR | 249.5260175 | -2.468821862 | 0.328699134 | -7.510886423 | 5.87283E-14 | 2.31895E-10 |
| RGS4 | 419.2991072 | -2.808320448 | 0.40061571 | -7.010010788 | 2.383E-12 | 7.84125E-09 |
| DDIT4L | 1424.441006 | -3.248314066 | 0.473299435 | -6.863126868 | 6.73693E-12 | 1.47786E-08 |
| MEG3 | 1265.606594 | -3.078189133 | 0.448096431 | -6.869479241 | 6.44367E-12 | 1.47786E-08 |
| DKK1 | 1095.868032 | 2.780604867 | 0.415341682 | 6.69474071 | 2.16054E-11 | 4.26556E-08 |
| COL8A1 | 3165.992972 | -2.101578026 | 0.314824625 | -6.675392775 | 2.46571E-11 | 4.4255E-08 |
| OPN3 | 1788.104108 | 1.982026836 | 0.304771435 | 6.503322187 | 7.85653E-11 | 1.2926E-07 |
| PRR15 | 61.54933664 | 2.808962379 | 0.436755949 | 6.431423286 | 1.26415E-10 | 1.91985E-07 |
| SCRG1 | 342.9594102 | -3.030104677 | 0.477981516 | -6.339376268 | 2.30697E-10 | 3.25332E-07 |
| ACTG2 | 875.2164353 | -2.915557262 | 0.464223988 | -6.280496773 | 3.37493E-10 | 4.09248E-07 |
| ISM1 | 506.98741 | 2.189675417 | 0.349020189 | 6.273778663 | 3.52389E-10 | 4.09248E-07 |
| RP11-81A22.5 | 256.986946 | 2.651731696 | 0.421806212 | 6.286611294 | 3.2447E-10 | 4.09248E-07 |
| C1QTNF3 | 2242.162941 | -2.970438922 | 0.475243209 | -6.25035533 | 4.0952E-10 | 4.25534E-07 |
| MICAL2 | 3670.942629 | -1.431091014 | 0.228885337 | -6.252436404 | 4.04098E-10 | 4.25534E-07 |
| RAB11FIP1 | 1055.304823 | 1.568946215 | 0.254751792 | 6.158724946 | 7.3333E-10 | 7.23906E-07 |
| CYFIP2 | 2871.064107 | 2.185186776 | 0.355622539 | 6.144680207 | 8.01248E-10 | 7.53287E-07 |
| PTN | 11494.21479 | -2.192629457 | 0.358421787 | -6.117455842 | 9.50811E-10 | 8.53266E-07 |
| SFXN2 | 333.6886376 | 1.085368403 | 0.178340122 | 6.085946278 | 1.15805E-09 | 9.94062E-07 |
| HTR2A | 87.3250008 | -2.158326293 | 0.358653981 | -6.017851202 | 1.76748E-09 | 1.39581E-06 |
| FGFBP3 | 142.6248505 | 1.242872641 | 0.209425126 | 5.934687312 | 2.94406E-09 | 2.23556E-06 |
| MTND1P23 | 1565.121819 | -2.059441966 | 0.34843361 | -5.910572086 | 3.40922E-09 | 2.49289E-06 |
| CDK6 | 2839.81294 | -1.558328203 | 0.265482528 | -5.869795706 | 4.36332E-09 | 3.07661E-06 |
| CACNA1E | 170.2722752 | 2.386110488 | 0.408120078 | 5.846589319 | 5.01754E-09 | 3.09617E-06 |
| FGGY | 446.6534378 | -1.366886035 | 0.233157896 | -5.862490866 | 4.55975E-09 | 3.09617E-06 |
| MAN1A1 | 1327.842094 | -1.333800553 | 0.227879996 | -5.853083095 | 4.82543E-09 | 3.09617E-06 |
| PRTG | 676.5673553 | 1.499529791 | 0.256480603 | 5.846562166 | 5.01836E-09 | 3.09617E-06 |
| NPY1R | 97.66533168 | 3.145990975 | 0.538627229 | 5.8407574 | 5.1964E-09 | 3.10887E-06 |
| ANGPTL2 | 11162.18345 | -1.684721672 | 0.289338537 | -5.82266604 | 5.79162E-09 | 3.36306E-06 |
| CACNB4 | 143.177223 | 1.90010567 | 0.327411289 | 5.803421365 | 6.49752E-09 | 3.46704E-06 |
| FAM166B | 232.3706073 | 2.933135524 | 0.505008109 | 5.808095898 | 6.31873E-09 | 3.46704E-06 |
| OGN | 3651.726825 | -2.715336384 | 0.467883774 | -5.803442086 | 6.49672E-09 | 3.46704E-06 |
| CTD-2541J13.1 | 32.72113158 | -1.988941547 | 0.343249283 | -5.794452159 | 6.85446E-09 | 3.56125E-06 |
| C1QTNF7 | 302.4719862 | -2.522517676 | 0.436476722 | -5.779271947 | 7.50246E-09 | 3.64852E-06 |
| CALD1 | 14205.44143 | -1.175402723 | 0.203440926 | -5.777611948 | 7.57683E-09 | 3.64852E-06 |
| TAGLN | 3045.727192 | -1.618429814 | 0.279868557 | -5.782821161 | 7.34581E-09 | 3.64852E-06 |
| DBNDD2 | 366.1606718 | 1.326939839 | 0.230045458 | 5.768163612 | 8.014E-09 | 3.76715E-06 |
| GREM1 | 3349.746587 | -2.275469417 | 0.3957426 | -5.74987231 | 8.93109E-09 | 4.10062E-06 |
| TSPAN5 | 923.2931506 | -1.424623586 | 0.249089734 | -5.719318748 | 1.06952E-08 | 4.79898E-06 |
| BMP2 | 1641.130422 | 1.402103488 | 0.245675476 | 5.707136551 | 1.14893E-08 | 4.93114E-06 |
| GNG4 | 1123.053413 | 2.207694005 | 0.386627769 | 5.71012789 | 1.12891E-08 | 4.93114E-06 |
| BNC2 | 1064.770031 | -1.671417258 | 0.293615952 | -5.69252879 | 1.25171E-08 | 5.258E-06 |
| ACAN | 19156.39935 | -2.365443404 | 0.417484744 | -5.665939738 | 1.46221E-08 | 5.8946E-06 |
| SAMD5 | 882.4459483 | -1.769629168 | 0.312332528 | -5.665849726 | 1.46298E-08 | 5.8946E-06 |
| CHI3L1 | 151.4519352 | -2.508351683 | 0.444306228 | -5.645546982 | 1.64657E-08 | 6.50164E-06 |
| MFAP5 | 152.0319458 | -2.524267726 | 0.447488045 | -5.640972431 | 1.69092E-08 | 6.54587E-06 |
| GADD45B | 2627.029684 | 1.122534262 | 0.199527724 | 5.625956342 | 1.84483E-08 | 7.00432E-06 |
| CDS1 | 80.61213519 | -1.92272482 | 0.342997986 | -5.60564463 | 2.07481E-08 | 7.72887E-06 |
| FOSB | 1404.158281 | 2.352502794 | 0.42055114 | 5.593856661 | 2.2208E-08 | 8.11806E-06 |
| ITK | 547.855695 | 2.187699152 | 0.39131024 | 5.590702536 | 2.26153E-08 | 8.11806E-06 |
| RUNDC3A | 21.95871105 | 1.498771488 | 0.269798693 | 5.555147334 | 2.77378E-08 | 9.77905E-06 |
| PRICKLE2 | 727.1890472 | -1.519703029 | 0.274131981 | -5.543691112 | 2.96161E-08 | 1.0094E-05 |
| NRXN1 | 682.0232446 | 2.608431251 | 0.473079704 | 5.513724707 | 3.51318E-08 | 1.15601E-05 |
| TNR | 392.3520569 | 3.344760101 | 0.607700396 | 5.503962349 | 3.71349E-08 | 1.20189E-05 |
| CRISPLD1 | 2956.921449 | -1.50693048 | 0.275002166 | -5.479704038 | 4.26038E-08 | 1.33512E-05 |
| RP11-45P15.4 | 44.47359563 | -1.263942317 | 0.230612949 | -5.480795084 | 4.23419E-08 | 1.33512E-05 |
| ABLIM1 | 1541.350827 | 1.141456348 | 0.208864195 | 5.465064754 | 4.62738E-08 | 1.40896E-05 |
| FLNC | 5397.439707 | -2.369928247 | 0.433685009 | -5.464630316 | 4.63873E-08 | 1.40896E-05 |
| LSP1 | 5362.614453 | -1.255470798 | 0.229986776 | -5.458882552 | 4.79141E-08 | 1.43328E-05 |
| GRHL1 | 75.63593478 | 1.295409421 | 0.238125899 | 5.440019033 | 5.32749E-08 | 1.52906E-05 |
| PLEKHH1 | 168.5023158 | 1.481488217 | 0.272358912 | 5.439470307 | 5.34392E-08 | 1.52906E-05 |
| RP11-304F15.3 | 64.85617166 | 1.752845287 | 0.321976634 | 5.444013949 | 5.20931E-08 | 1.52906E-05 |
| RP1-80N2.3 | 16.80742419 | 2.175933338 | 0.400627305 | 5.431315615 | 5.59401E-08 | 1.55553E-05 |
| MEG9 | 16.95553026 | -2.810958988 | 0.520076588 | -5.404894307 | 6.48466E-08 | 1.74195E-05 |
| SLC16A8 | 244.7966036 | 1.894662624 | 0.350625199 | 5.403669304 | 6.52913E-08 | 1.74195E-05 |
| GNG12 | 3345.038557 | -1.176391553 | 0.217809259 | -5.401017193 | 6.62641E-08 | 1.74434E-05 |
| TSPAN18 | 4266.927023 | -1.311199064 | 0.244048779 | -5.372692583 | 7.75695E-08 | 2.01507E-05 |
| IL17RD | 344.0255638 | -1.655463429 | 0.308885118 | -5.359479397 | 8.34621E-08 | 2.13999E-05 |
| TMEM30B | 196.7768726 | -1.770955772 | 0.330828766 | -5.353088831 | 8.64654E-08 | 2.18857E-05 |
| CTD-2003C8.2 | 98.3849135 | -2.014030609 | 0.376699271 | -5.34652113 | 8.96608E-08 | 2.24073E-05 |
| C1QTNF9B | 7.932282755 | 2.170444073 | 0.406536655 | 5.338864394 | 9.35306E-08 | 2.30822E-05 |
| ZXDA | 370.378045 | 1.216340458 | 0.228262576 | 5.328689796 | 9.89238E-08 | 2.38177E-05 |
| GDF10 | 107.4545896 | -2.750399017 | 0.520729179 | -5.281822352 | 1.27905E-07 | 3.04245E-05 |
| CPNE5 | 1253.684587 | 1.576359652 | 0.299611147 | 5.261351819 | 1.43E-07 | 3.36101E-05 |
| CRISPLD2 | 2209.349082 | -1.670482539 | 0.318872147 | -5.23872203 | 1.61692E-07 | 3.7195E-05 |
| KAZALD1 | 3306.588003 | 1.341256589 | 0.256045735 | 5.238347714 | 1.62021E-07 | 3.7195E-05 |
| GPC3 | 459.7418474 | -2.141331729 | 0.409856026 | -5.22459496 | 1.74537E-07 | 3.91577E-05 |
| SP6 | 1270.086206 | 2.012796217 | 0.387526132 | 5.19396255 | 2.05865E-07 | 4.56672E-05 |
| DSEL | 1228.084985 | -1.567282948 | 0.302186145 | -5.186481826 | 2.14304E-07 | 4.70111E-05 |
| VGLL3 | 571.0346628 | -2.360464993 | 0.455585273 | -5.181170541 | 2.20498E-07 | 4.78383E-05 |
| MAFF | 690.0915717 | 1.157281371 | 0.224006138 | 5.166293122 | 2.38782E-07 | 5.06912E-05 |
| FRMD1 | 81.84478586 | 2.52006191 | 0.488236352 | 5.1615614 | 2.44899E-07 | 5.08951E-05 |
| MYOD1 | 24.98465719 | -3.27283536 | 0.633956779 | -5.162552827 | 2.43605E-07 | 5.08951E-05 |
| PTPRZ1 | 2920.218331 | 2.495528307 | 0.483801094 | 5.158170035 | 2.49375E-07 | 5.0919E-05 |
| OLFML1 | 681.7988881 | -1.710213712 | 0.332696896 | -5.14045587 | 2.74073E-07 | 5.52145E-05 |
| CAV1 | 3782.187838 | -1.367679808 | 0.267624998 | -5.110433706 | 3.2142E-07 | 6.22137E-05 |
| CST7 | 185.9590854 | -1.950378258 | 0.381644263 | -5.110461363 | 3.21373E-07 | 6.22137E-05 |
| ZFP36 | 3190.80427 | 1.344392987 | 0.262913726 | 5.113437806 | 3.16348E-07 | 6.22137E-05 |
| EYA2 | 794.2361956 | -1.421583677 | 0.278943043 | -5.096322398 | 3.46315E-07 | 6.45028E-05 |
| FAM153A | 9.210768702 | 2.269369623 | 0.445215667 | 5.097236672 | 3.44647E-07 | 6.45028E-05 |
| SPATA6L | 25.47540534 | 1.300154329 | 0.255461771 | 5.089428144 | 3.59145E-07 | 6.57268E-05 |
| COL27A1 | 6263.375586 | -1.466952076 | 0.28930059 | -5.070684705 | 3.96387E-07 | 7.07421E-05 |
| CDH3 | 214.4497095 | -2.626436251 | 0.518764237 | -5.062870689 | 4.1299E-07 | 7.28005E-05 |
| LSAMP | 555.5492513 | -1.995655732 | 0.394394229 | -5.060053073 | 4.1914E-07 | 7.32308E-05 |
| ITPR1 | 1301.711211 | -1.422930478 | 0.281490426 | -5.054987112 | 4.3042E-07 | 7.45419E-05 |
| INPP4B | 451.5101583 | -1.159599448 | 0.229543547 | -5.051762353 | 4.37752E-07 | 7.51525E-05 |
| RP11-455F5.3 | 137.8481579 | 1.535821971 | 0.304353066 | 5.046185306 | 4.50718E-07 | 7.67115E-05 |
| BDNF | 280.5960801 | 1.571033602 | 0.311516244 | 5.043183564 | 4.5785E-07 | 7.70827E-05 |
| PRUNE2 | 1468.947934 | -1.543180499 | 0.306134936 | -5.040850681 | 4.63467E-07 | 7.70827E-05 |
| RASGRP2 | 364.9039973 | 1.545217865 | 0.307142013 | 5.030955715 | 4.88041E-07 | 7.97855E-05 |
| ZDHHC23 | 172.2675149 | 1.403481038 | 0.278989638 | 5.030584821 | 4.88986E-07 | 7.97855E-05 |
| AC007750.5 | 55.34656314 | -1.407818413 | 0.281006923 | -5.009906506 | 5.44565E-07 | 8.74093E-05 |
| COL8A2 | 5412.645317 | -1.992996155 | 0.39831984 | -5.003507116 | 5.62966E-07 | 8.96342E-05 |
| SMOC2 | 1940.343921 | -1.733680685 | 0.348734786 | -4.971344284 | 6.64903E-07 | 0.000103364 |
| EDIL3 | 7106.571352 | -1.171454676 | 0.236797011 | -4.947083889 | 7.53335E-07 | 0.000113535 |
| PDGFRL | 1280.348639 | -1.543439932 | 0.3119241 | -4.948126593 | 7.49312E-07 | 0.000113535 |
| KCNA2 | 40.38965679 | 3.072920862 | 0.621399588 | 4.945160767 | 7.60811E-07 | 0.000113793 |
| ACER2 | 41.43990904 | 1.053564118 | 0.214058094 | 4.921860689 | 8.57252E-07 | 0.000127254 |
| CKMT1B | 28.10729743 | 2.738525685 | 0.55779881 | 4.909522278 | 9.12985E-07 | 0.000133633 |
| RPS6KL1 | 105.037557 | 1.311384877 | 0.267119614 | 4.909354494 | 9.13767E-07 | 0.000133633 |
| EPHB2 | 3552.668734 | -1.252514219 | 0.255904672 | -4.894456245 | 9.85779E-07 | 0.000142327 |
| APCDD1 | 7979.547702 | 1.5421323 | 0.316340385 | 4.874914407 | 1.08856E-06 | 0.000155735 |
| IRX5 | 1186.865017 | 1.17698767 | 0.241944956 | 4.864691917 | 1.14635E-06 | 0.000162823 |
| CASC10 | 394.8209797 | 1.021360833 | 0.210431885 | 4.853641036 | 1.21215E-06 | 0.000169727 |
| CNBD2 | 16.76116614 | 1.462538656 | 0.301322319 | 4.853734902 | 1.21158E-06 | 0.000169727 |
| SSX2IP | 683.6986102 | -1.185778055 | 0.244459387 | -4.850613712 | 1.2308E-06 | 0.000171125 |
| MURC | 153.4712336 | -1.887259496 | 0.389505911 | -4.845265358 | 1.26443E-06 | 0.000173358 |
| AHRR | 325.6462469 | -1.800061147 | 0.37223419 | -4.835829695 | 1.32591E-06 | 0.000176088 |
| PROSER2 | 476.4904348 | 1.195758882 | 0.247101171 | 4.83914696 | 1.30398E-06 | 0.000176088 |
| PTPRN2 | 285.1775067 | 1.640475864 | 0.338993153 | 4.839259579 | 1.30324E-06 | 0.000176088 |
| SOX8 | 287.8543773 | -2.330892396 | 0.482117467 | -4.834698089 | 1.33348E-06 | 0.000176088 |
| UNC13C | 46.66398334 | 2.068674006 | 0.427698518 | 4.836757476 | 1.31974E-06 | 0.000176088 |
| LOXL4 | 1239.574346 | 1.540781218 | 0.318865724 | 4.83206912 | 1.35121E-06 | 0.000176669 |
| RP11-177B4.1 | 16.72319733 | 1.401942783 | 0.290577344 | 4.824680284 | 1.40228E-06 | 0.00018214 |
| PVT1 | 669.5949693 | 1.430191193 | 0.296868104 | 4.81759803 | 1.45297E-06 | 0.000186272 |
| SSC5D | 1068.063199 | -1.469002395 | 0.305638722 | -4.806336002 | 1.53721E-06 | 0.000194546 |
| SMOX | 2933.2974 | 1.215653442 | 0.253407825 | 4.797221394 | 1.60882E-06 | 0.000202311 |
| ARHGAP36 | 15.82515493 | -3.190129034 | 0.665488604 | -4.793664408 | 1.63762E-06 | 0.000203769 |
| SAP25 | 14.52684863 | 1.671870655 | 0.349251437 | 4.787011526 | 1.69283E-06 | 0.000207587 |
| AL022344.7 | 22.66499828 | 1.557535345 | 0.326190717 | 4.774922352 | 1.79777E-06 | 0.000213843 |
| EGFR | 2445.087378 | -1.384848733 | 0.290054066 | -4.77445033 | 1.80199E-06 | 0.000213843 |
| FOXP1 | 2272.339106 | -1.054931059 | 0.221000487 | -4.773433194 | 1.81112E-06 | 0.000213843 |
| TGFBI | 69294.73685 | -1.468530494 | 0.307707708 | -4.772485237 | 1.81966E-06 | 0.000213843 |
| VEPH1 | 172.9627731 | -1.73729617 | 0.363639564 | -4.777522426 | 1.77468E-06 | 0.000213843 |
| IGF2 | 31076.55695 | -1.769332889 | 0.370871126 | -4.770748553 | 1.83543E-06 | 0.000214088 |
| SATB2 | 7743.957085 | 1.199393159 | 0.25145187 | 4.769871701 | 1.84343E-06 | 0.000214088 |
| JMJD1C-AS1 | 110.2333731 | 1.130562154 | 0.237434478 | 4.761575328 | 1.92088E-06 | 0.000221777 |
| ALDH3B2 | 11.63619874 | 1.737259043 | 0.365321685 | 4.755422723 | 1.98032E-06 | 0.000227296 |
| C11orf70 | 120.8330386 | 1.153446344 | 0.242730387 | 4.751965167 | 2.01449E-06 | 0.000228023 |
| TRIM9 | 316.9597559 | -1.84500769 | 0.388316718 | -4.75129605 | 2.02117E-06 | 0.000228023 |
| TRIM17 | 335.6111302 | 1.662871904 | 0.350379734 | 4.74591348 | 2.07567E-06 | 0.000232841 |
| HIST3H2A | 385.949203 | 1.800861677 | 0.380024427 | 4.738805059 | 2.14982E-06 | 0.000238449 |
| MYRIP | 49.83884202 | 1.811715189 | 0.382601585 | 4.735252701 | 2.18782E-06 | 0.000241308 |
| GBP1 | 1949.554012 | -1.3592727 | 0.287342079 | -4.730503463 | 2.23964E-06 | 0.000245651 |
| COL6A1 | 119632.1253 | -1.219558394 | 0.258166719 | -4.723917932 | 2.31344E-06 | 0.000249266 |
| JAG2 | 2563.556819 | 1.08226496 | 0.229144362 | 4.723070433 | 2.3231E-06 | 0.000249266 |
| EFNA5 | 892.2876088 | -1.155842203 | 0.245066001 | -4.7164527 | 2.39992E-06 | 0.000256117 |
| RP4-794H19.1 | 51.2025285 | -1.259671599 | 0.267236406 | -4.713697582 | 2.43262E-06 | 0.00025821 |
| C1orf127 | 50.82457227 | 1.232299198 | 0.261682993 | 4.709129861 | 2.48777E-06 | 0.000261255 |
| FAM221A | 139.9628856 | 1.161895176 | 0.246958552 | 4.704818541 | 2.54092E-06 | 0.000263249 |
| ELN | 3866.410131 | -1.795726417 | 0.382181092 | -4.69862705 | 2.61916E-06 | 0.000265248 |
| GFRA1 | 184.4612517 | 2.50735947 | 0.533321945 | 4.701399392 | 2.58385E-06 | 0.000265248 |
| TENM2 | 1329.132426 | -1.789896724 | 0.380787412 | -4.700514425 | 2.59507E-06 | 0.000265248 |
| TMCC2 | 964.5567317 | 1.263638711 | 0.269031015 | 4.69700012 | 2.6401E-06 | 0.000265936 |
| ARHGAP44 | 937.45908 | 1.323772128 | 0.282056492 | 4.693287225 | 2.6885E-06 | 0.000268076 |
| CCDC13 | 14.7227615 | 1.180431304 | 0.251487144 | 4.693803759 | 2.68171E-06 | 0.000268076 |
| VSNL1 | 262.4092407 | -2.213647148 | 0.471926537 | -4.690660461 | 2.72325E-06 | 0.000270176 |
| UNC5B-AS1 | 48.46645656 | 1.470534617 | 0.313650497 | 4.688449824 | 2.75282E-06 | 0.000271745 |
| AEBP1 | 41815.65 | -1.486331918 | 0.318541997 | -4.66604697 | 3.0705E-06 | 0.000301596 |
| THBS2 | 19268.76198 | -1.499190244 | 0.32157482 | -4.662026224 | 3.13111E-06 | 0.000306028 |
| PSD | 500.1283019 | 1.180480306 | 0.253273216 | 4.660896743 | 3.14835E-06 | 0.000306196 |
| ENTPD3 | 1076.628668 | 1.826639747 | 0.392076839 | 4.658882054 | 3.17931E-06 | 0.000307692 |
| LRIG1 | 989.3991475 | -1.656851773 | 0.355715949 | -4.657794451 | 3.19615E-06 | 0.000307746 |
| ROR1 | 941.4021819 | -1.05804572 | 0.227202672 | -4.656836614 | 3.21105E-06 | 0.000307746 |
| PDLIM3 | 764.5682671 | -2.203327347 | 0.473592605 | -4.652368565 | 3.28144E-06 | 0.000312973 |
| ALDH1B1 | 1625.567604 | -1.066929549 | 0.229769321 | -4.64348132 | 3.42587E-06 | 0.000323622 |
| DUSP27 | 291.8906054 | -2.153406564 | 0.463938933 | -4.641573301 | 3.45766E-06 | 0.000324505 |
| CHRNA1 | 403.6721293 | -2.159070965 | 0.465557986 | -4.637598386 | 3.52481E-06 | 0.000326715 |
| APOD | 308.8965511 | -1.891712263 | 0.408172508 | -4.634590095 | 3.57645E-06 | 0.000329953 |
| MTSS1 | 3511.624795 | 1.231106916 | 0.266544813 | 4.618761478 | 3.86037E-06 | 0.000349612 |
| SIGLEC17P | 35.74762333 | 1.635168477 | 0.353925902 | 4.620087049 | 3.83579E-06 | 0.000349612 |
| TMEM130 | 27.47145829 | -1.949563222 | 0.422050415 | -4.619266212 | 3.851E-06 | 0.000349612 |
| SIX4 | 491.4525874 | -1.182876614 | 0.256780256 | -4.60657152 | 4.09362E-06 | 0.000367365 |
| SYT12 | 1217.254506 | 1.581763924 | 0.343359801 | 4.606724261 | 4.09062E-06 | 0.000367365 |
| CHRM3 | 280.7415214 | 1.444739706 | 0.314571533 | 4.592722344 | 4.37501E-06 | 0.000386104 |
| HMP19 | 8.720095068 | 2.057274597 | 0.447804276 | 4.594137905 | 4.34542E-06 | 0.000386104 |
| SMPD3 | 4816.142188 | 1.772501185 | 0.385959557 | 4.592453159 | 4.38066E-06 | 0.000386104 |
| HEPH | 655.0519488 | -1.604751284 | 0.350387964 | -4.57992697 | 4.65138E-06 | 0.000404057 |
| PTCHD2 | 57.21930483 | -1.955695673 | 0.427076686 | -4.579261148 | 4.66621E-06 | 0.000404057 |
| CH507-513H4.4 | 82.51098811 | -3.251132747 | 0.710729433 | -4.574360647 | 4.77676E-06 | 0.000411824 |
| AP000892.6 | 79.03715269 | -1.049395737 | 0.229482834 | -4.572872477 | 4.81083E-06 | 0.000412957 |
| GALNT8 | 39.59566756 | -1.921038277 | 0.420456499 | -4.568934671 | 4.9021E-06 | 0.00041897 |
| SLC38A4 | 420.7283365 | -2.107240153 | 0.461535157 | -4.565719689 | 4.97784E-06 | 0.00042361 |
| LOXL1 | 5851.232227 | -1.514613152 | 0.332457769 | -4.555806157 | 5.2185E-06 | 0.000442184 |
| HOMER2 | 3555.168205 | 1.281683311 | 0.281488284 | 4.553238567 | 5.28263E-06 | 0.000445705 |
| THBS3 | 2950.163915 | -1.166121107 | 0.256211369 | -4.551402658 | 5.32895E-06 | 0.000447699 |
| C9orf47 | 83.86476304 | 1.124208226 | 0.247090905 | 4.549775832 | 5.37031E-06 | 0.000449263 |
| BMP4 | 2581.008665 | 1.03006925 | 0.226456484 | 4.54864101 | 5.39935E-06 | 0.000449786 |
| CTD-2284J15.1 | 19.75444276 | 1.199334296 | 0.26379057 | 4.546539684 | 5.45351E-06 | 0.000452389 |
| SFRP2 | 1305.87508 | -2.391837067 | 0.526637139 | -4.541717417 | 5.57978E-06 | 0.000459007 |
| NR1D1 | 880.2189635 | -1.125066663 | 0.247929935 | -4.537841157 | 5.6833E-06 | 0.000463659 |
| TNK2-AS1 | 30.72875956 | 1.138563478 | 0.251399099 | 4.528908343 | 5.92892E-06 | 0.000479732 |
| RP11-642P15.1 | 23.1509435 | 1.104189681 | 0.244163346 | 4.522340061 | 6.11597E-06 | 0.000488857 |
| ITIH6 | 44.48564591 | -2.475756377 | 0.54800961 | -4.517724382 | 6.25078E-06 | 0.000493636 |
| BAIAP2L2 | 341.3825799 | 1.463171001 | 0.324039536 | 4.51540889 | 6.31947E-06 | 0.000495609 |
| SLITRK2 | 138.9996091 | 2.90559716 | 0.643763084 | 4.513457249 | 6.37793E-06 | 0.000497706 |
| P4HA3 | 837.705063 | -1.476063316 | 0.327467626 | -4.507509134 | 6.55931E-06 | 0.000507845 |
| AC006277.2 | 21.00882409 | 1.640576693 | 0.36414235 | 4.505316924 | 6.6274E-06 | 0.000511112 |
| PFKFB1 | 26.54055991 | 1.318768999 | 0.292930136 | 4.501991565 | 6.73197E-06 | 0.000513163 |
| PROK2 | 65.15558882 | 2.046734896 | 0.454613069 | 4.502147071 | 6.72704E-06 | 0.000513163 |
| LEFTY1 | 11.5685308 | 1.907842867 | 0.424084251 | 4.498735486 | 6.83588E-06 | 0.000517091 |
| FGFRL1 | 1877.359601 | -1.0924551 | 0.243269784 | -4.49071431 | 7.09847E-06 | 0.000534905 |
| TBXA2R | 840.858055 | 1.096530234 | 0.244309711 | 4.488279358 | 7.18007E-06 | 0.000538997 |
| ABCC11 | 21.29133995 | 1.457385973 | 0.324789701 | 4.487168064 | 7.21761E-06 | 0.000539763 |
| LTBP2 | 2532.750351 | -1.325449533 | 0.295840548 | -4.480283523 | 7.4544E-06 | 0.000550008 |
| TRPV3 | 110.781482 | -1.599245724 | 0.357127115 | -4.478085412 | 7.53155E-06 | 0.000552771 |
| FAP | 2209.382016 | -1.425922059 | 0.318825138 | -4.472426698 | 7.73369E-06 | 0.000565505 |
| SYCE1 | 16.53027797 | 2.762910801 | 0.619111315 | 4.462704422 | 8.09317E-06 | 0.000587439 |
| CHML | 1252.73377 | 1.136428988 | 0.255888934 | 4.441102518 | 8.94991E-06 | 0.000647246 |
| RP11-383H13.1 | 623.95734 | -1.361840373 | 0.306804153 | -4.438793805 | 9.04644E-06 | 0.000651839 |
| ACTA2 | 4425.987895 | -1.339414415 | 0.301825993 | -4.437704003 | 9.09235E-06 | 0.000652765 |
| RP11-71L14.3 | 95.50445978 | 1.889937871 | 0.426132639 | 4.435092965 | 9.20326E-06 | 0.000658333 |
| SMG7-AS1 | 26.07494741 | 1.199557847 | 0.270633063 | 4.432414256 | 9.31838E-06 | 0.000661772 |
| CDH8 | 74.91569823 | 1.921438338 | 0.434003039 | 4.42724628 | 9.54437E-06 | 0.000670749 |
| HGF | 781.3876939 | -1.72314463 | 0.389285667 | -4.426427116 | 9.58067E-06 | 0.000670749 |
| RP11-154H23.3 | 14.91410357 | -1.584878158 | 0.358023007 | -4.426749472 | 9.56637E-06 | 0.000670749 |
| KAL1 | 474.9155995 | -1.698744252 | 0.384517993 | -4.417853736 | 9.96858E-06 | 0.000692992 |
| WDR66 | 203.4846057 | 1.347012506 | 0.305186089 | 4.413741504 | 1.01599E-05 | 0.000703816 |
| PAX1 | 100.8217753 | -2.638551476 | 0.598451977 | -4.408961082 | 1.03868E-05 | 0.00071631 |
| IL16 | 566.9508893 | -1.091229083 | 0.247576095 | -4.407651249 | 1.04498E-05 | 0.000716353 |
| RP11-161M6.2 | 98.8102313 | -1.585735323 | 0.359837152 | -4.406813789 | 1.04902E-05 | 0.000716639 |
| BAMBI | 5576.836157 | 1.35570333 | 0.308137256 | 4.399673535 | 1.08414E-05 | 0.000735538 |
| ISLR2 | 58.92013907 | -1.667553109 | 0.379272778 | -4.396711823 | 1.09903E-05 | 0.000743088 |
| PTGFR | 1690.391875 | -1.59073643 | 0.361895796 | -4.39556482 | 1.10485E-05 | 0.000744473 |
| AC005062.2 | 17.15836037 | 1.44493544 | 0.329252413 | 4.388534093 | 1.14117E-05 | 0.000766332 |
| ITGB6 | 57.88370904 | -2.088200423 | 0.476563554 | -4.381787919 | 1.17709E-05 | 0.00078247 |
| PHLDB2 | 1027.623625 | -1.225629395 | 0.279681619 | -4.382230762 | 1.1747E-05 | 0.00078247 |
| SORCS2 | 476.1734527 | -2.071098326 | 0.473172432 | -4.377047747 | 1.20298E-05 | 0.000796992 |
| AXL | 3315.702379 | -1.037801772 | 0.237253391 | -4.374233669 | 1.2186E-05 | 0.000801959 |
| MAP3K7CL | 368.6421167 | -1.367614731 | 0.312632 | -4.374519336 | 1.217E-05 | 0.000801959 |
| CYP4F3 | 7.772147402 | 2.034119141 | 0.465380351 | 4.370874572 | 1.2375E-05 | 0.000811692 |
| ARSJ | 368.096316 | -1.283497067 | 0.293884464 | -4.367352556 | 1.25762E-05 | 0.000822156 |
| ATP2B2 | 92.10643487 | -1.68546705 | 0.386442334 | -4.36149692 | 1.29176E-05 | 0.000839312 |
| DPY19L2 | 298.1178827 | 1.234905543 | 0.283288891 | 4.359173915 | 1.30554E-05 | 0.000839588 |
| LGI2 | 566.6219998 | -1.211430691 | 0.278033571 | -4.357138193 | 1.31774E-05 | 0.00084468 |
| CD28 | 326.1430777 | 1.287269735 | 0.295712612 | 4.353110703 | 1.34219E-05 | 0.000857183 |
| OXCT2 | 47.92835985 | 1.245637091 | 0.286188765 | 4.352501717 | 1.34593E-05 | 0.000857183 |
| CSRP2 | 3070.154299 | -1.004854606 | 0.231065876 | -4.348779763 | 1.36897E-05 | 0.000869055 |
| AMPH | 643.746077 | -1.518591045 | 0.349778885 | -4.341574372 | 1.41465E-05 | 0.000892317 |
| KIAA0319 | 28.99312362 | 1.605385235 | 0.370257699 | 4.335859158 | 1.45192E-05 | 0.000907128 |
| SLC4A8 | 477.1563582 | 1.210182685 | 0.279508451 | 4.329681908 | 1.49325E-05 | 0.000927082 |
| MYOG | 21.42149073 | -2.786730776 | 0.644215389 | -4.325774923 | 1.51997E-05 | 0.000940711 |
| CACNA1G | 556.9584954 | -1.709769903 | 0.395360851 | -4.32458069 | 1.52822E-05 | 0.000942866 |
| FLJ22447 | 116.6501033 | -1.731907072 | 0.400659194 | -4.322644024 | 1.5417E-05 | 0.000945275 |
| LRRC4B | 88.67243548 | -1.762258644 | 0.407670869 | -4.322748518 | 1.54097E-05 | 0.000945275 |
| CD8A | 185.377734 | -1.798193459 | 0.416357343 | -4.318870527 | 1.5683E-05 | 0.000955645 |
| ZNF521 | 1376.159521 | -1.139499868 | 0.263900626 | -4.31791272 | 1.57512E-05 | 0.000956847 |
| LINC01358 | 25.62085739 | -1.332903465 | 0.308742871 | -4.31719593 | 1.58024E-05 | 0.000957014 |
| COL12A1 | 54125.96332 | -1.153829585 | 0.26755921 | -4.312427093 | 1.61472E-05 | 0.000971934 |
| CTA-228A9.3 | 176.9212081 | 1.572600284 | 0.365126509 | 4.307001119 | 1.65483E-05 | 0.000993048 |
| GFPT2 | 801.5130177 | -1.408979559 | 0.327198855 | -4.306187311 | 1.66092E-05 | 0.000993686 |
| AP000473.5 | 17.41639495 | -1.583223442 | 0.368783177 | -4.293101046 | 1.76195E-05 | 0.001039259 |
| RP11-34A14.3 | 4.65678567 | 2.061992092 | 0.480398418 | 4.292254128 | 1.76868E-05 | 0.001039259 |
| TMEM100 | 203.4990085 | 1.585427103 | 0.369361539 | 4.292344862 | 1.76796E-05 | 0.001039259 |
| ZIC4 | 267.2750482 | -1.505897042 | 0.350705077 | -4.293912862 | 1.75551E-05 | 0.001039259 |
| IFIT2 | 987.0556186 | 1.009052691 | 0.23518243 | 4.290510522 | 1.78263E-05 | 0.001044345 |
| MGP | 6383.614837 | -1.095250995 | 0.255387249 | -4.288589184 | 1.79812E-05 | 0.001047204 |
| RP11-670E13.6 | 34.65131662 | 1.43053473 | 0.333559551 | 4.28869366 | 1.79727E-05 | 0.001047204 |
| CHODL | 33.18570246 | -2.086634672 | 0.48805418 | -4.275416047 | 1.90781E-05 | 0.00110134 |
| FGFR4 | 377.6771764 | -1.993836354 | 0.467275638 | -4.266938379 | 1.98174E-05 | 0.001134072 |
| EFHD1 | 1409.342975 | 1.352893451 | 0.317551008 | 4.26039728 | 2.04064E-05 | 0.001164402 |
| CAV3 | 87.94712919 | -2.334123848 | 0.548466558 | -4.255726832 | 2.08371E-05 | 0.00117986 |
| EPHA4 | 520.6474891 | -1.273193218 | 0.299395954 | -4.252539828 | 2.1136E-05 | 0.001192249 |
| HRH1 | 840.0547613 | -1.039995489 | 0.24515704 | -4.242160412 | 2.21378E-05 | 0.001241668 |
| BOK | 780.0750303 | 1.04639642 | 0.246967122 | 4.236986732 | 2.2654E-05 | 0.001267017 |
| SCG3 | 13.11694175 | 1.703995175 | 0.40247172 | 4.23382586 | 2.29749E-05 | 0.001281337 |
| RP11-108L7.15 | 47.55754118 | 1.001144692 | 0.236721766 | 4.229204214 | 2.34519E-05 | 0.001304258 |
| ENO3 | 370.2460968 | -1.287240642 | 0.304864918 | -4.222331164 | 2.41789E-05 | 0.001329702 |
| KLHL4 | 196.8397488 | -1.721759236 | 0.407732564 | -4.222766068 | 2.41322E-05 | 0.001329702 |
| MOXD1 | 178.4653575 | -2.237666686 | 0.529875985 | -4.223000754 | 2.41071E-05 | 0.001329702 |
| PRSS23 | 5052.3688 | -1.001926534 | 0.238266865 | -4.205060292 | 2.61013E-05 | 0.001404134 |
| SEMA3A | 1809.769273 | -1.085998932 | 0.258243193 | -4.205334202 | 2.60697E-05 | 0.001404134 |
| MYO3A | 261.2991607 | 2.317790397 | 0.551352128 | 4.203829603 | 2.62437E-05 | 0.001407958 |
| ECM2 | 1377.746596 | -1.335434324 | 0.318463148 | -4.193371614 | 2.74839E-05 | 0.001446971 |
| FAM110D | 216.1429007 | 1.109437292 | 0.264527896 | 4.194027584 | 2.74045E-05 | 0.001446971 |
| LAYN | 771.8291531 | -1.043105078 | 0.248702515 | -4.194187893 | 2.73851E-05 | 0.001446971 |
| RTN1 | 387.767161 | 1.209290979 | 0.288622177 | 4.189875469 | 2.79108E-05 | 0.001465538 |
| CH507-513H4.3 | 79.32017893 | -2.461872444 | 0.58801779 | -4.186731227 | 2.83001E-05 | 0.001478117 |
| ERBB4 | 250.3525687 | 1.68764744 | 0.405354359 | 4.163387912 | 3.1356E-05 | 0.001607952 |
| TEX29 | 26.80376825 | -1.768957488 | 0.425386483 | -4.158471314 | 3.20384E-05 | 0.001630245 |
| CDK15 | 82.73072654 | -1.378904226 | 0.33185223 | -4.155175405 | 3.25038E-05 | 0.001641235 |
| ANGPT1 | 2908.552469 | 1.291955666 | 0.311869322 | 4.142618642 | 3.43363E-05 | 0.001724939 |
| ITGA11 | 6917.654237 | -1.221958407 | 0.295100805 | -4.140816928 | 3.46071E-05 | 0.001729742 |
| DNAH2 | 150.6168183 | 1.791741034 | 0.433611979 | 4.132129925 | 3.59417E-05 | 0.001787399 |
| PTHLH | 38.50061148 | -1.692457078 | 0.410088158 | -4.127056698 | 3.67436E-05 | 0.001818117 |
| ACTA2-AS1 | 42.52667825 | -1.192399262 | 0.289429742 | -4.119822842 | 3.79164E-05 | 0.001871458 |
| MYEF2 | 475.2844373 | 1.476538155 | 0.358888918 | 4.114192664 | 3.88537E-05 | 0.001898733 |
| RP11-333I13.1 | 16.5102381 | -1.270684459 | 0.308840367 | -4.114372972 | 3.88233E-05 | 0.001898733 |
| ELANE | 15.67461225 | -1.927869135 | 0.468670578 | -4.11348445 | 3.89731E-05 | 0.001899526 |
| FOS | 5782.279739 | 1.431580754 | 0.348066065 | 4.112956988 | 3.90623E-05 | 0.001899526 |
| RP11-150O12.6 | 29.544552 | -1.806105328 | 0.441976983 | -4.086423949 | 4.38073E-05 | 0.002079057 |
| SERTAD4 | 407.7884016 | -1.42084899 | 0.348007417 | -4.082812375 | 4.44939E-05 | 0.002101541 |
| CLEC2L | 53.64472613 | 2.616174111 | 0.641294464 | 4.07952081 | 4.51286E-05 | 0.002126387 |
| PXDNL | 118.6585991 | 1.33313669 | 0.326831583 | 4.078971436 | 4.52354E-05 | 0.002126387 |
| VPS9D1-AS1 | 308.395257 | 1.365179802 | 0.335309954 | 4.071396586 | 4.67321E-05 | 0.002170899 |
| CHST8 | 44.67253382 | 1.889789304 | 0.464995596 | 4.064101509 | 4.82178E-05 | 0.002229426 |
| TRIM50 | 11.9900949 | 1.539723918 | 0.379335929 | 4.05899837 | 4.92837E-05 | 0.002252332 |
| RP11-280O1.2 | 15.93596362 | 1.818448175 | 0.449371284 | 4.046649709 | 5.19559E-05 | 0.002358081 |
| SPON2 | 2508.598122 | -1.495128062 | 0.370316513 | -4.037432866 | 5.40393E-05 | 0.002424768 |
| RPH3A | 12.05845548 | 1.752563027 | 0.434403772 | 4.034410239 | 5.47396E-05 | 0.00244032 |
| TOX | 419.3672749 | -1.462574783 | 0.362728449 | -4.03214798 | 5.52694E-05 | 0.002446599 |
| C1R | 5314.002118 | -1.117973164 | 0.277743143 | -4.025205274 | 5.69256E-05 | 0.002499908 |
| KCNJ3 | 221.4223305 | 2.490449952 | 0.61866746 | 4.025506617 | 5.68528E-05 | 0.002499908 |
| RIMKLA | 66.07878077 | 1.373689936 | 0.341423766 | 4.023416273 | 5.736E-05 | 0.002499908 |
| CTNND2 | 234.4811835 | -1.57416685 | 0.391685087 | -4.018960392 | 5.84555E-05 | 0.002536455 |
| KLHL41 | 105.0962663 | -1.898209026 | 0.472276017 | -4.019278888 | 5.83765E-05 | 0.002536455 |
| APBA1 | 266.4760512 | -1.001163652 | 0.249406054 | -4.014191455 | 5.96499E-05 | 0.002576955 |
| RP11-517I3.2 | 94.07237264 | -1.081435599 | 0.270169266 | -4.002807635 | 6.25952E-05 | 0.002686559 |
| CITED4 | 220.8114259 | 1.059233172 | 0.265014176 | 3.996892492 | 6.41794E-05 | 0.002742629 |
| GAS1 | 2906.722052 | -1.239076455 | 0.310110468 | -3.995596998 | 6.45314E-05 | 0.002751715 |
| TNNT3 | 753.029885 | -2.003596441 | 0.502105344 | -3.990390589 | 6.59646E-05 | 0.002787026 |
| CTD-3222D19.9 | 9.598882291 | -1.042179458 | 0.261470803 | -3.985834929 | 6.72433E-05 | 0.002819703 |
| ETF1P2 | 18.0519 | 1.089629973 | 0.273435569 | 3.984960618 | 6.74913E-05 | 0.002819703 |
| KIAA1549L | 593.1197436 | -1.311083509 | 0.329148948 | -3.983252928 | 6.79783E-05 | 0.002827871 |
| TKTL1 | 42.38261731 | 1.480950849 | 0.371813198 | 3.983050783 | 6.80362E-05 | 0.002827871 |
| GALNT6 | 729.3671954 | 1.009751461 | 0.253689264 | 3.980268796 | 6.88374E-05 | 0.002843215 |
| MRVI1 | 212.5767553 | -1.140344085 | 0.286499128 | -3.980270707 | 6.88368E-05 | 0.002843215 |
| FGFBP2 | 1033.257649 | -2.205761923 | 0.555662258 | -3.969609038 | 7.19907E-05 | 0.002948779 |
| LOXL1-AS1 | 229.9018168 | -1.110580109 | 0.279815697 | -3.968970005 | 7.2184E-05 | 0.002950576 |
| CTD-2334D19.1 | 11.00085027 | -1.766878952 | 0.445623755 | -3.964956832 | 7.34092E-05 | 0.00299446 |
| HYDIN | 48.04997453 | 1.373579871 | 0.347133364 | 3.956922656 | 7.59215E-05 | 0.003084194 |
| KIF26B | 525.6151913 | -1.360015916 | 0.343795775 | -3.955883158 | 7.62524E-05 | 0.003091276 |
| CCDC144NL-AS1 | 263.937797 | 1.751905576 | 0.443588667 | 3.949392093 | 7.83499E-05 | 0.003163318 |
| ARHGAP28 | 1559.531662 | -1.011877727 | 0.256457123 | -3.945601953 | 7.95998E-05 | 0.003200688 |
| KLHL33 | 19.70678026 | -2.013200638 | 0.510201966 | -3.945889609 | 7.95042E-05 | 0.003200688 |
| MYO16 | 162.763458 | 2.091472247 | 0.530157016 | 3.945005317 | 7.97982E-05 | 0.003202146 |
| FAM153B | 10.8674534 | 1.898263769 | 0.481273832 | 3.94424887 | 8.00505E-05 | 0.003205754 |
| FAIM2 | 63.95770079 | -1.505170062 | 0.382020003 | -3.940029451 | 8.14716E-05 | 0.003236406 |
| BAIAP2L1 | 426.2276076 | -1.228033339 | 0.311888943 | -3.937405817 | 8.23672E-05 | 0.003252353 |
| FRMD3 | 157.1397281 | 1.194791936 | 0.30342031 | 3.937745418 | 8.22508E-05 | 0.003252353 |
| RP1-140K8.5 | 21.21729842 | -2.038440022 | 0.518678136 | -3.930067379 | 8.49221E-05 | 0.003320032 |
| NTN4 | 539.2784513 | -1.195233361 | 0.304225632 | -3.928772716 | 8.53805E-05 | 0.003331358 |
| RP11-108M9.3 | 38.05125444 | 1.720722422 | 0.438232337 | 3.926507187 | 8.61883E-05 | 0.003349638 |
| PRSS12 | 161.0453514 | -1.868897248 | 0.476297569 | -3.923801774 | 8.71625E-05 | 0.003374213 |
| DES | 1936.14488 | -2.401181258 | 0.612065089 | -3.923081552 | 8.74235E-05 | 0.003377697 |
| CXCL2 | 45.9724763 | 1.597137834 | 0.407175257 | 3.922482533 | 8.76412E-05 | 0.003379494 |
| PCDH10 | 269.6505611 | -1.941556616 | 0.495044312 | -3.921985509 | 8.78223E-05 | 0.003379874 |
| C1S | 4358.391251 | -1.342112707 | 0.343554329 | -3.906551578 | 9.36227E-05 | 0.003554601 |
| PGBD5 | 445.7281862 | 1.506647806 | 0.385800543 | 3.905250615 | 9.41278E-05 | 0.003566919 |
| COL3A1 | 307425.9106 | -1.304246613 | 0.33470056 | -3.896756594 | 9.74895E-05 | 0.003659193 |
| LRRC10B | 70.38955274 | 1.173923029 | 0.301245757 | 3.896894813 | 9.74339E-05 | 0.003659193 |
| PKNOX2 | 152.0034562 | -1.398617309 | 0.359289969 | -3.892725731 | 9.91242E-05 | 0.00371349 |
| MAOB | 259.0134349 | 1.373323213 | 0.353224211 | 3.887964554 | 0.000101088 | 0.003776839 |
| SYNC | 170.0213321 | -1.126343676 | 0.289719655 | -3.887701978 | 0.000101198 | 0.003776839 |
| PARD6B | 112.3777245 | 1.181341696 | 0.30396425 | 3.886449461 | 0.000101721 | 0.003782071 |
| RP11-356J5.12 | 435.1458399 | 1.027381389 | 0.26457042 | 3.883205797 | 0.000103088 | 0.00381852 |
| PDE3A | 545.2123435 | -1.155957054 | 0.297766693 | -3.882089845 | 0.000103563 | 0.003828907 |
| ABCA4 | 303.8831342 | 1.417858144 | 0.36553519 | 3.878855398 | 0.000104949 | 0.003872917 |
| AC073850.6 | 6.173600107 | 1.701347233 | 0.438937955 | 3.876054038 | 0.000106164 | 0.00389911 |
| PLEKHB1 | 82.24526392 | 1.08574466 | 0.280130434 | 3.875853987 | 0.000106251 | 0.00389911 |
| RBM20 | 262.0037404 | 1.306445989 | 0.337065633 | 3.875939469 | 0.000106214 | 0.00389911 |
| UPB1 | 15.75066466 | 1.26488194 | 0.326537071 | 3.873624324 | 0.000107229 | 0.003927673 |
| RP11-572M11.3 | 9.579555908 | 1.536301047 | 0.396959633 | 3.870169454 | 0.00010876 | 0.003969027 |
| RP11-45A17.4 | 12.32114464 | 1.322269374 | 0.341717681 | 3.869478956 | 0.000109068 | 0.00397294 |
| PRR33 | 244.192827 | -1.022857069 | 0.264702701 | -3.864173146 | 0.000111466 | 0.004033615 |
| DNM1P47 | 21.62337864 | -1.379458308 | 0.357454317 | -3.859117769 | 0.000113797 | 0.00409981 |
| RP11-251M1.1 | 7.76409874 | 1.192558656 | 0.309689457 | 3.850820977 | 0.000117723 | 0.004208795 |
| HMCN1 | 3753.675024 | -1.067001712 | 0.277518613 | -3.844793325 | 0.000120654 | 0.004279346 |
| AP000473.8 | 13.04114188 | -1.706242321 | 0.444239331 | -3.840817783 | 0.000122625 | 0.004316404 |
| MYLK4 | 834.5267562 | 1.428597413 | 0.371956421 | 3.840765567 | 0.000122651 | 0.004316404 |
| KCND2 | 33.06870819 | 2.053885556 | 0.534820582 | 3.840326316 | 0.000122871 | 0.004316441 |
| IDI2-AS1 | 9.427461562 | 1.160070411 | 0.302259801 | 3.837991048 | 0.000124045 | 0.004349948 |
| DNAH6 | 46.31124291 | 1.195676116 | 0.31180756 | 3.834660443 | 0.000125738 | 0.004393704 |
| LINC00540 | 13.01063115 | 1.643478584 | 0.429296517 | 3.828306355 | 0.000129028 | 0.00450071 |
| CTB-131B5.2 | 7.161744495 | 1.576978682 | 0.412102085 | 3.826669992 | 0.000129888 | 0.004506833 |
| MEIS3P2 | 76.26809253 | 1.305303188 | 0.341093563 | 3.826818593 | 0.00012981 | 0.004506833 |
| SCG2 | 67.33609296 | -1.74349134 | 0.456169075 | -3.822028791 | 0.000132358 | 0.004560469 |
| SMOC1 | 764.3369078 | -1.757272429 | 0.459954149 | -3.820538269 | 0.000133161 | 0.004580126 |
| MIR99AHG | 159.7818856 | -1.28545628 | 0.336592349 | -3.819030006 | 0.000133977 | 0.004600204 |
| MEGF6 | 639.9256791 | -1.262838801 | 0.330803526 | -3.817488943 | 0.000134817 | 0.004620987 |
| CAP2 | 349.312263 | -1.752098718 | 0.459143045 | -3.81601929 | 0.000135622 | 0.004632497 |
| RP11-893F2.14 | 9.170203397 | -1.738881419 | 0.456099931 | -3.812500946 | 0.000137568 | 0.004658661 |
| LINC00598 | 13.59313697 | 1.242449425 | 0.326096758 | 3.810063714 | 0.000138931 | 0.004688743 |
| LRRN3 | 1356.549035 | -1.074515962 | 0.282507473 | -3.803495717 | 0.000142668 | 0.004790312 |
| PHYHIP | 35.98577204 | 1.154303356 | 0.303434356 | 3.804128743 | 0.000142304 | 0.004790312 |
| PLXDC1 | 1927.98448 | -1.002322229 | 0.2636717 | -3.801402388 | 0.000143879 | 0.004814596 |
| DLK1 | 27.12020834 | -2.606841354 | 0.686733091 | -3.7960037 | 0.000147047 | 0.004879252 |
| EGR1 | 6210.11815 | 1.003207906 | 0.264731286 | 3.789532853 | 0.000150931 | 0.004991336 |
| CTD-2015G9.2 | 14.79787498 | 1.281467153 | 0.338374298 | 3.787129107 | 0.000152398 | 0.005031425 |
| COL6A3 | 87067.97516 | -1.03351489 | 0.272945324 | -3.786527184 | 0.000152767 | 0.005033843 |
| FAM153C | 5.272558613 | 1.955717842 | 0.516597709 | 3.785765611 | 0.000153236 | 0.005033843 |
| RET | 125.0332851 | 1.08877753 | 0.287595333 | 3.785796932 | 0.000153217 | 0.005033843 |
| PDCD6IPP2 | 52.2713042 | 1.087279866 | 0.288573675 | 3.767772187 | 0.000164711 | 0.005322238 |
| IMPG1 | 14.50481377 | 1.202893976 | 0.320023544 | 3.758767127 | 0.000170753 | 0.005463809 |
| NR2F2-AS1 | 46.90144357 | 1.314783971 | 0.350452469 | 3.751675586 | 0.000175657 | 0.005593532 |
| RBM24 | 96.39231922 | -1.895300179 | 0.506035922 | -3.745386632 | 0.000180116 | 0.005707919 |
| RP11-243M5.2 | 33.50396967 | 1.626717408 | 0.434611071 | 3.74292676 | 0.000181889 | 0.005745663 |
| CLDN18 | 33.31057173 | -1.081850144 | 0.289166717 | -3.741267856 | 0.000183094 | 0.005774489 |
| PRDM16 | 142.8138711 | -1.702431637 | 0.455485827 | -3.737617146 | 0.000185773 | 0.005825246 |
| RSPH10B2 | 7.057248444 | 1.461865634 | 0.391138125 | 3.737466488 | 0.000185884 | 0.005825246 |
| RP1L1 | 13.99630195 | 1.21943669 | 0.32655737 | 3.734218857 | 0.000188299 | 0.005891571 |
| LINC00924 | 29.55581421 | 1.523424239 | 0.408072504 | 3.733219521 | 0.000189048 | 0.005896317 |
| RP4-740C4.5 | 39.0582607 | -1.01608824 | 0.2730748 | -3.720915442 | 0.000198502 | 0.006161986 |
| LTK | 454.0807021 | 1.259985311 | 0.338671578 | 3.720375119 | 0.000198927 | 0.006165491 |
| PAWR | 459.2321018 | -1.297718285 | 0.349517938 | -3.712880353 | 0.000204914 | 0.006252879 |
| GRAMD1B | 1927.524035 | 1.029115858 | 0.277829439 | 3.704128193 | 0.000212119 | 0.00640511 |
| KCNQ4 | 42.32226717 | 1.342298595 | 0.362579114 | 3.702084714 | 0.000213835 | 0.006427673 |
| CELSR3 | 213.856773 | -1.132659998 | 0.30616282 | -3.699534766 | 0.000215995 | 0.006480836 |
| NECAB2 | 44.9018695 | 1.566569611 | 0.42351776 | 3.698946681 | 0.000216496 | 0.006486012 |
| CPE | 42228.61264 | 1.03687758 | 0.280415363 | 3.697648971 | 0.000217606 | 0.006509372 |
| GNGT2 | 134.9130822 | 1.16277335 | 0.314693977 | 3.694933603 | 0.000219944 | 0.006559455 |
| MXRA5 | 20967.97187 | -1.025754104 | 0.277837462 | -3.691921523 | 0.000222566 | 0.006581197 |
| PARD6G-AS1 | 143.7534676 | 1.039680355 | 0.28171883 | 3.690489389 | 0.000223823 | 0.006581197 |
| RPGRIP1 | 41.24858716 | 1.012522956 | 0.274303929 | 3.691244813 | 0.000223159 | 0.006581197 |
| ADAP1 | 203.6320442 | 1.097990025 | 0.298089891 | 3.683419187 | 0.000230126 | 0.006671634 |
| SEMA3C | 933.227352 | -1.42643898 | 0.387169929 | -3.684271097 | 0.000229358 | 0.006671634 |
| SIM2 | 377.4874847 | -1.290982667 | 0.350368661 | -3.68464081 | 0.000229025 | 0.006671634 |
| ABCA3 | 497.4814911 | 1.108197185 | 0.301805991 | 3.671885968 | 0.000240767 | 0.006929249 |
| IGHA1 | 985.8895249 | -2.211399858 | 0.602524786 | -3.670222221 | 0.00024234 | 0.006964356 |
| RIMS1 | 105.6936725 | -2.133678724 | 0.582465318 | -3.663185875 | 0.000249098 | 0.007089039 |
| RP11-563J2.2 | 11.83407904 | 1.157830777 | 0.316034258 | 3.663624265 | 0.000248671 | 0.007089039 |
| KCNB1 | 392.0003225 | 1.441668368 | 0.393677584 | 3.662053484 | 0.000250202 | 0.007107526 |
| CACNA2D1 | 1009.310157 | -1.013838915 | 0.276932607 | -3.660958988 | 0.000251273 | 0.007127705 |
| NTNG2 | 100.6594477 | -1.38848165 | 0.379424398 | -3.659442189 | 0.000252765 | 0.007148376 |
| PRR16 | 207.5193384 | -1.174941829 | 0.321116866 | -3.658922816 | 0.000253278 | 0.007148376 |
| MYO1A | 7.968899031 | 1.338170625 | 0.366197883 | 3.654228182 | 0.000257957 | 0.007234149 |
| DNAJC9-AS1 | 10.26922351 | 1.263968218 | 0.346209485 | 3.650876925 | 0.000261346 | 0.007308446 |
| NR2E3 | 12.61988283 | 1.44393601 | 0.395933152 | 3.646918689 | 0.000265404 | 0.007380096 |
| ACTC1 | 496.3166562 | -2.17984448 | 0.597948833 | -3.645536808 | 0.000266834 | 0.007409435 |
| PLS1 | 245.6456041 | 1.188346508 | 0.326048675 | 3.644690496 | 0.000267714 | 0.007423417 |
| S100A2 | 273.2642101 | -1.227436102 | 0.336999545 | -3.642248546 | 0.000270267 | 0.007462769 |
| LMOD3 | 253.1326352 | -1.465453478 | 0.402551433 | -3.640413023 | 0.000272201 | 0.007496155 |
| ANO5 | 5672.023813 | 1.390272221 | 0.382376432 | 3.635873195 | 0.000277041 | 0.007586148 |
| FMN2 | 24.48382058 | 1.416927435 | 0.389692116 | 3.636017707 | 0.000276885 | 0.007586148 |
| RP11-532M24.1 | 20.6201747 | 1.090432808 | 0.300295602 | 3.63119806 | 0.000282109 | 0.007702195 |
| RP11-181C3.1 | 13.59860655 | 1.381451768 | 0.380599498 | 3.62967312 | 0.00028378 | 0.007717184 |
| PPP1R1C | 27.18656202 | -1.399627512 | 0.386006152 | -3.625920222 | 0.000287934 | 0.007787241 |
| HPSE2 | 6.63083885 | 1.656469866 | 0.456894558 | 3.625497039 | 0.000288406 | 0.007789335 |
| RP11-437B10.1 | 35.67560798 | 1.041894174 | 0.287922601 | 3.618660605 | 0.000296132 | 0.007932874 |
| KCNC1 | 33.73318258 | -1.806315799 | 0.499608567 | -3.615462016 | 0.000299812 | 0.008020592 |
| PCDHGB7 | 274.4804324 | -1.31804963 | 0.364645622 | -3.614604293 | 0.000300807 | 0.008036302 |
| MYH11 | 446.5212232 | -1.102320786 | 0.30510488 | -3.612924142 | 0.000302763 | 0.008077643 |
| FAM180A | 495.9492822 | -1.667498453 | 0.461682484 | -3.61178626 | 0.000304095 | 0.008097979 |
| GYS2 | 7.464547044 | 1.590273943 | 0.440327281 | 3.611572598 | 0.000304346 | 0.008097979 |
| AOC4P | 19.76623403 | 1.176546711 | 0.325930709 | 3.609806257 | 0.000306426 | 0.008142348 |
| LINC00694 | 1.606136192 | 2.121503643 | 0.588590151 | 3.604381824 | 0.000312897 | 0.008280859 |
| BHLHE22 | 67.46880916 | -2.266966672 | 0.629923167 | -3.598798693 | 0.000319691 | 0.008415533 |
| CHMP4C | 587.0517222 | 1.616670519 | 0.449460163 | 3.596916153 | 0.000322012 | 0.008465363 |
| PKHD1 | 24.54431315 | 1.684810344 | 0.468758688 | 3.59419545 | 0.000325396 | 0.008513662 |
| ZNF385D | 154.2465994 | -1.545658346 | 0.43009183 | -3.593786808 | 0.000325907 | 0.008513662 |
| GBP5 | 422.665936 | -1.346843434 | 0.374868306 | -3.592844242 | 0.000327088 | 0.008530648 |
| ASPN | 3770.697481 | -1.733227318 | 0.482488934 | -3.592263361 | 0.000327818 | 0.008538412 |
| RPL3L | 19.36923857 | 1.028414025 | 0.286358738 | 3.591348507 | 0.000328971 | 0.008557157 |
| ABTB2 | 299.0481869 | -1.003582643 | 0.279894641 | -3.585572918 | 0.000336339 | 0.008725809 |
| EPCAM | 397.2972417 | 1.469384915 | 0.40977036 | 3.58587409 | 0.000335951 | 0.008725809 |
| C1orf64 | 4.22134161 | 2.061432544 | 0.57500436 | 3.585072894 | 0.000336984 | 0.008728774 |
| RNF150 | 989.8480401 | -1.153685216 | 0.321933147 | -3.583617367 | 0.000338868 | 0.008746127 |
| SYT7 | 219.3600757 | -1.390042046 | 0.388275312 | -3.580042313 | 0.000343539 | 0.008819873 |
| HFE2 | 25.816887 | -1.921576171 | 0.536876889 | -3.579174688 | 0.000344681 | 0.008837711 |
| APCDD1L | 765.9908378 | -1.782643341 | 0.498933404 | -3.572908381 | 0.000353038 | 0.008987197 |
| CAPN6 | 133.8761464 | -1.856960399 | 0.519881882 | -3.571889045 | 0.000354416 | 0.009000763 |
| RARRES1 | 451.4461266 | 1.260863998 | 0.353600061 | 3.56579123 | 0.00036276 | 0.009123531 |
| SYT13 | 151.6630665 | 1.894124152 | 0.531265822 | 3.565303982 | 0.000363435 | 0.009128869 |
| GRM4 | 62.27856738 | 1.419744678 | 0.398318793 | 3.564342693 | 0.000364769 | 0.009150746 |
| AC144831.1 | 234.4233646 | -1.182361232 | 0.332647624 | -3.554395543 | 0.000378849 | 0.009384712 |
| PIANP | 86.20215488 | -1.323366748 | 0.37248409 | -3.552814157 | 0.000381134 | 0.009429475 |
| GREB1L | 68.23422409 | 1.424473067 | 0.401297678 | 3.549666853 | 0.000385719 | 0.009519061 |
| SIM1 | 65.3066839 | -1.711521943 | 0.482839266 | -3.544703305 | 0.000393055 | 0.009627903 |
| GRB7 | 25.99447496 | 1.39219369 | 0.393017023 | 3.542324146 | 0.000396618 | 0.009703128 |
| PGPEP1L | 5.055526877 | 1.64042467 | 0.463382852 | 3.540106552 | 0.000399966 | 0.00976084 |
| SHBG | 19.52920987 | 1.034380047 | 0.292727234 | 3.533596894 | 0.000409946 | 0.009955178 |
| DNAH10 | 488.3661922 | 1.086451011 | 0.307597581 | 3.532053174 | 0.000412346 | 0.009988903 |
| LRRC17 | 2418.775506 | -1.008308524 | 0.286058758 | -3.524830109 | 0.000423754 | 0.01021831 |
| PCDHB6 | 135.8198876 | -1.253021439 | 0.355492547 | -3.524747426 | 0.000423887 | 0.01021831 |
| GABRP | 11.21993719 | 1.152616678 | 0.327307572 | 3.521509361 | 0.000429098 | 0.010281156 |
| DOCK3 | 188.6091508 | 1.304558244 | 0.371058506 | 3.515775065 | 0.000438472 | 0.010442411 |
| EGR2 | 201.3322505 | 1.000843781 | 0.285204156 | 3.509218783 | 0.000449425 | 0.010639087 |
| SOX6 | 975.8962546 | -1.019761246 | 0.290591288 | -3.509262973 | 0.00044935 | 0.010639087 |
| IGSF10 | 1169.873153 | -1.249585476 | 0.356298687 | -3.507129054 | 0.000452969 | 0.010684558 |
| KHDRBS3 | 180.8086775 | -1.180387387 | 0.336750924 | -3.505223897 | 0.000456223 | 0.010730002 |
| RLTPR | 85.5585663 | 1.062890076 | 0.303492226 | 3.502198683 | 0.000461435 | 0.010832484 |
| CABP4 | 264.7416162 | 1.033413524 | 0.295289174 | 3.499666141 | 0.000465841 | 0.010909968 |
| RP11-338I21.1 | 14.9378622 | 1.131760241 | 0.323695579 | 3.496372256 | 0.00047163 | 0.01098042 |
| ANKRD29 | 58.9625665 | -1.123136874 | 0.321326716 | -3.495311213 | 0.000473509 | 0.01099056 |
| COL14A1 | 6620.479153 | -1.62042923 | 0.463535961 | -3.495800467 | 0.000472642 | 0.01099056 |
| RP11-1299A16.3 | 14.59310313 | -1.252813968 | 0.358440108 | -3.495183549 | 0.000473736 | 0.01099056 |
| RP11-98D18.9 | 10.59344059 | 1.086100606 | 0.310811849 | 3.49439897 | 0.00047513 | 0.011009976 |
| BNC1 | 41.06333831 | -1.741360589 | 0.499198092 | -3.488315793 | 0.000486074 | 0.011130793 |
| FBLN1 | 9093.734873 | -1.111431064 | 0.318630761 | -3.488147417 | 0.00048638 | 0.011130793 |
| PAQR9 | 36.98238356 | 1.852454788 | 0.531085136 | 3.488056174 | 0.000486546 | 0.011130793 |
| PDE6C | 13.25000091 | 1.16385383 | 0.333954317 | 3.485068981 | 0.00049201 | 0.01121681 |
| RP4-614O4.12 | 22.85650305 | 1.115763596 | 0.320717487 | 3.478960894 | 0.000503362 | 0.011402129 |
| EPHB6 | 211.5992542 | -1.152930169 | 0.331530406 | -3.477600084 | 0.000505924 | 0.011441535 |
| DSC3 | 46.23013223 | -2.084852943 | 0.599837588 | -3.475695729 | 0.00050953 | 0.011496801 |
| RP11-309L24.6 | 8.47839407 | -1.709042086 | 0.491712414 | -3.475694406 | 0.000509533 | 0.011496801 |
| RP11-370I10.12 | 12.81810634 | 1.304725429 | 0.3755425 | 3.474241743 | 0.000512299 | 0.011532867 |
| RPS6KA6 | 654.3596129 | 1.257651066 | 0.362142002 | 3.472811935 | 0.000515036 | 0.011568098 |
| ALOX15B | 17.1198239 | 1.213443137 | 0.349475277 | 3.472185926 | 0.000516239 | 0.011581932 |
| SLC8A3 | 2721.000945 | 1.397513582 | 0.402541621 | 3.471724432 | 0.000517127 | 0.01158869 |
| ADD3-AS1 | 20.81717062 | 1.152862146 | 0.332515074 | 3.467097395 | 0.000526111 | 0.011769531 |
| IGSF11 | 219.2554519 | 1.395716148 | 0.402647388 | 3.466348446 | 0.000527579 | 0.011770121 |
| MT1A | 75.13474691 | 1.484397077 | 0.428756981 | 3.462094243 | 0.000535989 | 0.011876587 |
| CALN1 | 25.28021013 | 1.754580738 | 0.506970389 | 3.46091365 | 0.000538345 | 0.01191542 |
| CLIP1-AS1 | 9.439464786 | 1.250149986 | 0.361299241 | 3.460151158 | 0.000539872 | 0.011929613 |
| ADCY10 | 30.23920081 | 1.16746118 | 0.337763574 | 3.456444887 | 0.000547351 | 0.012060662 |
| SLC26A9 | 16.62852885 | 1.275891507 | 0.36926204 | 3.455246871 | 0.000549789 | 0.012100878 |
| PKHD1L1 | 20.44664307 | 1.606487925 | 0.465256984 | 3.452904479 | 0.000554585 | 0.012125585 |
| SUSD4 | 271.3610531 | 1.150989536 | 0.333251741 | 3.453814026 | 0.000552718 | 0.012125585 |
| CXCL3 | 34.99669694 | 1.221166277 | 0.353820666 | 3.451370691 | 0.000557747 | 0.012180971 |
| CD52 | 195.3686678 | -1.190577545 | 0.345250361 | -3.448446924 | 0.00056382 | 0.012245879 |
| CAMSAP3 | 23.2530729 | 1.477288143 | 0.429904806 | 3.436314556 | 0.000589686 | 0.012627081 |
| NME9 | 17.98614578 | 1.017488396 | 0.296146676 | 3.435758285 | 0.000590898 | 0.012639326 |
| HS6ST2 | 264.8086942 | -1.445058572 | 0.420766805 | -3.434345474 | 0.000593987 | 0.012691643 |
| BMP8B | 3703.42234 | 1.115595756 | 0.325482143 | 3.427517544 | 0.000609127 | 0.012857294 |
| KLB | 24.12610016 | 1.05798526 | 0.308657048 | 3.427704855 | 0.000608707 | 0.012857294 |
| VWA3A | 13.06864411 | 1.328300184 | 0.387780767 | 3.425389546 | 0.000613919 | 0.012918998 |
| NPC1L1 | 11.8113038 | 1.135898284 | 0.331755539 | 3.423901485 | 0.00061729 | 0.012965059 |
| GEM | 710.7948725 | -1.138113557 | 0.333049343 | -3.417252069 | 0.000632567 | 0.013187715 |
| TSHZ2 | 201.5186119 | -1.199375975 | 0.351203781 | -3.415042886 | 0.00063772 | 0.013239222 |
| UPK3BL | 31.84697783 | -1.078299167 | 0.315736657 | -3.415185227 | 0.000637387 | 0.013239222 |
| ROBO2 | 2750.863443 | 1.117831073 | 0.327686761 | 3.411279321 | 0.000646588 | 0.013339175 |
| SVOP | 7.576169298 | 1.361831121 | 0.399389222 | 3.409784359 | 0.000650143 | 0.013370589 |
| A2ML1 | 16.48046579 | 1.163554905 | 0.341479349 | 3.407394648 | 0.000655862 | 0.013460174 |
| RP11-88E10.4 | 60.62849498 | 1.110039724 | 0.32636562 | 3.401215252 | 0.00067087 | 0.013696983 |
| CPA4 | 49.26548004 | -1.520808942 | 0.447513008 | -3.398356955 | 0.000677919 | 0.013769707 |
| ABCC8 | 22.41665786 | 1.705690553 | 0.50217377 | 3.396614188 | 0.000682251 | 0.013829239 |
| NEGR1 | 464.0048408 | -1.37978192 | 0.406631793 | -3.393197343 | 0.000690819 | 0.013931389 |
| CXADR | 790.6485772 | 1.068853799 | 0.31513444 | 3.391739087 | 0.000694505 | 0.013977187 |
| MYBPH | 22.80678196 | -2.428242175 | 0.716547897 | -3.388806505 | 0.000701975 | 0.014098777 |
| COL4A4 | 118.3325825 | 1.30195284 | 0.38440297 | 3.386947922 | 0.000706748 | 0.0141228 |
| ZIC1 | 1049.373726 | -1.21327343 | 0.358177413 | -3.387353268 | 0.000705705 | 0.0141228 |
| RP13-608F4.5 | 7.563019687 | 1.438817137 | 0.425913346 | 3.378192187 | 0.000729641 | 0.014448642 |
| GPR171 | 57.78470203 | -1.273875895 | 0.377830028 | -3.371558114 | 0.000747443 | 0.014683344 |
| RP11-567M16.1 | 28.77644216 | -1.520399489 | 0.45132678 | -3.368733155 | 0.000755145 | 0.014781699 |
| C3 | 1207.933365 | -1.273986559 | 0.378603461 | -3.364962795 | 0.00076554 | 0.014917331 |
| SOD3 | 1831.200941 | -1.153640945 | 0.342861677 | -3.364741593 | 0.000766154 | 0.014917331 |
| FCAR | 9.911735786 | 1.271608355 | 0.378137847 | 3.362816938 | 0.000771515 | 0.014992148 |
| NAT8L | 189.6884911 | 1.076649861 | 0.320501213 | 3.359269222 | 0.000781489 | 0.015156126 |
| BMP8A | 393.8519503 | 1.082299993 | 0.322326229 | 3.357778227 | 0.000785716 | 0.015209875 |
| LINC01348 | 12.43534372 | 1.259632711 | 0.375322451 | 3.356134726 | 0.000790401 | 0.015254035 |
| LINC00184 | 3.611968835 | 1.530270581 | 0.456070637 | 3.355336777 | 0.000792684 | 0.015283168 |
| GYG2 | 2755.662009 | 1.008881929 | 0.301129753 | 3.35032297 | 0.000807174 | 0.015479267 |
| MYO5B | 92.17759747 | -1.24651198 | 0.372075547 | -3.350158293 | 0.000807654 | 0.015479267 |
| LRFN5 | 110.0151236 | -1.474377762 | 0.442467041 | -3.33217534 | 0.000861699 | 0.016322192 |
| GPR83 | 24.9954917 | 1.433611668 | 0.430319139 | 3.331508033 | 0.000863768 | 0.016334648 |
| EFCAB8 | 9.44984956 | 1.389034523 | 0.417112623 | 3.330118646 | 0.00086809 | 0.016400668 |
| HPDL | 191.3752949 | 1.009071843 | 0.303137193 | 3.328762908 | 0.000872326 | 0.016447772 |
| LRRN4CL | 98.83078686 | -1.252976647 | 0.376472553 | -3.328201852 | 0.000874085 | 0.016447772 |
| AGAP7P | 14.9316244 | 1.069634406 | 0.321447951 | 3.327550856 | 0.00087613 | 0.016458072 |
| ADORA1 | 37.09069994 | -1.112164989 | 0.334440851 | -3.325446002 | 0.000882772 | 0.016504325 |
| KRT77 | 3.17780844 | 2.074541413 | 0.623689847 | 3.326238874 | 0.000880265 | 0.016504325 |
| CCDC158 | 61.34115666 | 1.342181868 | 0.403684489 | 3.324828929 | 0.000884728 | 0.016525246 |
| RGS9 | 65.54756144 | 1.030349232 | 0.309944754 | 3.324299627 | 0.000886409 | 0.016540996 |
| RP11-554A11.4 | 32.79927065 | -2.017688437 | 0.607301429 | -3.322383811 | 0.000892518 | 0.016623575 |
| AC144831.3 | 62.08927292 | -1.02748929 | 0.309654724 | -3.318177343 | 0.000906069 | 0.016828342 |
| MGAM | 17.72298521 | 1.232246059 | 0.371396117 | 3.317875453 | 0.000907049 | 0.01682932 |
| RP11-20I20.4 | 36.10664891 | -1.31223354 | 0.395785657 | -3.315515648 | 0.000914742 | 0.016909883 |
| SDK1 | 88.28382686 | -1.49450843 | 0.450742842 | -3.315656492 | 0.000914281 | 0.016909883 |
| RGL3 | 193.0142165 | 1.096308682 | 0.330983278 | 3.312278161 | 0.000925395 | 0.017058889 |
| OCA2 | 34.78277453 | 1.550718048 | 0.468215675 | 3.311973797 | 0.000926402 | 0.017061529 |
| CDH16 | 3.07209408 | 1.768510695 | 0.535876528 | 3.300220482 | 0.000966089 | 0.017631801 |
| XKR9 | 65.31800078 | 1.268262606 | 0.384303374 | 3.300159956 | 0.000966297 | 0.017631801 |
| PHOSPHO1 | 7773.733619 | 1.215877925 | 0.3690241 | 3.294846935 | 0.000984753 | 0.017813518 |
| LINC00950 | 26.08906291 | -1.02670907 | 0.311834595 | -3.292479684 | 0.000993081 | 0.017921748 |
| EML5 | 10.4227566 | 1.28924926 | 0.391945378 | 3.289359517 | 0.001004157 | 0.018039186 |
| LINC01353 | 5.834408894 | 1.124969503 | 0.342748384 | 3.282202207 | 0.001029997 | 0.018402926 |
| GBP1P1 | 54.42893667 | -1.40885636 | 0.429326642 | -3.281548878 | 0.001032386 | 0.018422771 |
| ILDR2 | 1104.538441 | 1.259141795 | 0.383823515 | 3.280522806 | 0.001036149 | 0.018462714 |
| MME | 3260.318903 | -1.32928425 | 0.406025163 | -3.273896232 | 0.001060756 | 0.018782509 |
| RP11-416N2.4 | 32.50694007 | 1.113875365 | 0.340336991 | 3.272860124 | 0.001064652 | 0.018793922 |
| PRRT4 | 69.95642981 | -1.294297063 | 0.395691229 | -3.270977392 | 0.001071765 | 0.018892725 |
| ITGA8 | 457.0070858 | -1.001776118 | 0.306452184 | -3.268947557 | 0.001079483 | 0.018994861 |
| CCL16 | 4.374515789 | 1.21395399 | 0.371401535 | 3.268575582 | 0.001080903 | 0.018997714 |
| IP6K3 | 43.5475364 | -1.776096298 | 0.544426668 | -3.262324206 | 0.001105027 | 0.019306677 |
| ACE2 | 18.91211847 | 1.289593861 | 0.395905892 | 3.257324249 | 0.001124679 | 0.019615312 |
| RNF32 | 68.36308426 | 1.005829685 | 0.308891999 | 3.256250366 | 0.001128942 | 0.019637615 |
| CTB-186G2.1 | 3.920614946 | 1.869499427 | 0.574173316 | 3.255984517 | 0.001129999 | 0.019638709 |
| GDF7 | 40.64218222 | -1.181284466 | 0.36284067 | -3.255656175 | 0.001131307 | 0.019644139 |
| COL22A1 | 4288.65463 | 1.448052969 | 0.445332051 | 3.251625312 | 0.001147472 | 0.019889847 |
| HAL | 7.738834823 | 1.073339739 | 0.330145509 | 3.251111134 | 0.001149549 | 0.019908376 |
| SH3BGR | 187.0441677 | -1.00302662 | 0.308651102 | -3.249710157 | 0.001155227 | 0.019989171 |
| GS1-259H13.2 | 22.29323277 | -1.041691739 | 0.320673245 | -3.248452298 | 0.001160347 | 0.020025108 |
| SLC17A7 | 254.8765561 | 1.171082036 | 0.360574045 | 3.247826766 | 0.0011629 | 0.020034157 |
| ISLR | 5593.315175 | -1.770529049 | 0.546507705 | -3.239714707 | 0.001196493 | 0.020469992 |
| MB | 133.3844852 | 1.65034351 | 0.509537263 | 3.238906413 | 0.001199889 | 0.020510317 |
| POU5F1B | 18.65081609 | 1.360608145 | 0.420572653 | 3.235132232 | 0.001215864 | 0.020765402 |
| CNTN1 | 655.9709983 | -1.339809171 | 0.414420127 | -3.232973217 | 0.00122509 | 0.020832869 |
| DSG1 | 5.96070714 | 1.944484819 | 0.601404768 | 3.233238117 | 0.001223955 | 0.020832869 |
| CD8B | 52.57782355 | -1.505331771 | 0.465691834 | -3.232463321 | 0.001227279 | 0.020835329 |
| NTF4 | 5.769177099 | -1.61776014 | 0.500763171 | -3.230589294 | 0.001235353 | 0.02089938 |
| IRF4 | 64.3352157 | -1.264445921 | 0.391432499 | -3.230303881 | 0.001236587 | 0.020902345 |
| SRPK3 | 295.0494967 | -1.06228244 | 0.328886747 | -3.229933854 | 0.001238189 | 0.020911512 |
| CSPG4P13 | 151.3968974 | 1.008990519 | 0.312473906 | 3.229039289 | 0.001242068 | 0.02093783 |
| GOLGA6A | 2.33205407 | 2.044555581 | 0.633377387 | 3.228021119 | 0.001246498 | 0.020962184 |
| BMPR1B | 160.3035347 | -1.29921555 | 0.402646688 | -3.226688776 | 0.001252316 | 0.021042105 |
| ADCY5 | 80.29609619 | -1.092414505 | 0.338803215 | -3.22433335 | 0.001262663 | 0.021143987 |
| FGF7 | 186.1671673 | -1.368155017 | 0.424548811 | -3.222609465 | 0.001270286 | 0.021185666 |
| HLF | 61.44652686 | -1.349154178 | 0.418644163 | -3.222675238 | 0.001269995 | 0.021185666 |
| VGLL2 | 60.22374471 | -2.176432864 | 0.67551853 | -3.221869966 | 0.001273569 | 0.02121863 |
| HGD | 6.458701878 | -1.345407678 | 0.417763881 | -3.220497842 | 0.001279682 | 0.021241069 |
| SLC14A1 | 76.50018982 | -1.27454573 | 0.395720358 | -3.220824264 | 0.001278225 | 0.021241069 |
| SH2D5 | 16.4594429 | -1.47854488 | 0.459252706 | -3.219458177 | 0.001284331 | 0.021290131 |
| RP11-375N15.2 | 33.13390293 | 1.005701918 | 0.312528491 | 3.217952749 | 0.001291091 | 0.021343882 |
| CLDN11 | 417.7133057 | 1.516511299 | 0.472051976 | 3.212593899 | 0.001315421 | 0.021623951 |
| RP11-544A12.8 | 20.68620117 | 1.095532214 | 0.341264633 | 3.210213157 | 0.001326366 | 0.021767613 |
| PDZRN4 | 208.2357971 | -1.517513877 | 0.473449205 | -3.205230595 | 0.001349543 | 0.022056317 |
| TRPM6 | 46.8430209 | 1.217757669 | 0.379923978 | 3.205266682 | 0.001349374 | 0.022056317 |
| UGT3A1 | 4.549964007 | 1.626690334 | 0.507476489 | 3.205449652 | 0.001348516 | 0.022056317 |
| PHEX | 1125.077499 | 1.25755521 | 0.392424716 | 3.204576976 | 0.001352611 | 0.022069919 |
| TRIM72 | 39.66173055 | -1.466120804 | 0.457485371 | -3.204738113 | 0.001351854 | 0.022069919 |
| ERAP2 | 2748.603537 | -1.205181478 | 0.376147919 | -3.204009425 | 0.00135528 | 0.022093676 |
| XPNPEP2 | 337.5060333 | -1.661591257 | 0.519239137 | -3.20005011 | 0.001374037 | 0.022288974 |
| RP11-22P4.1 | 6.113426285 | 1.047850638 | 0.327859414 | 3.196036456 | 0.001393295 | 0.022473713 |
| PLXNB3 | 303.4806451 | -1.044779331 | 0.327033828 | -3.194713332 | 0.001399698 | 0.02254016 |
| SPTB | 104.0639642 | 1.317962339 | 0.412698013 | 3.19352722 | 0.001405461 | 0.022596103 |
| PAX9 | 92.99994206 | -1.359691137 | 0.426246385 | -3.189918286 | 0.00142313 | 0.02282442 |
| FGF9 | 11.5318112 | 1.46630505 | 0.459918692 | 3.188183207 | 0.001431698 | 0.022924584 |
| COL4A3 | 47.93762465 | 1.137396661 | 0.357550026 | 3.181083984 | 0.001467251 | 0.023267417 |
| C10orf71 | 52.12846902 | -2.201633332 | 0.692609381 | -3.178751825 | 0.001479107 | 0.023380308 |
| RP11-384O8.1 | 49.16401919 | -1.410634075 | 0.444206569 | -3.175626324 | 0.001495134 | 0.023558206 |
| EVPL | 21.46349676 | 1.053491933 | 0.331792497 | 3.175152974 | 0.001497575 | 0.023576286 |
| RP11-106D4.2 | 25.06428884 | 1.033221156 | 0.325886773 | 3.170491228 | 0.001521814 | 0.023792995 |
| SLC38A3 | 329.9211015 | -1.020919986 | 0.322153666 | -3.169046619 | 0.001529399 | 0.023850645 |
| FCRL6 | 19.64218821 | -1.107118755 | 0.349680447 | -3.166087107 | 0.001545045 | 0.023999864 |
| RHBDL2 | 2539.522108 | 1.009516964 | 0.318934218 | 3.165282705 | 0.001549323 | 0.024047399 |
| FMOD | 3681.093344 | -1.235207727 | 0.390428359 | -3.163724408 | 0.001557642 | 0.024119631 |
| RP11-613D13.8 | 9.80403685 | 1.307957541 | 0.414728152 | 3.153770817 | 0.001611756 | 0.024666353 |
| LINC01119 | 23.28211868 | -1.036821677 | 0.328905399 | -3.152340095 | 0.001619675 | 0.024731048 |
| ELAVL2 | 35.09140904 | -1.182472166 | 0.375520494 | -3.148888504 | 0.001638927 | 0.024920389 |
| HABP2 | 4.179204419 | 1.464634172 | 0.465146356 | 3.148759855 | 0.001639649 | 0.024920389 |
| XKR4 | 66.2386611 | 1.35030786 | 0.429391196 | 3.144703179 | 0.001662554 | 0.025210298 |
| ASPA | 115.4929489 | -1.42884856 | 0.455293501 | -3.13830212 | 0.001699296 | 0.025629646 |
| CXCL14 | 4605.765575 | -1.314203492 | 0.419206308 | -3.13498024 | 0.001718657 | 0.02586238 |
| RP11-54O7.3 | 644.2097219 | 1.046958238 | 0.334106585 | 3.133605513 | 0.001726728 | 0.02592456 |
| IDO1 | 123.0078038 | -1.305703721 | 0.417091365 | -3.130498084 | 0.001745101 | 0.02616062 |
| GRID1 | 71.41536412 | -1.088810678 | 0.348185553 | -3.127098953 | 0.001765405 | 0.026364894 |
| PLA2G4F | 25.96458136 | 1.592863732 | 0.509522871 | 3.126186914 | 0.00177089 | 0.026426812 |
| ASB18 | 9.390814773 | 1.524595031 | 0.487782273 | 3.125564655 | 0.001774641 | 0.026429768 |
| SLPI | 123.7390088 | -1.416241753 | 0.453126614 | -3.125487907 | 0.001775104 | 0.026429768 |
| FLJ31356 | 19.09692503 | -1.121420067 | 0.359998871 | -3.11506551 | 0.001839041 | 0.027176781 |
| RYR1 | 725.9532129 | -1.174737056 | 0.37752057 | -3.111716682 | 0.001860029 | 0.027425359 |
| FLG | 181.7351251 | -1.124820482 | 0.361887236 | -3.108207118 | 0.001882261 | 0.027655837 |
| PTCHD1 | 101.6985308 | -1.473705104 | 0.474262716 | -3.107360232 | 0.001887662 | 0.027667495 |
| RP11-411K7.1 | 5.378349134 | 1.527532696 | 0.492532349 | 3.101385521 | 0.001926173 | 0.028044573 |
| ASPHD1 | 254.5124397 | 1.111974566 | 0.358726337 | 3.09978513 | 0.001936611 | 0.028155012 |
| GRB14 | 15.92684385 | 1.336874796 | 0.431469794 | 3.098420363 | 0.001945553 | 0.028222662 |
| CTB-12O2.1 | 4.508946798 | 1.528234758 | 0.493454134 | 3.0970148 | 0.001954801 | 0.02825303 |
| PCDHGA10 | 285.2668984 | -1.102733381 | 0.356922555 | -3.089559246 | 0.002004537 | 0.028595071 |
| JPH2 | 234.6355867 | -1.067962633 | 0.346216399 | -3.084667958 | 0.002037795 | 0.028925829 |
| AP001056.1 | 3.935342098 | 1.531808136 | 0.498520008 | 3.072711448 | 0.002121235 | 0.029807503 |
| OPCML | 35.12133983 | 1.523913474 | 0.496337494 | 3.070317057 | 0.002138316 | 0.029962229 |
| COL4A6 | 13.86931445 | 1.267754363 | 0.413383989 | 3.06677181 | 0.00216384 | 0.030234033 |
| PXT1 | 7.283659009 | 1.043645164 | 0.340453931 | 3.065451944 | 0.002173413 | 0.030332637 |
| FAM189A1 | 116.4119911 | -1.231464665 | 0.40189213 | -3.064167154 | 0.002182769 | 0.030390981 |
| TMEM35 | 209.2112605 | 1.351850097 | 0.442108168 | 3.057736081 | 0.002230159 | 0.030704343 |
| NDNF | 3457.504025 | 1.062806181 | 0.348094273 | 3.053213637 | 0.002264047 | 0.031018397 |
| TTLL6 | 19.66670773 | 1.348938717 | 0.441739157 | 3.053699666 | 0.002260382 | 0.031018397 |
| IGJ | 174.2393598 | -1.556884983 | 0.511835216 | -3.041769954 | 0.002351916 | 0.031760514 |
| MSC | 993.0492493 | -1.107662325 | 0.364309674 | -3.040441699 | 0.002362314 | 0.031835827 |
| PCDHGB4 | 69.15352679 | 1.126526373 | 0.370645239 | 3.039365555 | 0.00237077 | 0.031867756 |
| IL20RA | 57.94479197 | -1.424170123 | 0.468667947 | -3.038761519 | 0.002375528 | 0.031904798 |
| RN7SKP70 | 2.148645084 | 1.306557404 | 0.430005291 | 3.038468204 | 0.002377842 | 0.031914163 |
| RP11-77H9.8 | 1.779966359 | 1.906991234 | 0.627870113 | 3.037238424 | 0.002387565 | 0.031979444 |
| NWD1 | 13.71369907 | 1.246497862 | 0.410537004 | 3.036261895 | 0.002395312 | 0.032061456 |
| HES7 | 55.98938897 | 1.282787459 | 0.422677371 | 3.034909238 | 0.002406081 | 0.032161986 |
| SFRP5 | 11.04181055 | -1.848666364 | 0.609940195 | -3.03089775 | 0.002438278 | 0.032429052 |
| ADAMTSL1 | 516.1742923 | -1.108039444 | 0.366679041 | -3.021823775 | 0.002512568 | 0.03311457 |
| LMF1-AS1 | 3.398565942 | -1.22264189 | 0.404632804 | -3.021608425 | 0.002514356 | 0.033116028 |
| C1orf61 | 14.83798078 | 1.057886297 | 0.350733655 | 3.016209829 | 0.002559561 | 0.033423036 |
| STAC2 | 326.7432003 | -1.326726235 | 0.440589394 | -3.011253226 | 0.002601718 | 0.033817663 |
| CH507-254M2.2 | 1.400908396 | -2.002596485 | 0.665764754 | -3.007964107 | 0.002630042 | 0.034138671 |
| LINC01314 | 17.50289027 | 1.121808207 | 0.373158768 | 3.006249094 | 0.002644922 | 0.034219333 |
| LINC01230 | 6.515404873 | 1.676918022 | 0.558018291 | 3.005130923 | 0.002654666 | 0.034300433 |
| FSTL4 | 20.11309217 | -1.411612676 | 0.470704543 | -2.998935739 | 0.002709244 | 0.034821523 |
| CTC-498M16.2 | 3.848634531 | 1.937226446 | 0.647217006 | 2.993163698 | 0.002761016 | 0.035213657 |
| RGS11 | 68.84186137 | -1.379439585 | 0.461323982 | -2.990175318 | 0.002788174 | 0.035425391 |
| RP1-8B1.4 | 3.84817338 | 1.650328108 | 0.551959909 | 2.989941988 | 0.002790304 | 0.035425391 |
| CASQ2 | 37.80974539 | -1.373241929 | 0.460821503 | -2.979986652 | 0.002882609 | 0.036174559 |
| ACSM1 | 4.393471257 | 1.50792011 | 0.507795461 | 2.969542318 | 0.002982437 | 0.036990962 |
| NRAP | 41.94993346 | 1.442736879 | 0.486443695 | 2.965886687 | 0.003018117 | 0.037218412 |
| ADRA1D | 847.3493103 | 1.130597039 | 0.381932762 | 2.960199151 | 0.003074402 | 0.037607141 |
| CNN1 | 125.41946 | -1.11146668 | 0.375536442 | -2.959677292 | 0.003079614 | 0.037634355 |
| LINC00313 | 2.14494984 | -1.584564525 | 0.538004969 | -2.945260017 | 0.003226834 | 0.039144072 |
| SCX | 466.2574372 | -1.162306541 | 0.394674114 | -2.944977894 | 0.003229777 | 0.039144072 |
| CAPN8 | 6.416432573 | 1.455309827 | 0.49432095 | 2.944058565 | 0.003239387 | 0.039164248 |
| NOG | 233.1410201 | 1.085874656 | 0.368922095 | 2.943371165 | 0.003246589 | 0.039174001 |
| PROX1-AS1 | 17.42598792 | 1.474062179 | 0.500832439 | 2.943224246 | 0.00324813 | 0.039174001 |
| COCH | 569.4057309 | 1.498061936 | 0.509268556 | 2.941595194 | 0.003265265 | 0.039284656 |
| OVCH1 | 9.648077616 | 1.114919515 | 0.379121954 | 2.940793859 | 0.003273723 | 0.039362434 |
| ARHGDIG | 12.88928877 | -1.768733253 | 0.601543187 | -2.940326299 | 0.003278668 | 0.039397892 |
| MYL1 | 115.3504787 | -1.876009175 | 0.638222769 | -2.939426898 | 0.003288198 | 0.039470849 |
| MFAP4 | 3808.922907 | -1.79518678 | 0.611095209 | -2.93765481 | 0.00330705 | 0.039546391 |
| ST6GALNAC1 | 11.15049677 | 1.135171294 | 0.386746519 | 2.935181672 | 0.003333525 | 0.039838847 |
| C1orf167 | 4.492629802 | 1.425965832 | 0.486010172 | 2.934024667 | 0.003345976 | 0.039915171 |
| SFRP4 | 7429.306663 | -1.134050191 | 0.386702979 | -2.932613017 | 0.003361226 | 0.040041368 |
| GRK7 | 13.55548426 | 1.093262939 | 0.373569262 | 2.926533444 | 0.003427627 | 0.040643138 |
| KCNJ6 | 53.34302857 | -1.376421431 | 0.470423891 | -2.925917366 | 0.003434421 | 0.04066276 |
| SYT1 | 118.0142454 | -1.205331461 | 0.411962658 | -2.925826988 | 0.003435419 | 0.04066276 |
| ASB5 | 157.6223719 | -1.350372294 | 0.463032105 | -2.916368603 | 0.003541319 | 0.041542633 |
| APOL5 | 7.311053383 | 1.180230813 | 0.405214998 | 2.912603973 | 0.003584289 | 0.041947012 |
| OSTN | 162.5202225 | -2.303734595 | 0.792279553 | -2.907729458 | 0.003640631 | 0.042415209 |
| COL25A1 | 202.1358177 | -1.40628922 | 0.485790652 | -2.894846196 | 0.003793444 | 0.043503478 |
| NELL2 | 42.75107645 | -1.151357317 | 0.397922394 | -2.893421769 | 0.003810692 | 0.043639497 |
| CA3 | 7879.847549 | 1.416773895 | 0.489983117 | 2.891474921 | 0.003834382 | 0.043688152 |
| DUSP13 | 24.67725629 | -1.898400368 | 0.656559693 | -2.891436053 | 0.003834856 | 0.043688152 |
| CR1 | 41.81017444 | 1.140124439 | 0.395352924 | 2.883814357 | 0.003928903 | 0.044451765 |
| CAMK2N1 | 651.4790497 | -1.012852333 | 0.351468225 | -2.881774972 | 0.00395442 | 0.044673863 |
| RP11-214C8.2 | 4.785113082 | 1.03366854 | 0.359371867 | 2.876320145 | 0.004023414 | 0.045220754 |
| PTPRQ | 15.49232128 | -1.508391193 | 0.524769638 | -2.874387318 | 0.004048122 | 0.045358724 |
| RP11-236L14.2 | 6.993534759 | 1.42304966 | 0.495276811 | 2.873241041 | 0.00406284 | 0.045395388 |
| ACTA1 | 332.1142221 | -1.413168607 | 0.492881775 | -2.86715533 | 0.004141797 | 0.045899886 |
| NEFM | 31.03560522 | -1.285063185 | 0.448060166 | -2.868059431 | 0.004129979 | 0.045899886 |
| MYLPF | 98.91905828 | -1.199725311 | 0.418672747 | -2.865544318 | 0.00416293 | 0.045951867 |
| NFE2 | 81.84954413 | 1.008899771 | 0.352091464 | 2.865447975 | 0.004164197 | 0.045951867 |
| RP11-141M3.6 | 3.794789977 | 1.206092856 | 0.420931687 | 2.865293569 | 0.004166228 | 0.045951867 |
| RP11-883A18.3 | 20.24892154 | -1.543128811 | 0.53905246 | -2.862669085 | 0.004200891 | 0.04623087 |
| SPON1 | 1635.682754 | -1.116450412 | 0.390237047 | -2.860954437 | 0.004223677 | 0.046404043 |
| EXTL1 | 283.1292468 | -1.012525948 | 0.354238883 | -2.85831397 | 0.004258988 | 0.046713995 |
| FLJ38122 | 5.788101426 | 1.153963401 | 0.403828717 | 2.857556563 | 0.004269165 | 0.046773658 |
| AC092159.2 | 8.311308215 | -1.062078403 | 0.372198809 | -2.853524451 | 0.00432372 | 0.047240291 |
| RP4-533D7.4 | 10.08515271 | 1.036847868 | 0.363943285 | 2.848927045 | 0.004386694 | 0.047757156 |
| CHI3L2 | 204.356133 | -1.173133166 | 0.412744958 | -2.842271345 | 0.004479335 | 0.048477817 |
| KCNJ16 | 11.42773001 | 1.351963077 | 0.476753621 | 2.835768869 | 0.004571552 | 0.049276144 |
| TLR9 | 1.427544489 | 1.337278181 | 0.472124332 | 2.832470369 | 0.004618985 | 0.049543898 |
| SMPX | 31.35773896 | -2.348900958 | 0.830263834 | -2.829101861 | 0.004667884 | 0.049950153 |
| SLC36A2 | 823.2288683 | 1.164587083 | 0.411672982 | 2.828913078 | 0.004670638 | 0.049952552 |
